# Photochemistry of Benzotriazoles: Generation of 1,3-Diradicals and Intermolecular Cycloaddition as a New Route toward Indoles and Dihydropyrrolo[3,4-*b*]Indoles

**DOI:** 10.3390/molecules191220695

**Published:** 2014-12-11

**Authors:** Nader A. Al-Jalal, Maher R. Ibrahim, Nouria A. Al-Awadi, Mohamed H. Elnagdi, Yehia A. Ibrahim

**Affiliations:** Department of Chemistry, Faculty of Science, Kuwait University, P.O. Box 5969, Safat 13060, Kuwait; E-Mails: maherriyad@hotmail.com (M.R.I.); n.alawadi@ku.edu.kw (N.A.A.-A.); shelmy1941@yahoo.com (M.H.E.); yehiaibrahim2002@yahoo.com (Y.A.I.)

**Keywords:** photolysis, benzotriazoles, maleimides, alkynes, indoles, pyrrolo[3,4-*b*]indoles

## Abstract

Irradiation of benzotriazoles **1a**–**e** at λ = 254 nm in acetonitrile solution generated the corresponding 1,3-diradicals which underwent intermolecular cycloaddition with maleimides to afford the corresponding dihydropyrrolo[3,4-*b*]indoles and with acetylene derivatives to afford indoles as the major products. This offers an interesting and simple access to such ring systems of potential synthetic and biological interest. The structures of the photoproducts were established spectroscopically and by single crystal X-ray crystallography.

## 1. Introduction

**Scheme 1:**
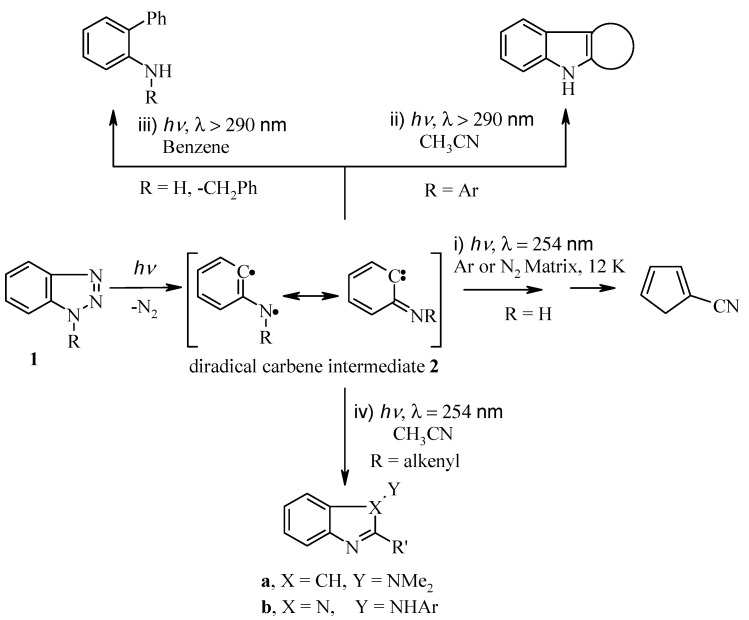
Literature photolysis of benzotriazole derivatives.

The photochemistry of benzotriazoles **1** has been extensively studied in the past [1,2,3,4,5]. Katritzky *et al.*, has already reviewed this chemistry in several review articles [6,7]. Scheme 1 summarizes the photolytic reactions of benzotriazoles which occur through initial extrusion of molecular nitrogen and formation of the diradical intermediate **2**, followed by subsequent rearrangement to: (i) cyanocyclopentadiene [8,9]; (ii) ring closure to condensed heterocyclic products [10,11,12]; (iii) reaction with solvent to yield 2-aminobiphenyl [13,14]; and (iv) intramolecularphotocycloaddtion to produce indoles and benzimidazoles [15,16].

In the present work, we describe intermolecular trapping of the photochemically generated diradical intermediate **2** and its derivatives with alkenes or alkynes. To the best of our knowledge this intermolecular reaction has not been reported before and is expected to offer a new and simple route to indole and dihydropyrrolo[3,4-*b*]indolederivatives. These compounds are structurally similar to 1,2-dihydropyrrolo[3,4-*b*]indol-3(4*H*)-one and its derivatives which have been reported as a vital class of pharmaceutical moieties such as mG1uR1 antagonists [17], cannabinoid 2 receptor agonists [18], potent inhibitor of purified human rennin [19] and therapeutic agents for the treatment of osteoporosis [20].

## 2. Results and Discussion

**Scheme 2:**
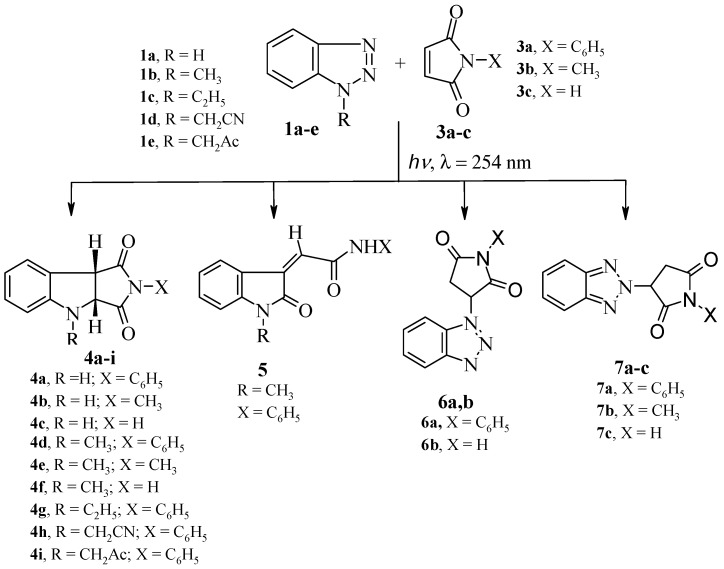
Products of irradiation of benzotriazoles **1a**–**e** with maleimides **3a**–**c**.

**Figure 1:**
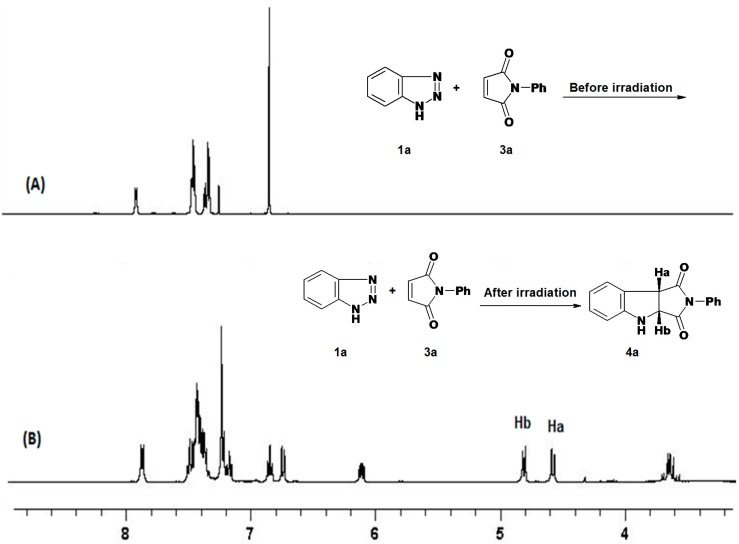
^1^H-NMR of: (**A**) reaction mixture of benzotriazole (**1a**) with *N*-phenylmaleimide (**3a**) before irradiation, and (**B**) reaction mixture after irradiation using low pressure Hg-lamp (λ = 254 nm) in CH_3_CN.

Irradiation of acetonitrile solution of benzotriazole (**1a**) and *N*-phenylmaleimide (**3a**) using a 16 W low pressure mercury arc-lamp (λ = 254 nm) led to the formation of three products **4a**, **6a** and **7a** (Scheme 2). The major product of this reaction was 2-phenyl-3a,4-dihydropyrrolo[3,4-*b*]indole-1,3(2*H*, 8b*H*)-dione (**4a**, Figure 1).

Several experiments were carried out in order to optimize the formation of **4a**, and the results are summarized in Table 1. From this table it is clear that the best reaction condition for the formation of the pyrroloindole **4a** is achieved by irradiating **1a** and **3a** (1:2 molar ratio) in acetonitrile for 16 h. The formation of **4a** in the reaction mixture was monitored by the Ha and Hb proton signals, which appear as two doublet signals at δ: 4.85, 4.61 ppm (*J =* 9.2 Hz) in the ^1^H-NMR spectra as shown in Figure 1. The percent yield of **4a** in the reaction mixture was calculated by ^1^H-NMR using DCM as calibration reference, as reported previously [21].

**Table 1:** Optimizing photolysis condition of benzotriazole (**1a**) with *N*-phenylmaleimide (**3a**).

Run *	Solvent	Time(h)	Molar Ratio (1a):(3a)	Yield (%)
				4a
1	MeOH	24	1:1	-
2	DCM	24	1:1	5
3	THF	24	1:1	8
4	MePh	24	1:1	7
5	MeCN	24	1:1	12
6	MeCN	24	1:2	19
7	MeCN	16	1:2	19
8	MeCN	10	1:2	12
9	MeCN	36	1:2	19

Note: * Reaction conditions: Acetonitrile (25 mL), **1a** (0.0595 g, 0.5 mmol) and **3a** (0.173 g, 1 mmol) irradiated at λ = 254 nm under nitrogen.

With the optimized reaction conditions in hand, irradiation of benzotriazole (**1a**) with maleimides **3a**–**c** produced the dihydropyrrolo[3,4-*b*]indoles **4a**–**c** in 19%–21% yield, together with Michael adducts **6a**,**b** in 17%–18% and **7a**–**c** in 13%–16% yields, as shown in Scheme 2 and Table 2.

**Table 2:** Products from irradiation of benzotriazoles **1a**–**e** with maleimides **3a**–**c**.

Entry *	Reactant		Products (Yield %)				
			4	5	6a,b	7a–c	Unreacted Bt
1	**1a**	**3a**	**4a** (19)	-	**6a**(17)	**7a** (13)	45
2	**1a**	**3b**	**4b** (21)	-	-	**7b**(16)	58
3	**1a**	**3c**	**4c** (19)	-	**6b**(18)	**7c**(15)	43
4	**1b**	**3a**	**4d**(32)	(20)	-	-	42
5	**1b**	**3b**	**4e** (33)	-	-	-	60
6	**1b**	**3c**	**4f**(35)	-	-	-	60
7	**1c**	**3a**	**4g** (38)	-	-	-	56
8	**1d**	**3a**	**4h**(25)	-	-	-	70
9	**1e**	**3a**	**4i**(35)	-	-	-	60

Note: *: Reaction conditions: In acetonitrile (100 mL), **1a**–**e** (2 mmol) and **3a**–**c** (4 mmol) was irradiated at λ = 254 nm under nitrogen for 16 h.

**Figure 2:**
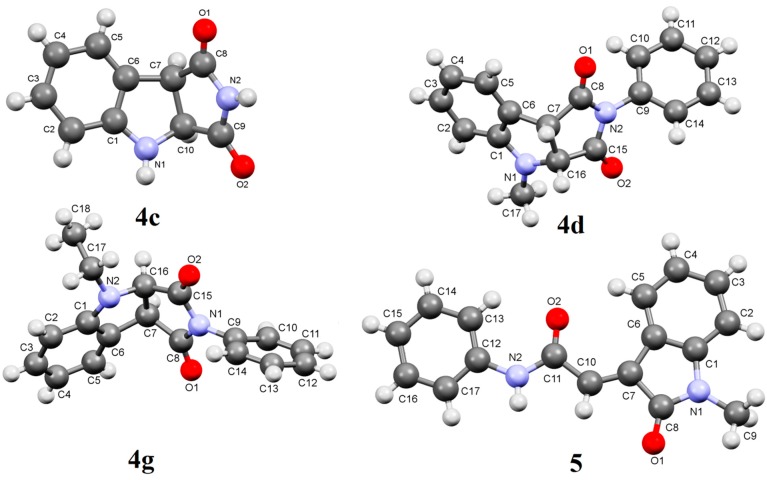
X-Ray crystal structures of compounds **4c**, **4d** and **5**.

The ^1^H-NMR spectrum (CDCl_3_) of pure **4a** showed two doublet signals at δ 4.85, 4.61 (*J =* 9.2 Hz) corresponding to H-b, H-a respectively (Figure 1). The ^13^C-NMR also showed two signals at δ 60.2 and 48.9 assigned to C-b, C-a respectively (HSQC). In addition GC-MS of **4a** showed a M^+^ peak at *m/z* = 264. These data support the structure of **4a**. Moreover, the structure of **4c** was unequivocally established by single crystal X-ray crystallography (Figure 2). The structures of compounds **6a**,**b** and **7a**–**c** were established by spectroscopic data and are the result of ground state Michael addition of **1a** and its tautomer to **3a**–**c**. In support of this view, heating of benzotriazole **1a** and *N*-phenylmaleimide **3a** in acetonitrile under reflux for 24 h gave a mixture of **6a** (24%) and **7a** (16%). On the other hand heating a mixture of **1a** and **3a** in a sealed tube at 230–300 °C produced only a mixture of Michael adducts **6a** (80%) and **7a** (10%) yields. Similar Michael adducts have also been reported recently [22].

The formation of Michael adducts **6a**,**b** and **7a**–**c** and the low yield of dihydropyrroloindoles **4a**–**c** promotedus to investigate the effect of substituting *N*-1 of benzotriazole with various alkyl groups [23]. Thus, irradiation of 1-methyl-1*H*-benzotriazole (**1b**) with maleimides **3a**–**c** produced dihydropyrrolo[3,4-*b*]indoles **4d**–**f** in 32%–35%. Similarly, irradiation of benzotriazoles **1c**–**e** with *N*-phenylmaleimide **3a** produced also the corresponding dihydropyrroloindoles **4g**–**i** in 25%–38% yields.

**Scheme 3:**
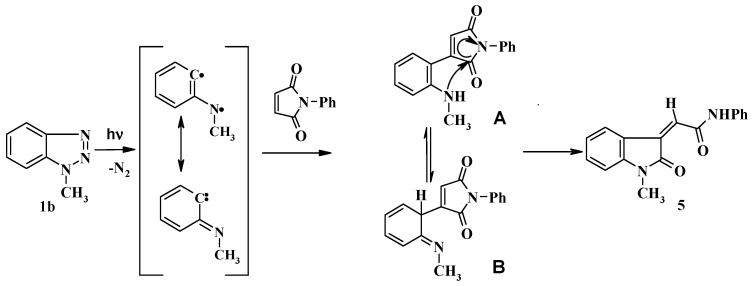
Proposed mechanism for formation of compound **5**.

Irradiation of **1b** with **3a** was accompanied by another photoproduct in 20% yield which was identified as 2-(1-methyl-2-oxoindoline-3-ylidene)-*N*-phenylacetamide (**5**). The formation of compound **5** could be explained as shown in Scheme 3. Thus, C-H insertion of the photogenerated diradicals or its tautomeric carbene intermediates leads to the formation of the intermediate product **A** or its tautomer **B** respectively, followed by nucleophilic transamidation leading to the final product **5**. The structures of **4d**, **4g** and **5** were established from single crystal X-ray crystallography (Figure 2).

In a similar way, irradiation of benzotriazole **1a** with phenylacetylene (**8a**), cyclohexen-1-yl acetylene (**8b**) or ethyl propiolate (**8c**) in acetonitrile under the same conditions afforded the functionally substituted-1*H*-indole derivatives **9a**–**c** in 31%–37% yield. Interestingly, the reaction took place regioselectively depending on the monosubstituted alkyne. Thus, only 2-substituted indole is formed with phenylacetylene and cyclohexen-1-ylacetylene, whereas a 3-substituted indole is formed with ethyl propiolate. In the latter case, the reaction presumably proceeds by initially formation of Michael adduct (3-benzotriazol-1-ylacrylic acid ethyl ester) followed by photolytic extrusion of nitrogen and intramolecular radical cyclization to give **9c**. The latter has been previously prepared from the same Michael adducts by pyrolytic extrusion of nitrogen and intramolecular cyclization [24]. This presumption has now been further supported by the formation of the ethyl *N*-methylindole-2-carboxylate **9f** upon irradiation of **1b** with ethyl propiolate.

**Scheme 4:**
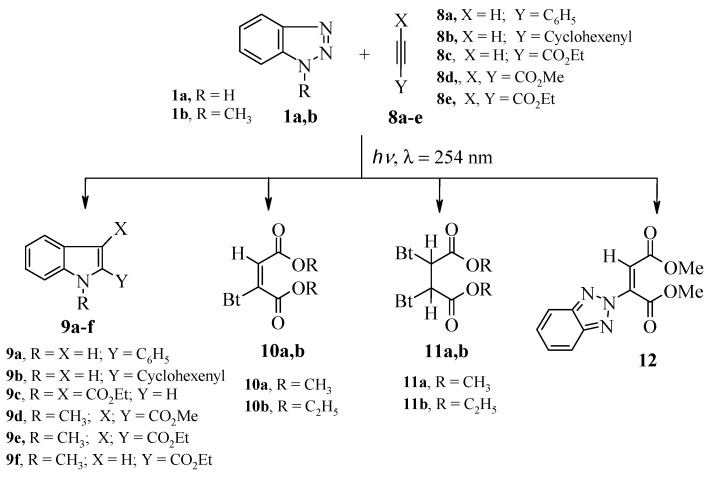
Products of irradiation of benzotriazoles **1a**,**b** with alkynes **8a**–**e**.

On the other hand, irradiation of **1a** with dimethyl and diethyl acetylenedicarboxylates (DMADC) **8d** and (DEADC) **8e**, gave only the corresponding Michael adducts **10a**,**b**, **11a**,**b** and **12**. However, irradiating of 1-methyl-1*H*-benzotriazole (**1b**) with **8d**,**e** produced the corresponding indoles **9d**,**e** Scheme 4, Table 3.

**Table 3:** Products from irradiation of benzotriazoles **1a**,**b** with alkynes **8a**–**e**.

Entry	Reactants		Products (Yield %)				
			9a–f	10a,b	11a,b	12	Unreacted Bt
1	**1a**	**8a**	**9a** (31)	-	-	-	60
2	**1a**	**8b**	**9b** (36)	-	-	-	48
3	**1a**	**8c**	**9c **(37)	-	-	-	50
4	**1a**	**8d**	-	**10a **(25)	**11a **(29)	**12** (19)	5
5	**1a**	**8e**	-	**10b** (31)	**11b** (28)	-	25
6	**1b**	**8d**	**9d** (32)	-	-	-	55
7	**1b**	**8e**	**9e** (23)	-	-	-	61
8	**1b**	**8c**	**9f** (19)	-	-	-	70

Note: Reaction conditions: In acetonitrile (100 mL) **1a**, **b** (2 mmol) and **8a**–**e** (4 mmol) was irradiated at λ = 254 nm under nitrogen for 16 h.

## 3. Experimental Section

### 3.1. General Information

All melting points were recorded on a Gallenkamp apparatus and were uncorrected. IR spectra were recorded using KBr pellets on a Perkin Elmer System 2000 FT-IR spectrophotometer. ^1^H- and ^13^C-NMR spectra were recorded on a Bruker DPX 400MHz or Avance 600 MHz spectrometer with proton spectra measured at 400, 600 MHz and carbon spectra at 100, 150 MHz. Mass spectra were measured on a VG Auto-spec-Q (high resolution, high performance, tri-sector GC/MS/MS) and with LCMS using Agilent 1100 series LC/MSD with an API-ES/APCI ionization mode. Microanalyses were performed on LECO CH NS-932 Elemental Analyzer. X-ray analysis was performed using a Rigaku Rapid II and Bruker X8 Prospector diffractometer [25].

### 3.2. Irradiation of Benzotriazoles **1a**–**e** with Maleimides **3a**–**c** orAlkynes **8a**–**e**

General procedure: A mixture of each benzotriazole derivative **1a**–**e** (2 mmol), the appropriate maleimide **3a**–**c** or alkyne **8a**–**e** (4 mmol) was dissolved in acetonitrile (100 mL) in a quartz tube and purged with nitrogen for 20 min, while being irradiated for 16 h at room temperature using an annular reactor model APQ40 (Applied Photo-Physics Limited, Surrey, UK) fitted with a 16 W low pressure mercury arc-lamp (λ = 254 nm). The progress of reaction was monitored by TLC. The solvent was removed under reduced pressure and the resulting residue was subject to column chromatography on silica gel (70–230 mesh) using ethyl acetate/petroleum ether (bp. 60–80 °C) as an eluent to give the corresponding products. All yields reported in the Experimental are isolated yields.

### 3.3. Products from Irradiation of Benzotriazoles **1a**–**e** with Maleimides **3a**–**c**

*2-Phenyl-3a,4-dihydropyrrolo[3,4-b]indole-1,3(2H,8bH)-dione* (**4a**). This compound was separated as white needles by column chromatography using ethyl acetate/pet. ether (1:4, *R_f_* = 0.56), yield (95 mg), mp.164–165 °C; LCMS (*m/z*) = 265 (M + 1); MS: (*m/z*, %) = 264 (M^+^, 60), 144 (15), 117 (100); IR ν*_max_* (KBr)/cm^−1^ 3257, 3046, 2949, 1775, 1715, 1494, 1452, 1265, 1196, 739. NMR δ*_H_* (600 MHz, CDCl_3_) 7.48–7.45 (m, 5H), 7.40 (dt, 1H, *J =* 8.4, 1.2), 7.26 (t, 1H, *J =* 8.0), 7.21 (dd, 1H, *J =* 8.0, 1.0), 6.89 (dt, 1H, *J =* 8.4, 1.2), 6.78 (d, 1H, *J =* 8.4), 4.85 (d, 1H, *J =* 9.0), 4.61 (d, 1H, *J =* 9.0); δ*_C_* (150 MHz, CDCl_3_) 176.9, 174.8, 149.0, 131.5, 130.0, 129.2, 128.8, 126.3, 125.5, 122.7, 120.5, 110.8, 60.2, 48.9; HR-MS (EI)* m/z* [M]^+^ calcd for C_16_H_12_N_2_O_2_ 264.0899, found = 264.0892.

*2-Methyl-3a,4-dihydropyrrolo[3,4-b]indole-1,3(2H,8bH)-dione* (**4b**). White needles (85 mg) after column chromatography using ethyl acetate/pet. ether (1:6, *R_f_* = 0.64), mp. 148–150 °C; MS: (*m/z*, %) = 202 (M^+^, 15), 119 (100), 91 (90); IR ν*_max_* (KBr)/cm^−1^ 3370, 2957, 2928, 1773, 1713, 1598, 1484, 1394, 1188, 750; NMR δ*_H_* (400 MHz, CDCl_3_) 7.43 (d, 1H, *J =* 8.0), 7.17 (t, 1H, *J =* 8.4), 6.87 (dt, 1H, *J* = 8.4, 1.2), 6.74 (d, 1H, *J =* 8.0), 4.70 (d, 1H, *J =* 8.8), 4.47 (d, 1H, *J =* 8.8), 3.1 (br, 1H, NH), 3.02 (s, 3H, CH_3_); δ*_C_* (100 MHz, CDCl_3_) 178.0, 176.1, 149.2, 130.0, 125.7, 123.0, 120.5, 110.9, 60.3, 49.1, 25.4; HR-MS (EI)* m/z* [M]^+^ calcd for C_11_H_10_N_2_O_2_ 202.0742, found 202.0736.

*3a,4-Dihydropyrrolo[3,4-b]indole-1,3(2H,8bH)-dione* (**4c**). A white solid (72 mg) from column chromatography using ethyl acetate/pet. ether (1:4, *R_f_* = 0.64), mp. 165–167 °C; MS: (*m/z*, %) = 188 (M^+^, 85), 118 (100), 91 (60); IR ν*_max_* (KBr)/cm^−1^ 3380, 3360, 3087, 2958, 1765, 1711, 1600, 1480, 1344, 1187, 1051, 794; NMR δ*_H_* (400 MHz, CDCl_3_) 8.18 (br, 2H, 2NH), 7.39 (d, 1H, *J =* 7.2), 7.17 (t, 1H, *J =* 7.6), 6.83 (t, 1H, *J =* 7.2), 6.72 (d, 1H, *J =* 7.6), 4.68 (d, 1H, *J =* 9.2), 4.46 (d, 1H, *J =* 9.2); δ*_C_* (100 MHz, CDCl_3_) 178.0, 175.8, 149.3, 130.1, 125.6, 122.6, 120.6, 110.9, 61.5, 50.3; HR-MS (EI) *m/z* [M]^+^ calcd for C_10_H_8_N_2_O_2_ 188.0586, found 188.0580.

*4-Methyl-2-phenyl-3a,4-dihydropyrrolo[3,4-b]indole-1,3(2H,8bH)-dione* (**4d**)*.* A white solid (178 mg) from column chromatography using ethyl acetate/pet. ether (1:3, *R_f_* = 0.62), mp. 146–148 °C; MS: (*m/z*, %) = 278 (M^+^, 100), 183 (15), 156 (100); IR ν*_max_* (KBr)/cm^−1^ 3044, 2893, 1774, 1711, 1600, 1491, 1379, 1293, 1187, 741; NMR δ*_H_* (400 MHz, CDCl_3_) 7.46–7.42 (m, 4H), 7.30–7.25 (m, 3H), 6.80 (t, 1H, *J =* 7.8), 6.54 (d, 1H, *J =* 7.6), 4.60 (d, 1H *J =* 9.6), 4.56 (d, 1H, *J =* 9.6), 3.16 (s, 3H); δ*_C_* (100 MHz, CDCl_3_) 174.7, 174.6, 150.9, 131.6, 130.0, 129.1, 128.7, 126.4, 125.5, 122.1, 118.6, 107.1, 66.3, 48.1, 34.0; HR-MS (EI)* m/z* [M]^+^ calcd for C_17_H_14_N_2_O_2_ 278.1055, found 278.1050.

*2,4-Dimethhyl-3a,4-dihydropyrrolo[3,4-b]indole-1,3(2H,8bH)-dione* (**4e**). A white solid (143 mg) from column chromatography using ethyl acetate/pet. ether (1:4, *R_f_* = 0.53) mp. 98–100 °C; MS: (*m/z*, %) = 216 (M^+^, 100), 158 (45), 131 (85); IR ν*_max_* (KBr)/cm^−1^ 3044, 2893, 1774, 1711, 1600, 1491, 1379, 1293, 1187, 741; NMR δ*_H_* (400 MHz, CDCl_3_) 7.37 (d, 1H, *J =* 7.2), 7.21 (t, 1H, *J =* 7.6), 6.75–6.70 (m, 1H), 6.46 (d, 1H, *J =* 8.0), 4.42 (d, 1H, *J =* 9.6), 4.39 (d, 1H, *J =* 9.6), 3.09 (s, 3H, CH_3_), 2.98 (s, 3H, CH_3_); δ*_C_* (100 MHz, CDCl_3_) 176.0, 175.8, 150.9, 130.1, 125.6, 122.3, 118.5, 107.1, 66.3, 48.2, 34.1, 23.9; HR-MS (EI) *m/z* [M]^+^ calcd for C_12_H_12_N_2_O_2_ 216.0899, found 216.0893.

*4-Methyl-3a,4-dihydropyrrolo[3,4-b]indol-1,3(2H,8bH)-dione* (**4f**). A white solid (142 mg) from column chromatography using ethyl acetate/pet. ether (1:5, *R_f_* = 0.44), mp. 155–157 °C; MS: (*m/z*, %) = 202 (M^+^, 95), 158 (15), 131 (100); IR ν*_max_* (KBr)/cm^−1^ 3274, 3051, 2958, 1772, 1711, 1605, 1487, 1341, 1198, 746; NMR δ*_H_* (400 MHz, CDCl_3_) 8.02 (br, 1H, NH), 7.35 (d, 1H, *J =* 7.2), 7.22 (t, 1H, *J =* 7.6), 6.75 (t, 1H, *J =* 7.8), 6.48 (d, 1H,*J* =8.0), 4.45 (d, 1H, *J =* 9.2), 4.39 (d, 1H, *J =* 9.2), 3.08 (s, 3H, CH_3_); δ*_C_* (100 MHz, CDCl_3_) 175.7, 175.6, 151.0, 130.2, 125.6, 122.0, 118.7, 107.2, 67.5, 49.5, 34.1; HR-MS (EI)* m/z* [M]^+^ calcd for C_11_H_10_N_2_O_2_ 202.0742, found 202.0735.

*4-Ethyl-2-phenyl-3a,4-dihydropyrrolo[3,4-b]indol-1,3(2H,8bH)-dione* (**4g**). A white solid (223 mg) from column chromatography using ethyl acetate/pet. ether (1:3, *R_f_* = 0.74), mp. 155–156 °C; MS: (*m/z*, %) = 292 (M^+^, 100), 277 (65), 130 (70); IR ν*_max_* (KBr)/cm^−1^ 3055, 2953, 1776, 1706, 1603, 1467, 1348, 1218, 756;NMR δ*_H_* (600 MHz, CDCl_3_) 7.46–7.44 (m, 3H), 7.39 (t, 1H, *J* = 7.4), 7.28–7.21 (m, 2H), 7.22 (t, 1H, *J =* 7.2), 6.77 (t, 1H, *J =* 7.8), 6.55 (d, 1H, *J =* 7.6), 4.69 (d, 1H, *J =* 9.6), 4.59 (d, 1H, *J =* 9.6), 3.61 (m, 1H), 3.56 (m, 1H), 1.29 (t, 3H, *J =* 7.2); δ*_C_* (150 MHz, CDCl_3_) 175.1, 174.8, 149.9, 131.7, 129.9, 129.1, 128.7, 126.4, 125.6, 122.3, 118.4, 107.4, 63.9, 48.1, 41.6, 11.6; HR-MS (EI)* m/z* [M]^+^ calcd for C_18_H_16_N_2_O_2_ 292.1212, found 292.1207.

*2-(1,3-Dioxo-2-phenyl-1,2,3,3a-tetrahydropyrrolo[3,4-b]indol-4(8bH)-yl)-acetonitrile* (**4h**)*.* A pale yellow solid (154 mg) from column chromatography using ethyl acetate/pet. ether (1:4, *R_f_* = 0.55), mp. 165–167 °C; MS: (*m/z*, %) = 303 (M^+^, 100), 183 (15), 156 (100); IR ν*_max_* (KBr)/cm^−1^ 3060, 2960, 2246, 1768, 1717, 1601, 1487, 1382, 1194, 1173, 751; NMR δ*_H_* 600 MHz, CDCl_3_) 7.53 (d, 1H, *J =* 7.8), 7.47–7.44 (m, 2H), 7.41–7.38 (m, 2H), 7.34 (t, 1H, *J =* 7.8), 7.29–7.27 (m, 1H), 6.98 (t, 1H, *J =* 7.2), 6.68 (d, 1H, *J =* 7.8), 4.72 (d, 1H, *J =* 9.0), 4.67 (d, 1H, *J =* 9.0), 4.51 (d, 1H, *J =* 18.6), 4.42 (d, 1H, *J =* 18.6); δ*_C_* (100 MHz, CDCl_3_) 174.2, 174.0, 147.6, 131.4, 130.5, 129.5, 129.2, 126.5, 126.4, 123.1, 121.9, 114.9, 108.7, 64.3, 48.2, 36.4; HR-MS (EI)* m/z* [M]^+^ calcd for C_18_H_13_N_3_O_2_ 303.1008, found 303.1002.

*4-(2-Oxopropyl)-2-phenyl-3a,4-dihydropyrrolo[3,4-b]indole-1,3(2H,8bH)-dione* (**4i**). A white solid (224 mg) from column chromatography using ethyl acetate/pet. ether (1:2, *R_f_* = 0.44), mp. 98–100 °C; MS: (*m/z*, %) = 320 (M^+^, 20), 277 (100), 130 (35); IR ν*_max_* (KBr)/cm^−1^ 3052, 2957, 1777, 1714, 1600, 1489, 11245, 1168, 747; NMR δ*_H_* (400 MHz, CDCl_3_) 7.39–7.35 (m, 4H), 7.28–7.25 (m*,* 2H), 7.18 (t, 1H, *J =* 7.2), 6.80 (t, 1H, *J =* 7.2), 6.33 (d, 1H, *J =* 7.6), 4.77 (d, 1H, *J =* 9.6), 4.65 (d, 1H, *J =* 9.6), 4.40 (d, 1H, *J =* 18.8), 4.31 (d, 1H, *J =* 18.8), 2.21 (s, 3H); δ*_C_* (150 MHz, CDCl_3_) 204.7, 174.90, 174.86, 149.7, 131.7, 130.1, 129.4, 129.0, 126.6, 126.0, 122.3, 119.5, 106.9, 64.3, 55.7, 48.4, 27.5; HR-MS (EI)* m/z* [M]^+^ calcd for C_19_H_16_N_2_O_3_ 320.1161, found 320.1155.

*2-(1-Methyl-2-oxoindolin-3-ylidene)-N-phenylacetamide* (**5**). Yellow needles (110 mg) from column chromatography using ethyl acetate/pet. ether (1:1, *R_f_* = 0.58), mp. 225–227 °C; MS: (*m/z*, %) = 278 (M^+^, 70), 186 (100), 146 (35); IR ν*_max_* (KBr)/cm^−1^ 3325, 3051, 2858, 1701, 1671, 1603, 1540, 1369, 1244, 1189, 741; NMR δ*_H_* (400 MHz, CDCl_3_) 8.73 (d, 1H, *J =* 7.6), 8.16 (br, 1H, NH), 7.68 (d, 2H, *J =* 8.0), 7.41–7.35 (m, 3H), 7.18 (t, 1H, *J =* 7.8), 7.11–7.00 (m, 2H), 6.80 (d, 1H, *J* = 8.0), 3.56 (s, 3H); δ*_C_* (150 MHz, CDCl_3_) 168.4, 162.6, 145.7, 137.9, 136.5, 132.3, 129.6, 129.4, 129.2, 126.2, 125.3, 123.3, 120.4, 108.3, 26.6. HR-MS (EI)* m/z* [M]^+^ calcd for C_17_H_14_N_2_O_2_ 278.1055, found 278.1050.

*3-(1H-Benzotriazol-1-yl)-1-phenylpyrrolidine-2,5-dione* (**6a**). A colorless powder (100 mg) from column chromatography using ethyl acetate/pet. ether (1:3, *R_f_* = 0.65), mp. 146–148 °C (145–147 °C, [22]); MS: (*m/z*,%) = 292 (M^+^, 80), 236 (15), 173 (100); ν*_max_* (KBr)/cm^−1^ 2980, 2936, 2852, 1717, 1595, 1499, 1395, 1186, 840, 748; NMR δ*_H_* (400 MHz, CDCl_3_) 8.14 (d, 1H, *J =* 8.4), 7.63–7.57 (m, 2H), 7.53–7.42 (m, 4H), 7.38 (dd, 2H, *J =* 8.4, 1.6), 5.98 (dd, 1H, *J =* 9.6, 5.6), 3.86 (dd, 1H, *J =* 18.4, 5.6), 3.65 (dd, 1H, *J =* 18.4, 5.6); δ*_C_* (100 MHz, CDCl_3_) 172.2, 170.9, 146.5, 133.2, 131.3, 129.6, 129.5, 128.8, 126.5, 124.9, 120.9, 109.2, 55.8, 35.1; HR-MS (EI)* m/z* [M]^+^ calcd for C_16_H_12_N_4_O_2_ 292.0960), found 292.0951.

*3-(1H-Benzotriazol-1-yl)-pyrrolidine-2,5-dione* (**6b**). A colorless powder (78 mg) from column chromatography using ethyl acetate/pet. ether (1:2, *R_f_* = 0.58), mp. 216–218 °C; MS: (*m/z*, %) = 216 (M^+^, 80), 159 (40), 103 (100); IR ν*_max_* (KBr)/cm^−1^ 2989, 2926, 1722, 1598, 1496, 1398, 1187, 852, 739; NMR δ*_H_* (400 MHz, DMSO-*d*_6_) 11.95 (br, 1H, NH), 8.12 (d, 1H, *J* = 8.4), 7.83 (d, 1H, *J =* 8.4), 7.64 (t, 1H, *J* = 8.6), 7.48 (t, 1H, *J =* 8.6), 6.35 (dd, 1H, *J =* 9.6, 5.2), 3.43 (dd, 1H, *J =* 18.4, 9.2), 3.32 (dd, 1H, *J =* 18.4, 9.2); δ*_C_* (150 MHz, DMSO-*d*_6_) 175.8, 174.8, 145.6, 133.4, 128.6, 125.0, 120.0, 110.8, 57.3, 36.7; HR-MS (EI)* m/z* [M]^+^ calcd for C_10_H_8_N_4_O_2_ 216.0647, found 216.0641.

*3-(2H-Benzotriazol-2-yl)-1-phenylpyrrolidine-2,5-dione* (**7a**). A white solid (76 mg) from column chromatography using ethyl acetate/pet. ether (1:2, *R_f_* = 0.54), mp. 218–219 °C; MS: (*m/z*, %) = 292 (M^+^, 80), 173 (80), 145 (100); IR ν*_max_* (KBr)/cm^−1^ 2990, 2966, 2852, 1722, 1595, 1499, 1395, 1216, 1184, 840, 748; NMR δ*_H_* (400 MHz, CDCl_3_) 7.91–7.88 (m, 2H), 7.53 (dt, 2H, *J =* 8.2, 1.6), 7.49–7.42 (m, 5H), 6.13 (dd, 1H, *J =* 9.6, 5.6), 3.66 (dd, 2H, *J =* 12.4, 9.2); δ*_C_* (100 MHz, CDCl_3_) 172.3, 170.2, 145.1, 131.4, 129.6, 129.4, 127.6, 126.6, 118.5, 63.1, 36.3; HR-MS (EI)* m/z* [M]^+^ calcd for C_16_H_12_N_4_O_2_ 292.0960, found 292.0954.

*3-(2H-Benzotriazol-2-yl)-1-methylpyrrolidine-2,5-dione* (**7b**). A white solid (74 mg) from column chromatography using ethyl acetate/pet. ether (1:1, *R_f_* = 0.58), mp. 155–157 °C; MS: (*m/z*, %) = 230 (M^+^, 60), 145 (100), 91 (15); IR ν*_max_* (KBr)/cm^−1^ 2980, 2916, 2854, 1720, 1585, 1479, 1395, 1216, 840, 768; NMR δ*_H_* (400 MHz, CDCl_3_) 7.88–7.85 (m, 2H), 7.44–7.41 (m, 2H), 5.98 (dd, 1H, *J* = 9.2, 5.6), 3.54 (dd, 1H, *J =* 18.4, 5.6), 3.44 (dd, 1H, *J =* 18.4, 5.6), 3.17 (s, 3H, CH_3_); δ*_C_* (150 MHz, CDCl_3_) 173.2, 171.3, 145.1, 127.6, 118.5, 63.1, 36.2, 25.9; HR-MS (EI)* m/z* [M]^+^ calcd for C_11_H_10_N_4_O_2_ 230.0804, found 230.0799.

*3-(2H-Benzotriazol-2-yl)-pyrrolidine-2,5-dione* (**7c**). A white solid (65 mg) from column chromatography using ethyl acetate/pet. ether (1:1, *R_f_* = 0.58), mp. 168–170 °C; MS (*m/z*, %) = 216 (M^+^, 85), 145 (30), 103 (100); IR ν*_max_* (KBr)/cm^−1^ 3236, 2959, 2930, 2861, 1795, 1727, 1595, 1462, 1276, 1125, 1072, 744; NMR δ*_H_* (600 MHz, CDCl_3_) 8.14 (br, 1H, NH), 7.87 (dd, 2H, *J =* 7.2, 3.0), 7.46 (dd, 2H, *J =* 6.6, 3.0), 6.02 (dd, 1H, *J* = 11.4, 6.0), 3.61 (dd, 1H, *J =* 18.6, 5.6), 3.50 (dd, 1H, *J =* 18.6, 5.6); δ*_C_* (150 MHz, CDCl_3_) 172.2, 170.5, 145.1, 127.7, 118.5, 64.0, 37.2; HR-MS (EI)* m/z* [M]^+^ calcd for C_10_H_8_N_4_O_2_ 216.0647, found 216.0641.

### 3.4. Photoproducts from Irradiation of Benzotriazoles **1a**,**b** with Alkynes **8a**–**e**

*2-Phenyl-1H-indole* (**9a**). White solid (120 mg), mp. 189–190 °C (187–188 °C, [26]); MS: (*m/z*,%) = 193 (M^+^, 100), 165 (25), 96 (15); NMR δ*_H_* (400 MHz, CDCl_3_) 8.35 (br, 1H, NH), 7.66 (d, 2H, *J =* 7.6), 7.63 (d, 1H, *J =* 7.6), 7.46–77.40 (m, 2H), 7.35 (t, 1H, *J*= 8.0), 7.20 (dt, 1H, *J =* 8.0, 1.2), 7.17 (dt, 1H, *J =* 8.0, 1.2), 7.13 (t, 1H, *J =* 8.0), 6.84 (s, 1H); δ*_C_* (100 MHz, CDCl_3_) 138.1, 137.0, 132.6, 129.5, 129.3, 127.9, 125.4, 122.6, 120.9, 120.5, 111.1, 100.2; HR-MS (EI)* m/z* [M]^+^ calcd for C_14_H_11_N 193.0891, found 193.0886.

*2-Cyclohexen-1-yl-1H-indole* (**9b**). White solid (142 mg), mp. 138–140 °C (137–139 °C, [26]); MS: (*m/z*, %) = 197 (M^+^, 100), 168 (65); NMR δ*_H_* (600 MHz, CDCl_3_) 8.09 (br, 1H, NH), 7.55 (d, 1H, *J =* 7.8), 7.30 (d, 1H, *J =* 7.8), 7.14 (t, 1H, *J =* 7.4), 7.06 (t, 1H, *J =* 7.4), 6.44 (s, 1H), 6.14 (s, 1H), 2.47 (br, 2H), 2.26–2.25 (br, 2H), 1.82–1.78 (m, 2H), 1.72–1.69 (m, 2H); δ*_C_* (150 MHz, CDCl_3_) 139.7, 136.4, 129.4, 129.2, 122.8, 122.2, 120.6, 120.0, 110.6, 98.9, 26.3, 25.8, 22.8, 22.4; HR-MS (EI)* m/z* [M]^+^ calcd for C_14_H_15_N 197.1204, found 197.1199.

*Ethyl 1H-indole-3-carboxylate* (**9c**). White solid (142 mg), mp. 124–126 °C (125–127 °C, [24,26]); MS: (*m/z*, %) = 189 (M^+^, 50), 144 (100); NMR δ*_H_* (400 MHz, CDCl_3_): 8.73 (br, 1H, NH), 8.23 (m, 1H), 7.94 (d, 1H, *J =* 2.8), 7.45–7.41 (m, 1H), 7.32–7.27 (m, 2H), 4.42 (q, 2H, *J =* 7.4), 1.45 (t, 3H, *J =* 7.4); δ*_C_* (150 MHz, CDCl_3_) 165.6, 136.3, 131.2, 126.0, 123.4, 122.2, 121.8, 111.7, 109.3, 60.1, 14.8; HR-MS (EI)* m/z* [M]^+^ calcd for C_11_H_11_NO_2_ 189.0790, found 189.0784.

*Dimethyl 1-methyl-1H-indole-2,3-dicarboxylate* (**9d**). Yellow solid (122 mg), mp. 38–40 °C (37–40 °C, [27]); MS: (*m/z*, %) = 247 (M^+^, 60), 216 (100), 149 (50); NMR δ*_H_* (400 MHz, CDCl_3_): 8.12 (d, 1H, *J =* 8.0), 7.38–7.36 (m, 2H), 7.32–7.26 (m, 1H), 4.02 (s, 3H, CH_3_), 3.93 (s, 3H, CH_3_), 3.84 (s, 3H, CH_3_); δ*_C_* (100 MHz, CDCl_3_): 164.8, 163.5, 137.0, 134.9, 129.7, 125.5, 124.7, 122.9, 122.6, 110.4, 53.3, 51.7, 31.6; HR-MS (EI)* m/z* [M]^+^ calcd for C_13_H_13_NO_4_ 247.0845, found 247.0839.

*Diethyl 1-methyl-1H-indole-2,3-dicarboxylate* (**9e**). A yellow oil (126 mg) from column chromatography using ethyl acetate/pet. ether (1:1, *R_f_* = 0.58), MS: (*m/z*, %) = 275 (M^+^ , 80), 230 (35), 202 (95); NMR δ*_H_* (400 MHz, CDCl_3_) 8.14 (d, 1H, *J =* 7.6), 7.38–7.36 (m, 2H), 7.32–7.28 (m, 1H), 4.48 (q, 2H, *J =* 7.2), 4.38 (q, 2H, *J =* 7.2), 3.84 (s, 3H), 1.44 (t, 3H, *J =* 7.2), 1.41 (t, 3H, *J =* 7.2); δ*_C_* (100 MHz, CDCl_3_) 164.3, 163.1, 136.9, 135.2, 125.6, 124.5, 122.7, 122.5, 110.3, 108.1, 62.5, 60.4, 31.6, 14.6, 14.3; HR-MS (EI)* m/z* [M]^+^ calcd for C_15_H_17_NO_4_ 275.1158, found 275.1153 [28].

*Ethyl N-methylindole-2-carboxylate* (**9f**). A white solid (80 mg) from column chromatography using ethyl acetate/pet. ether (1:3, *R_f_* = 0.68), mp. 58–60 °C (59–60 °C, [29]); MS: (*m/z*, %) = 203 (M^+^, 40), 178 (70), 89 (85); NMR δ*_H_* (400 MHz, CDCl_3_) 7.71 (d, 1H, *J =* 7.6), 7.40–7.33 (m, 2H), 7.31 (s, 1H), 7.15 (dt, 1H, *J =* 7.0, 1.2), 4.38 (q, 2H, *J =* 7.2), 4.09 (s, 3H), 1.42 (t, 3H, *J* = 7.2); δ*_C_* (100 MHz, CDCl_3_) 162.3, 139.6, 128.8, 125.9, 124.9, 122.5, 120.5, 110.21, 110.07, 60.5, 31.6, 14.4; HR-MS (EI)* m/z* [M]^+^ calcd for C_12_H_13_NO_2_ 203.0946, found 203.0941.

*Dimethyl 2-(1H-benzotriazol-1-yl)maleate* (**10a**). A white solid (130 mg) from column chromatography using ethyl acetate/pet. ether (1:3, *R_f_* = 0.68), mp. 84–86 °C; MS: (*m/z*, %) = 261 (M^+^, 100), 202 (50), 175 (65); IR ν*_max_* (KBr)/cm^−1^ 3040, 2954, 1746, 1718, 1638, 1434, 1360, 1263, 1159, 1056, 746; NMR δ*_H_* (400 MHz, CDCl_3_) 8.16 (d, 1H, *J =* 8.4), 7.634–7.62 (m, 2H), 7.52–7.48 (m, 1H), 6.68 (s, 1H), 4.07 (s, 3H), 3.86 (s, 3H); δ*_C_* (100 MHz, CDCl_3_) 164.9, 162.5, 146.9, 140.6, 131.4, 130.0, 125.8, 121.4, 111.1, 110.1, 54.0, 52.8; HR-MS (EI)* m/z* [M]^+^ calcd for C_12_H_11_N_3_O_4 _261.0750, found 261.0745.

*Diethyl 2-(1H-benzotriazol-1-yl)maleate* (**10b**). A colorless oil (180 mg) from column chromatography using ethyl acetate/pet. ether (1:3 *R_f_* = 0.58); MS: (*m/z*, %) = 289 (M^+^, 40), 187 (90), 159 (100); ν*_max_* (KBr)/cm^−1^ 3040, 2954, 1746, 1718, 1638, 1434, 1360, 1263, 1159, 1056, 746; NMR δ*_H_* (600 MHz, CDCl_3_) 8.16 (dd, 1H, *J =* 7.8, 1.6), 7.67–7.62 (m, 2H), 7.60–7.48 (dd, 1H, *J =* 8.4, 1.2), 6.66 (s, 1H), 4.53 (q, 2H, *J =* 7.2), 4.32 (q, 2H, *J =* 7.2), 1.39 (t, 3H, *J =* 7.2) 1.35 (t, 3H, *J =* 7.2); δ*_C_* (100 MHz, CDCl_3_) 164.3, 162.0, 146.9, 140.5, 131.5, 129.8, 125.7, 121.3, 111.1, 110.8, 63.4, 61.8, 14.35, 14.05; HR-MS (EI)* m/z* [M]^+^ calcd for C_14_H_15_N_3_O_4_ 289.1063, found 289.1058.

*Dimethyl 2,3-Di(1H-benzotriazol-1-yl)succinate* (**11a**). A white solid (220 mg) from column chromatography using ethyl acetate/pet. ether (1:2, *R_f_* = 0.72), mp.147–149 °C; MS: (*m/z*, %) = 380 (M^+^, 15), 293 (40), 190 (95); IR ν*_max_* (KBr)/cm^−1^ 3051, 2991, 1759, 1730, 1613, 1556, 1452, 1303, 1278, 1169, 1001, 778, 750; NMR δ*_H_* (400 MHz, CDCl_3_) 7.73 (d, 2H, *J =*8.4), 7.43 (t, 4H, *J =* 8.0), 7.25–7.23 (m, 2H), 6.63 (s, 2H), 3.85 (s, 6H, 2CH_3_); δ*_C_* (100 MHz, CDCl_3_) 166.5, 145.3, 133.1, 128.6, 124.6, 120.0, 109.3, 58.9, 53.9; HR-MS (EI)* m/z* [M]^+^ calcd for C_18_H_16_N_6_O_4_ 380.1233, found 380.1228.

*Diethyl 2,3-di(1H-benzotriazol-1-yl)succinate* (**11b**). A white solid (230 mg) from column chromatography using ethyl acetate/pet. ether (1:1, *R_f_* = 0.58), mp. 124–126 °C; MS: (*m/z*, %)= 408 (M^+^, 5), 289 (45), 187 (95); IR ν*_max_* (KBr)/cm^−1^ 3051, 2991, 1759, 1730, 1613, 1556, 1452, 1303, 1278, 1169, 1001, 778, 750; NMR δ*_H_* (400 MHz, CDCl_3_) 7.77 (d, 2H, *J =* 8.4), 7.43–7.36 (m, 4H), 7.23–7.19 (m, 2H), 6.60 (s, 2H), 4.37–4.26 (m, 4H, 2CH_2_), 1.23 (t, 6H, *J =* 7.2, 2CH_3_); δ*_C_* (100 MHz, CDCl_3_) 166.0, 145.3, 133.2, 128.5, 124.5, 120.0, 109.0, 63.4, 60.3, 14.0; HR-MS (EI)* m/z* [M]^+^ calcd for C_20_H_20_N_6_O_4_ 408.1546, found 408.1540.

*Dimethyl 2-(2H-benzotriazol-2-yl)maleate* (**12**). A white solid (100 mg) from column chromatography using ethyl acetate/pet. ether (1:2, *R_f_* = 0.59), mp. 147–149 °C; MS: (*m/z*, %) = 261 (M^+^, 45), 230 (30), 71 (65); IR ν*_max_* (KBr)/cm^−1^ 3010, 2957, 1748, 1714, 1643, 1437, 1357, 1259, 1201, 1155, 1053, 744; NMR: δ*_H_* (400 MHz, CDCl_3_) 7.87–7.84 (m*,* 2H), 7.45–7.26 (m, 2H), 7.13 (s, 1H), 4.11 (s, 3H), 3.85 (s, 3H); δ*_C_* (150 MHz, CDCl_3_) 164.5, 162.0, 145.7, 143.9, 128.9, 118.7, 110.7, 53.8, 52.6; HR-MS (EI)* m/z* [M]^+^ calcd for C_12_H_11_N_3_O_4_ 261.0750 found 261.0744.

## 4. Conclusions

For the first time intermolecular trapping of the diradical intermediates, formed by irradiation of benzotriazoles with a 16 W low pressure mercury arc-lamp (λ = 254 nm) in the presence of electron poor alkenes and alkynes has been achieved. The present study offers an interesting simple access to dihydropyrrolo[3,4-*b*]indoles and functionally substituted indoles of potential synthetic and biological interest.

## Supplementary Materials

Supplementary materials can be accessed at: http://www.mdpi.com/1420-3049/19/12/20695/s1.
