# Supplementary material for: Photochemistry of Benzotriazoles: Generation of 1,3-Diradicals and Intermolecular Cycloaddition as a New Route toward Indoles and Dihydropyrrolo[3,4-b]Indoles
Source: Molecules. 2014 Dec 11;19(12):20695–708. doi: 10.3390/molecules191220695 (PMC6271786; doi:10.3390/molecules191220695)
Supplement: Supplementary file 1 [file molecules-19-20695-s001.pdf]

Supplementary Materials

Figure S1. ^{1}H-NMR of **4a**.

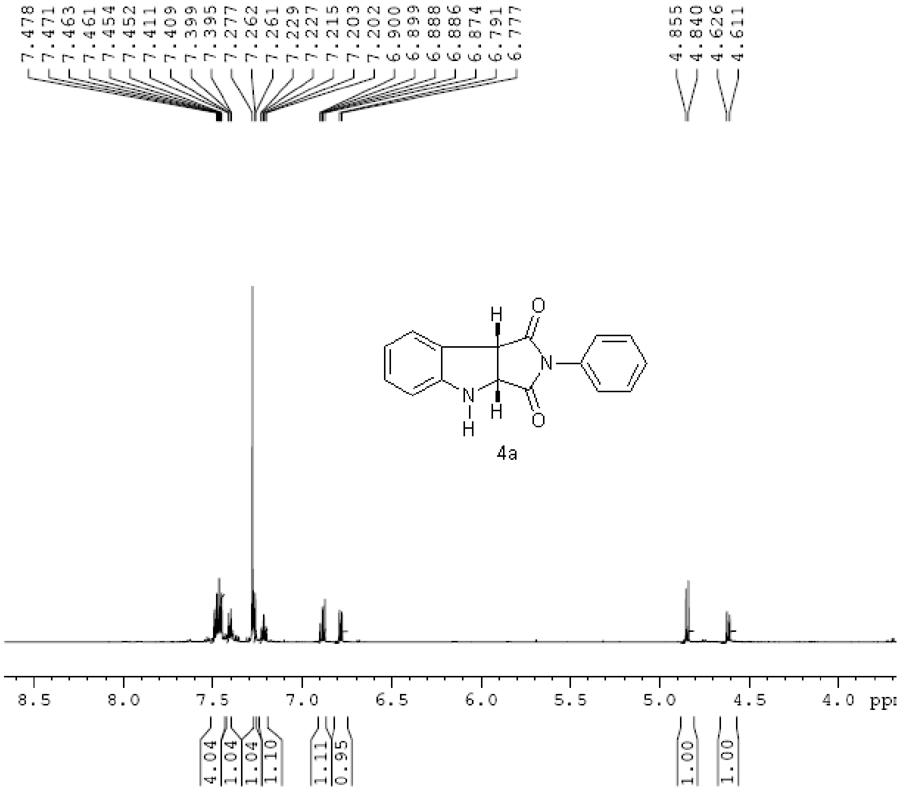

Figure S2. ^{13}C-NMR of **4a**.

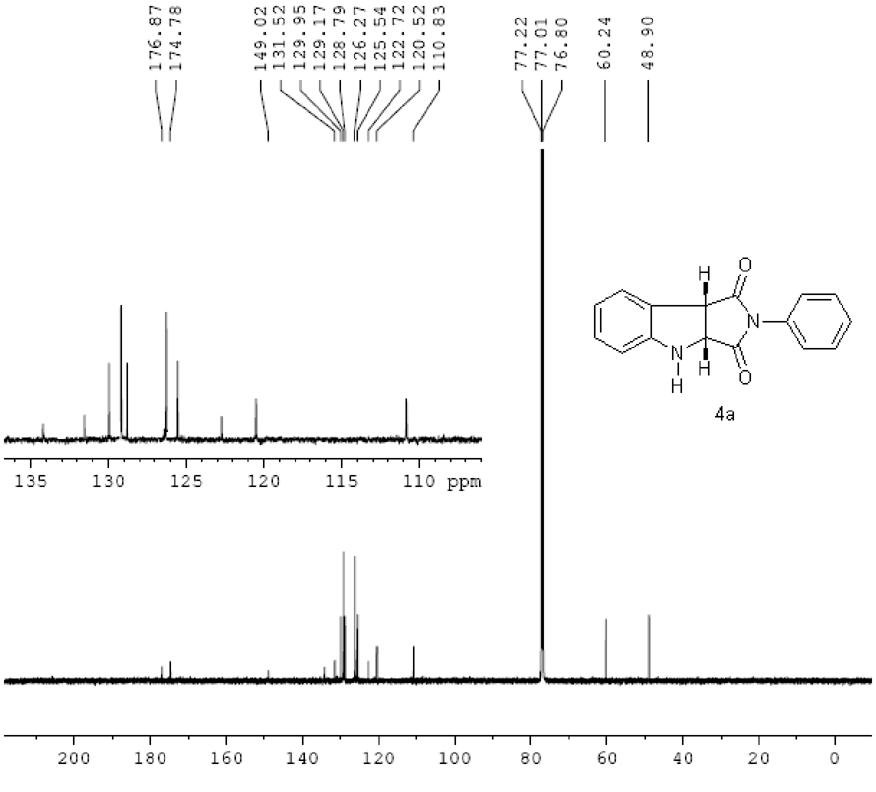

Figure S3. ^{1}H-NMR and ^{13}C-NMR of **4b**.

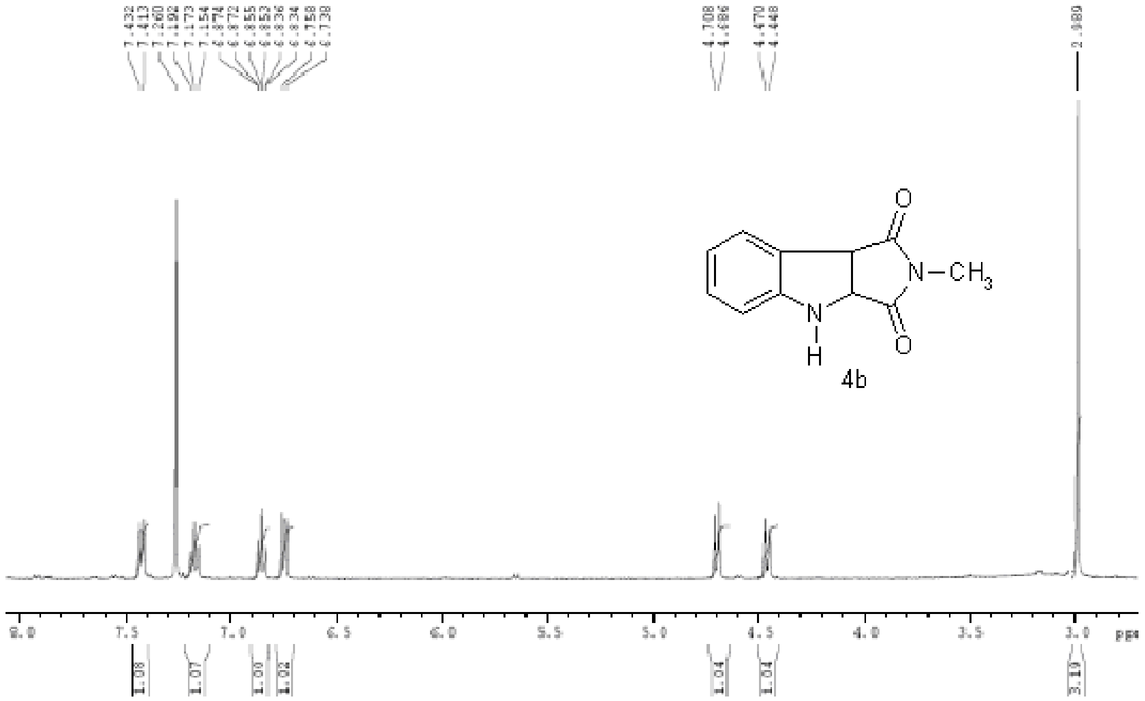

Figure S4. ^{13}C-NMR of **4b**.

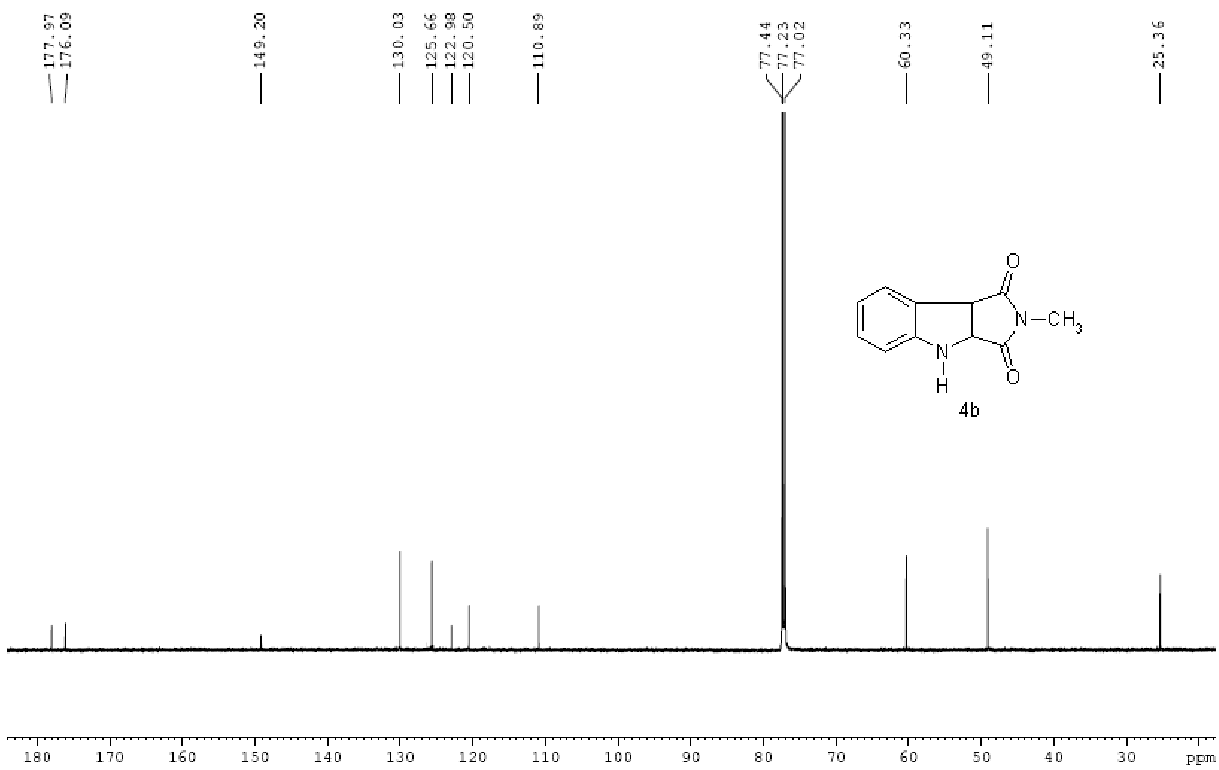

Figure S5. HSQC of **4b**.

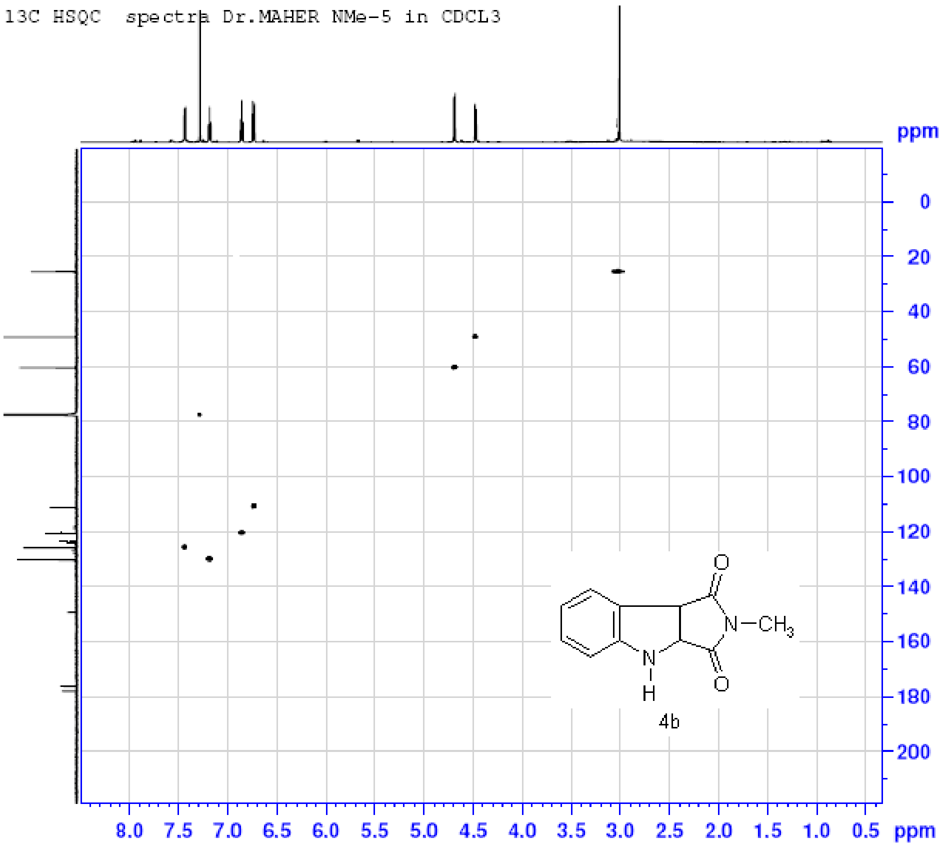

Figure S6. H,H COSY of **4b**.

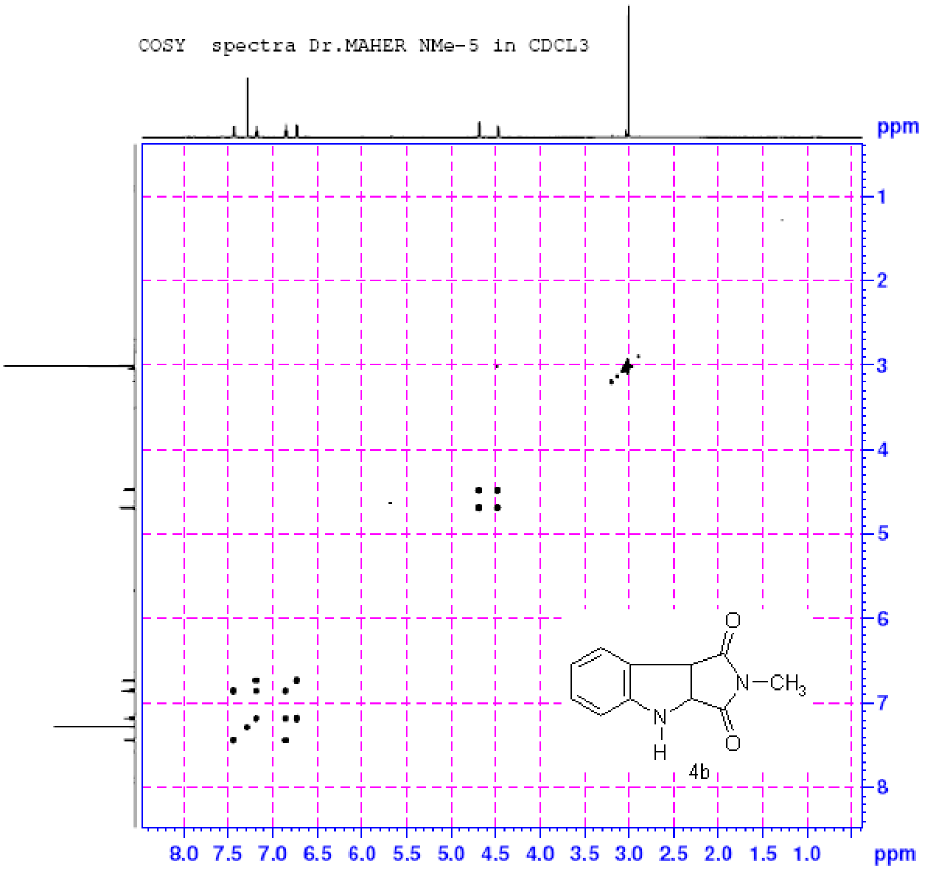

Figure S7. ^{1}H-NMR of **4c**.

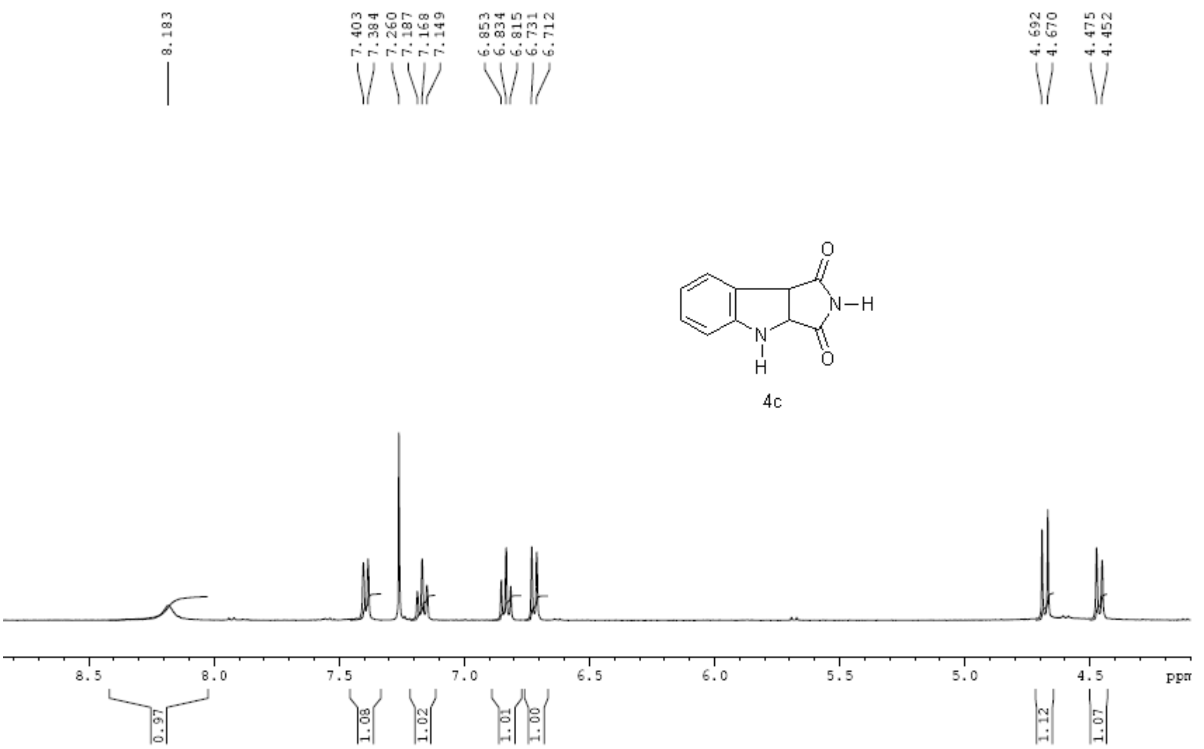

Figure S8. ^{13}C-NMR of **4c**.

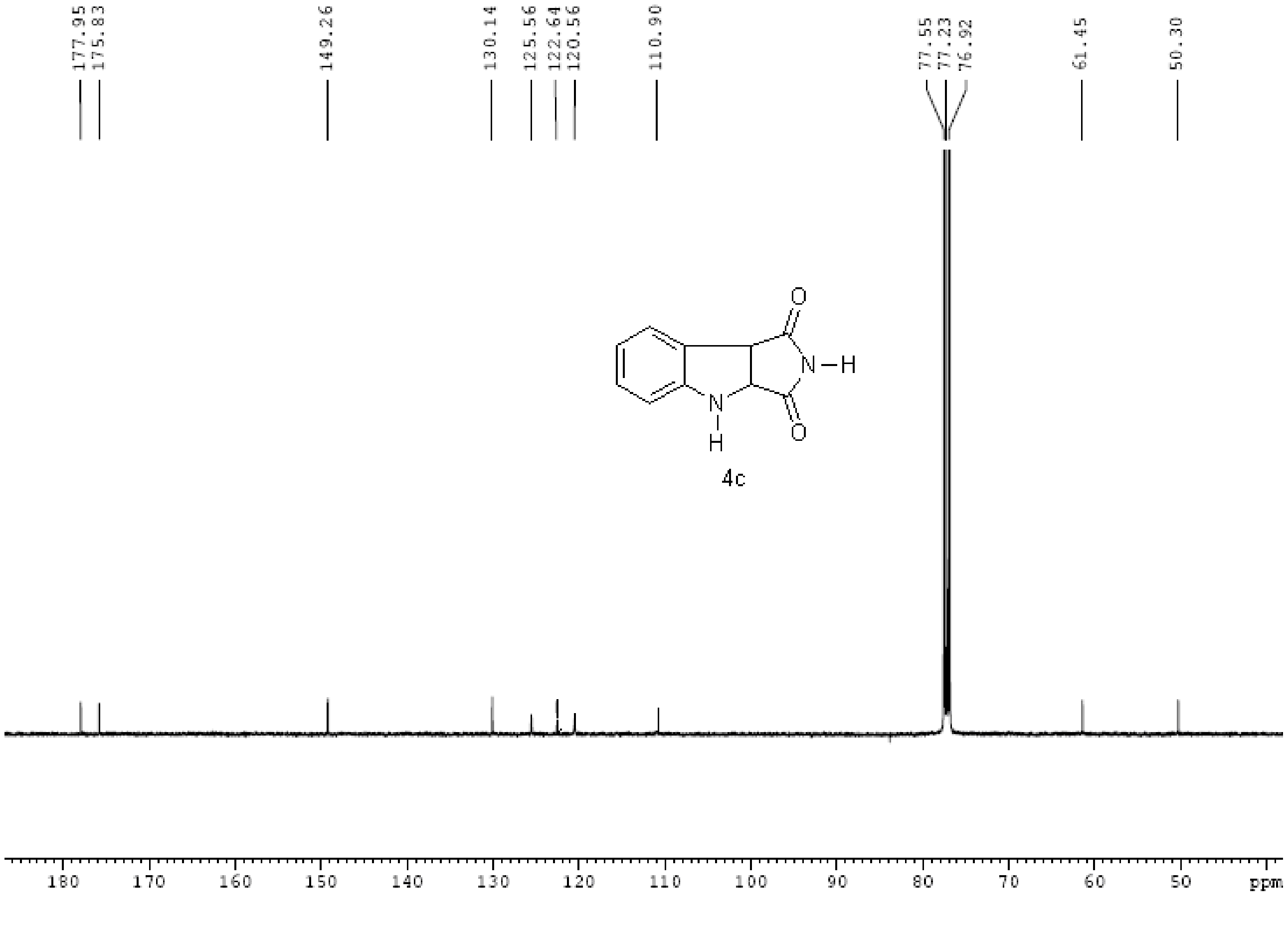

Figure S9. ^{1}H-NMR of **4**d.

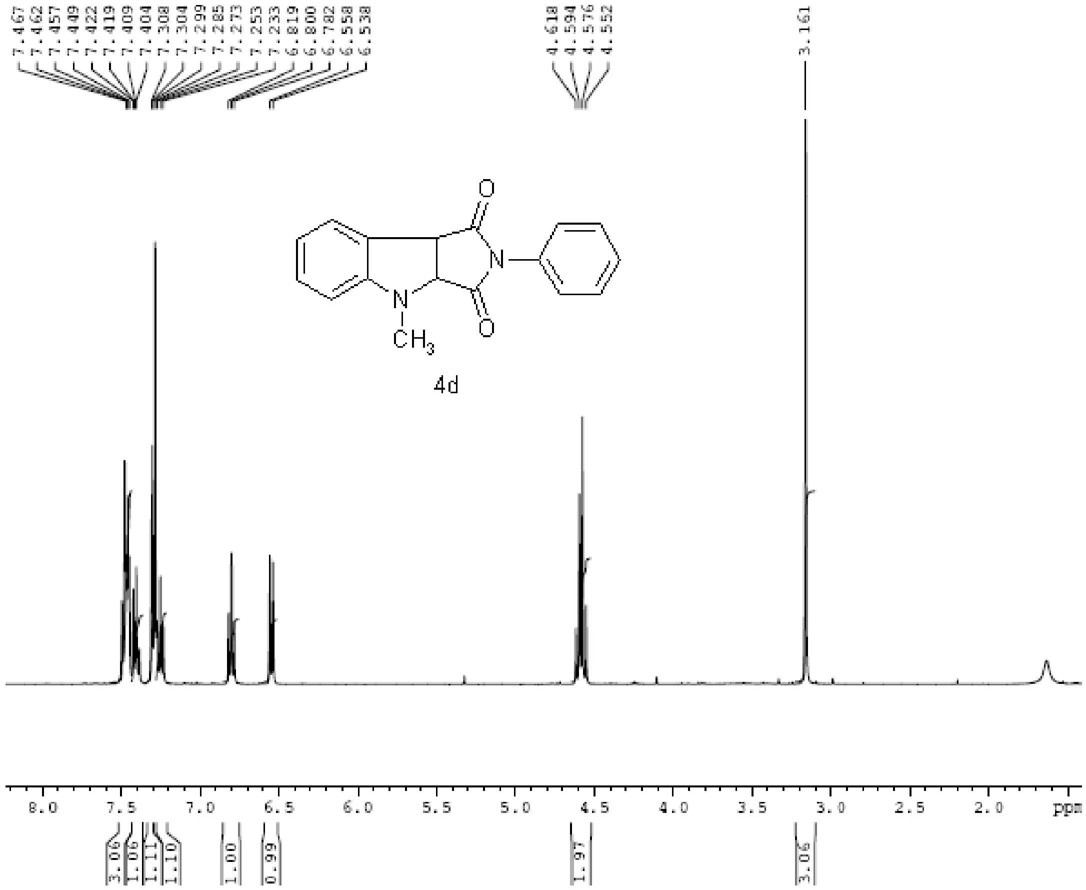

Figure S10. ^{13}C-NMR of **4d**.

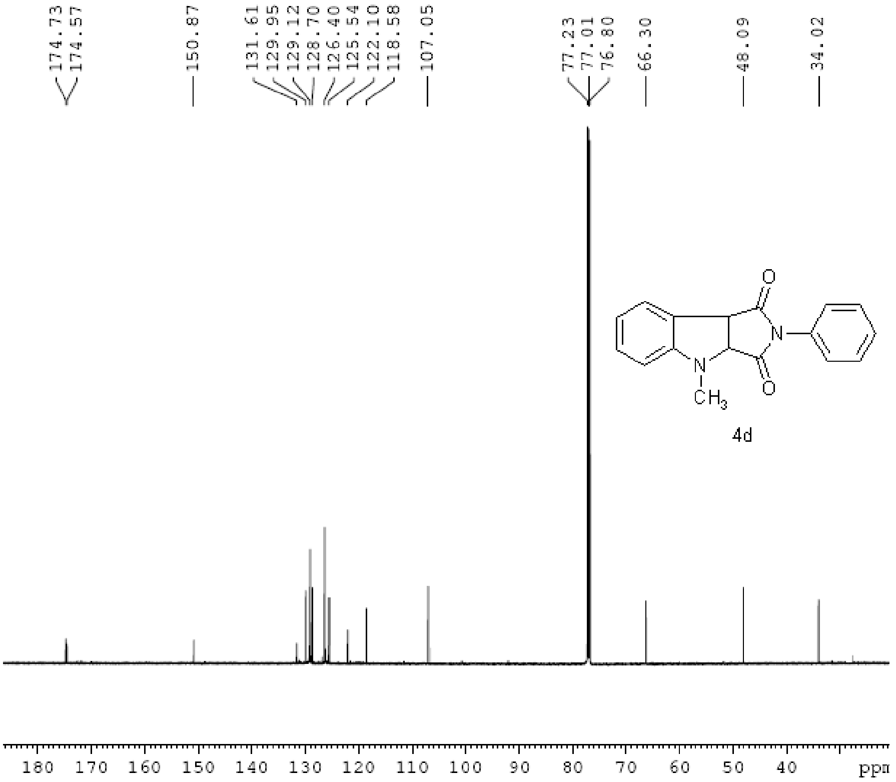

Figure S11. ^{1}H-NMR of **4e**.

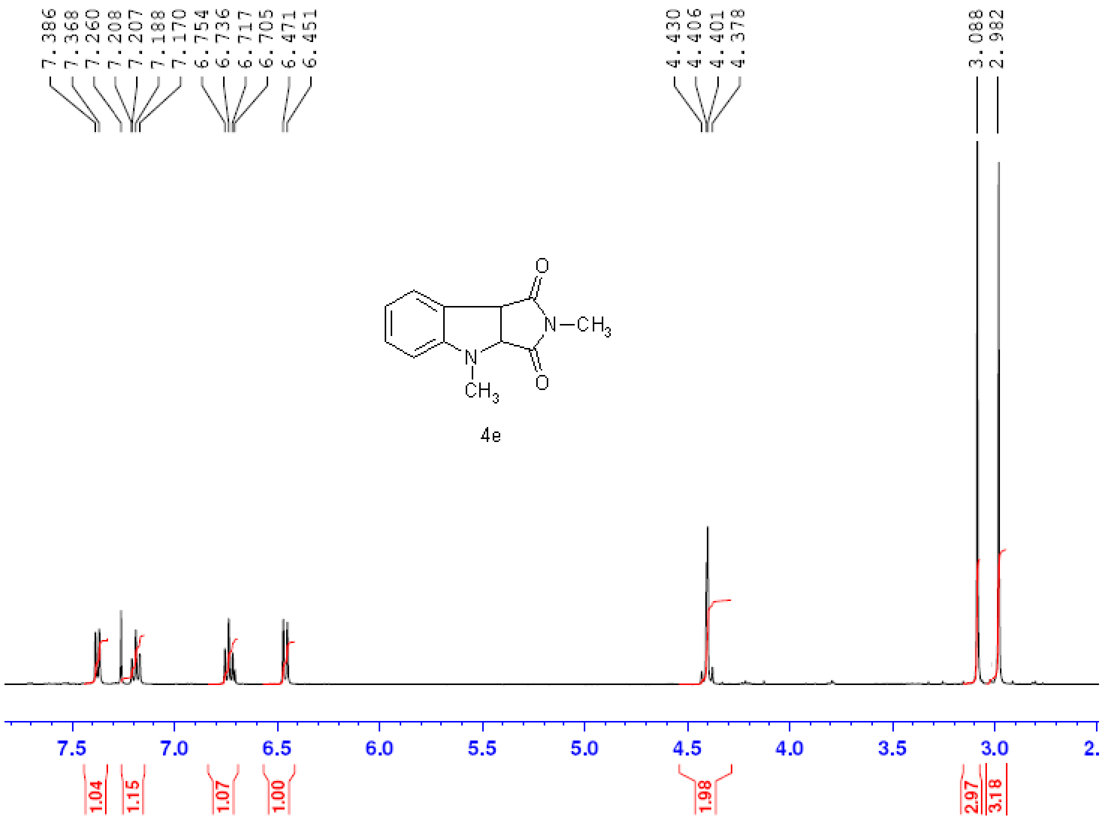

Figure S12. ^{13}C-NMR of **4e**.

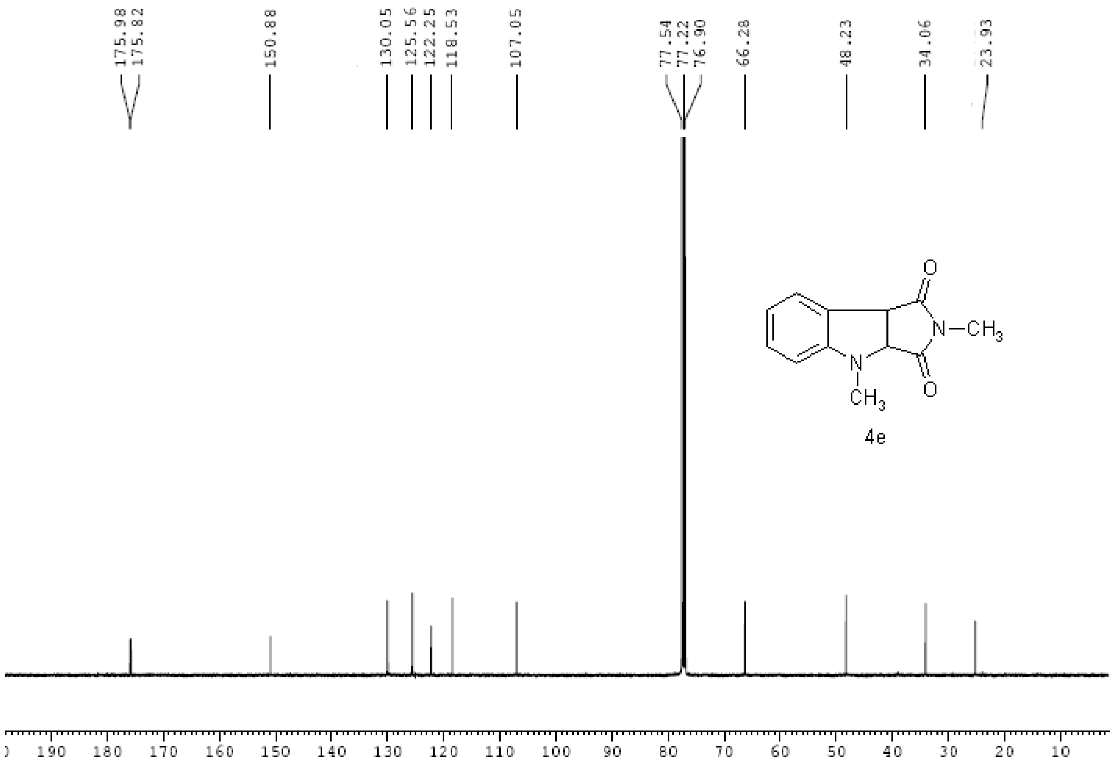

Figure S13. ^{1}H-NMR of **4f**.

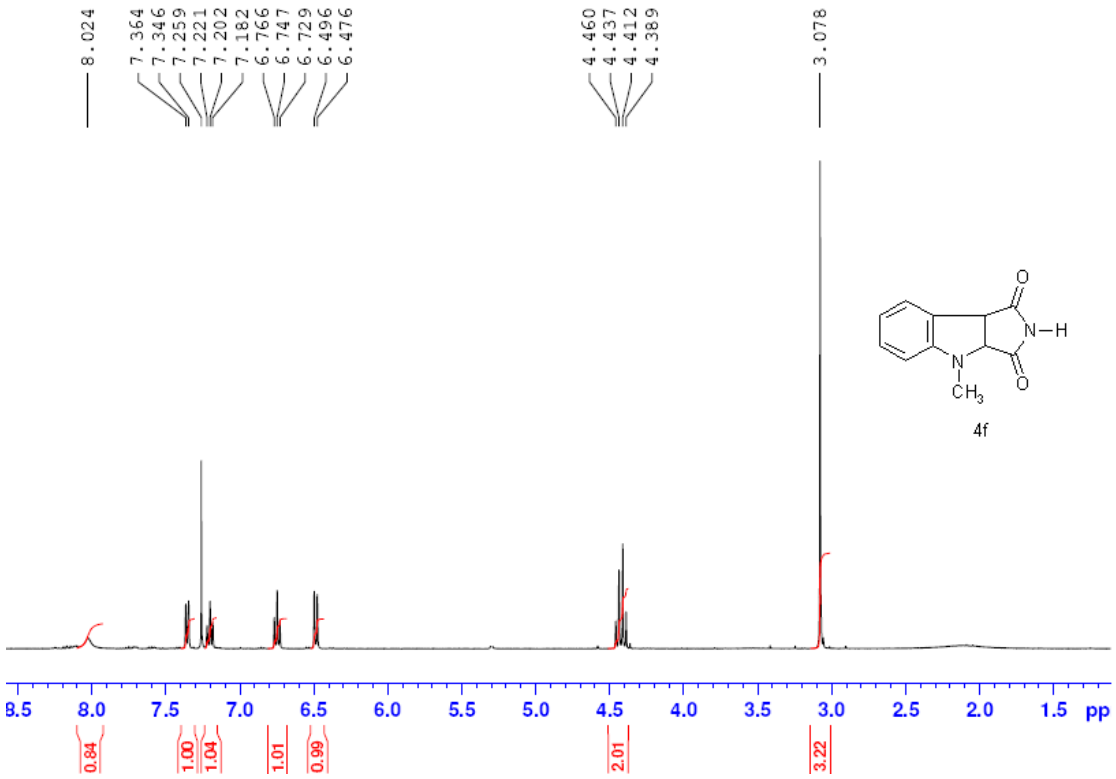

Figure S14. ^{13}C-NMR of **4f**.

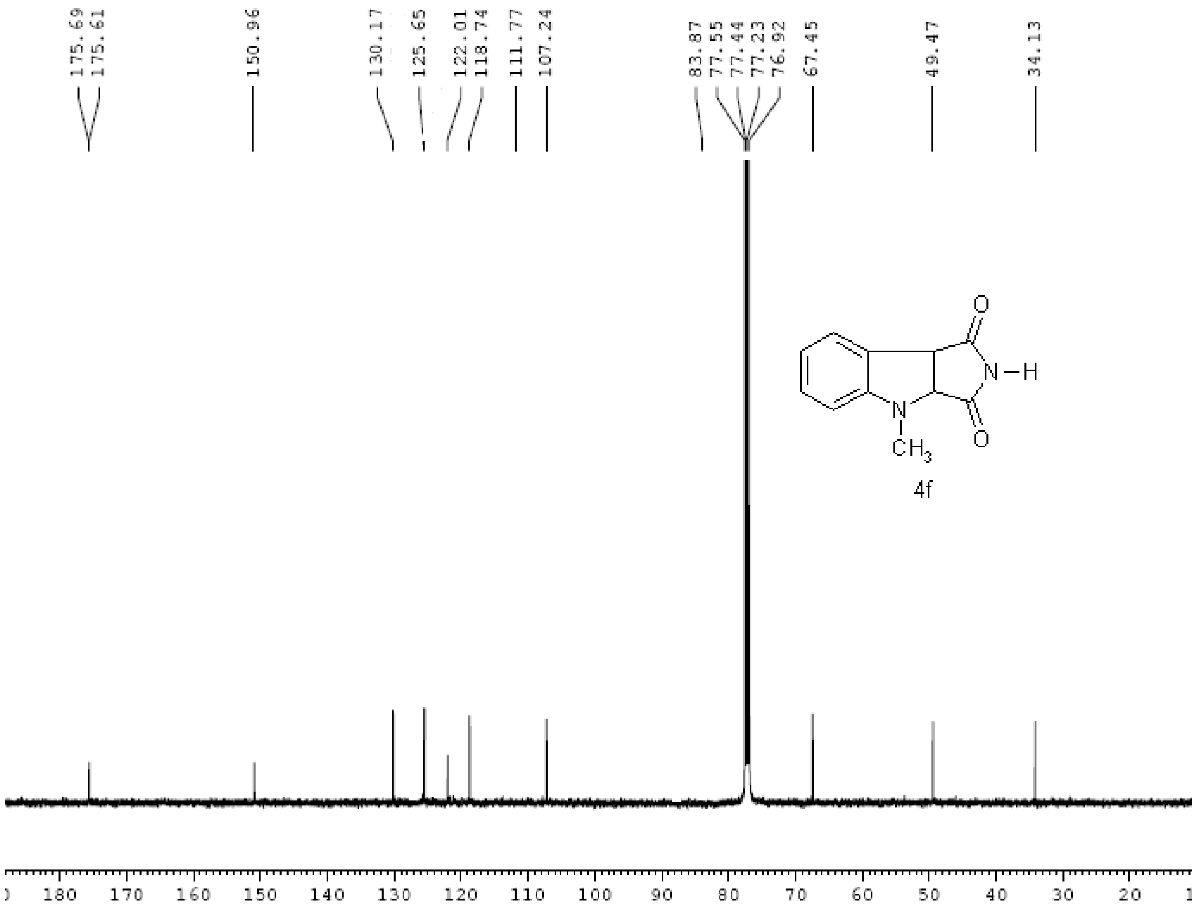

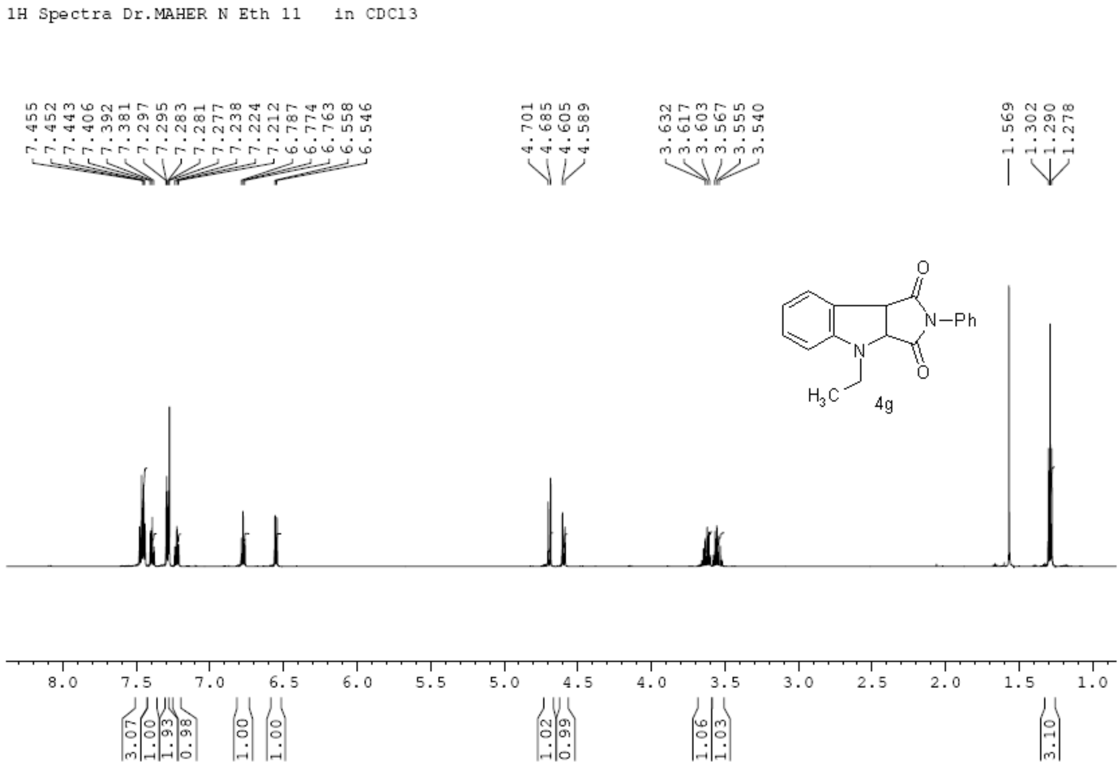

Figure S15. ^{1}H-NMR of **4g**.

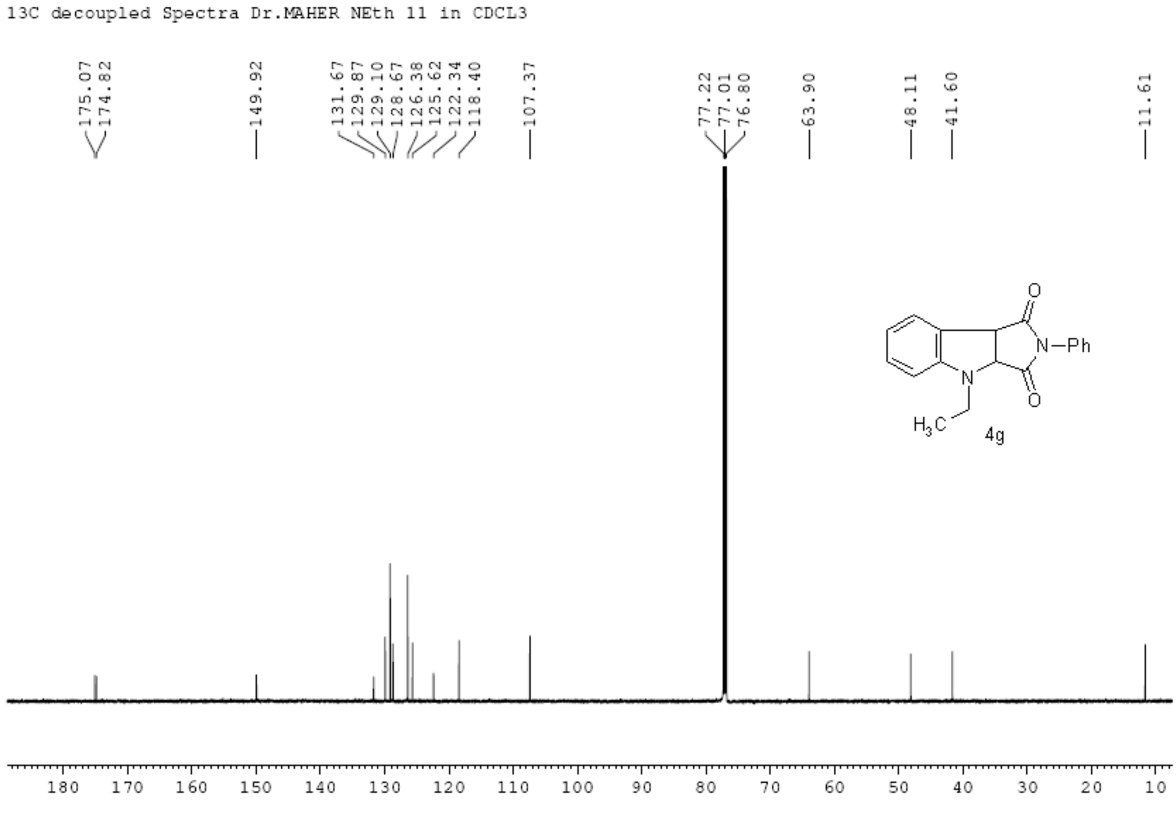

Figure S16. ^{13}C-NMR of **4g**.

Figure S17. ^{1}H-NMR of **4h**.

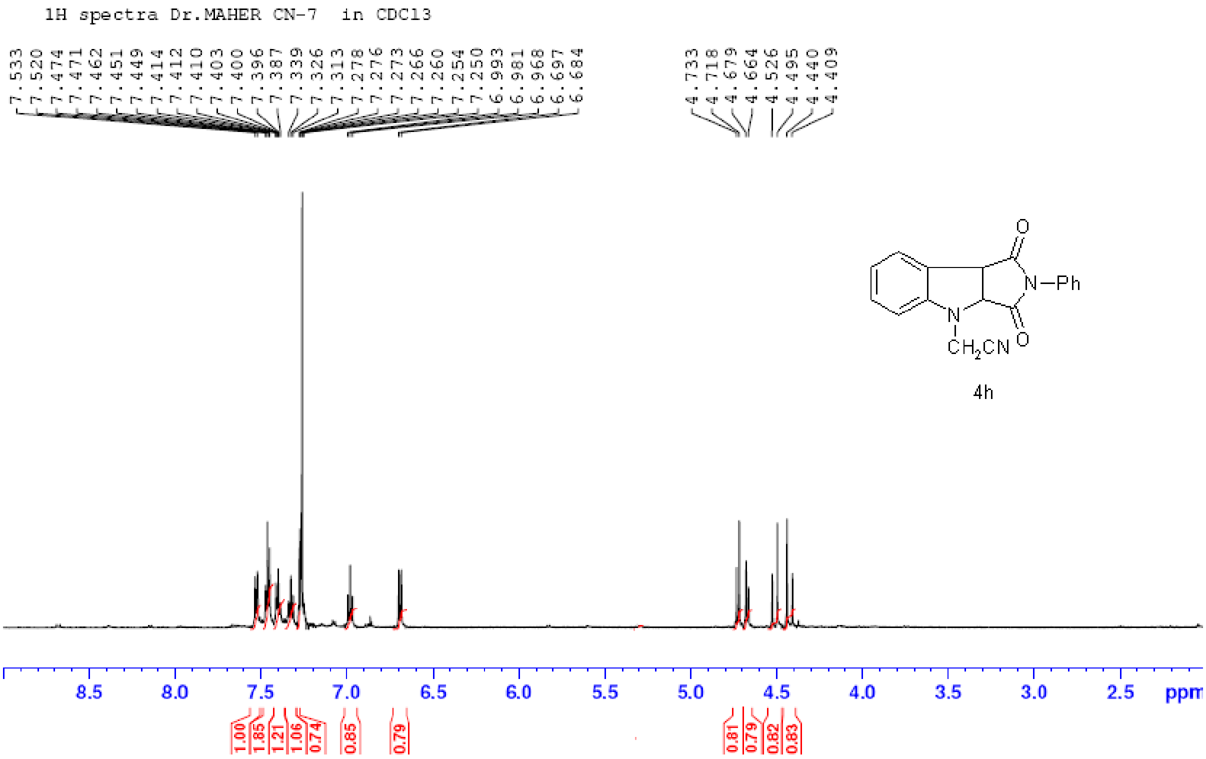

Figure S18. ^{1}H-NMR of **4i**.

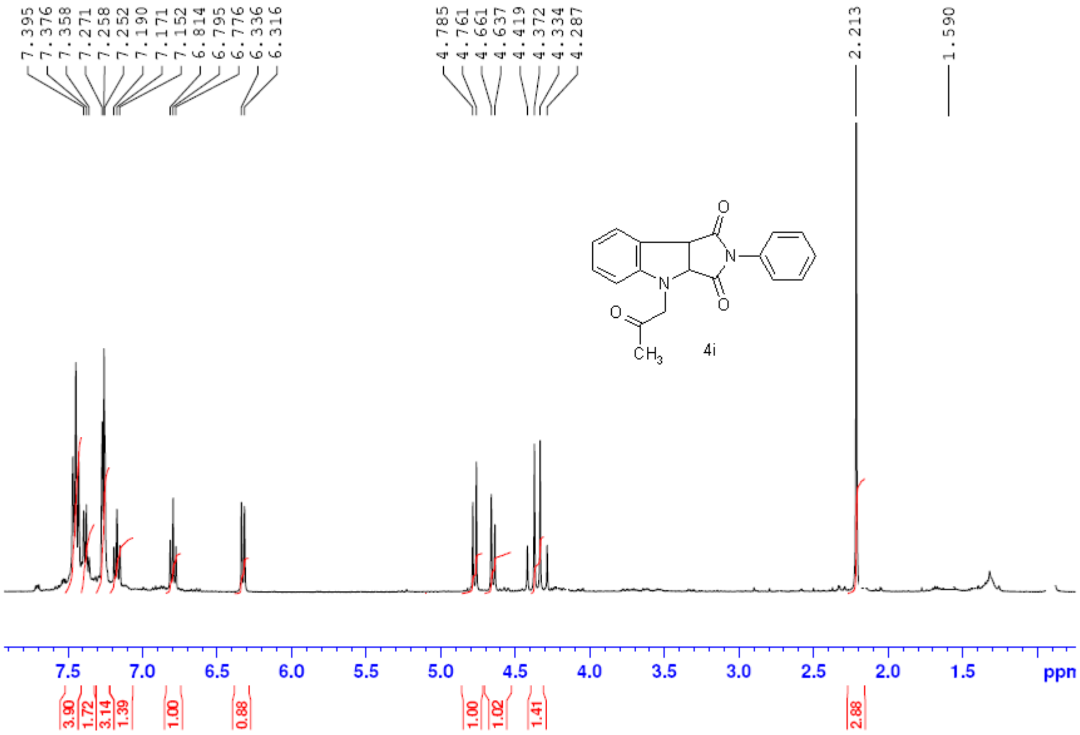

Figure S19. ^{13}C-NMR of **4i**.

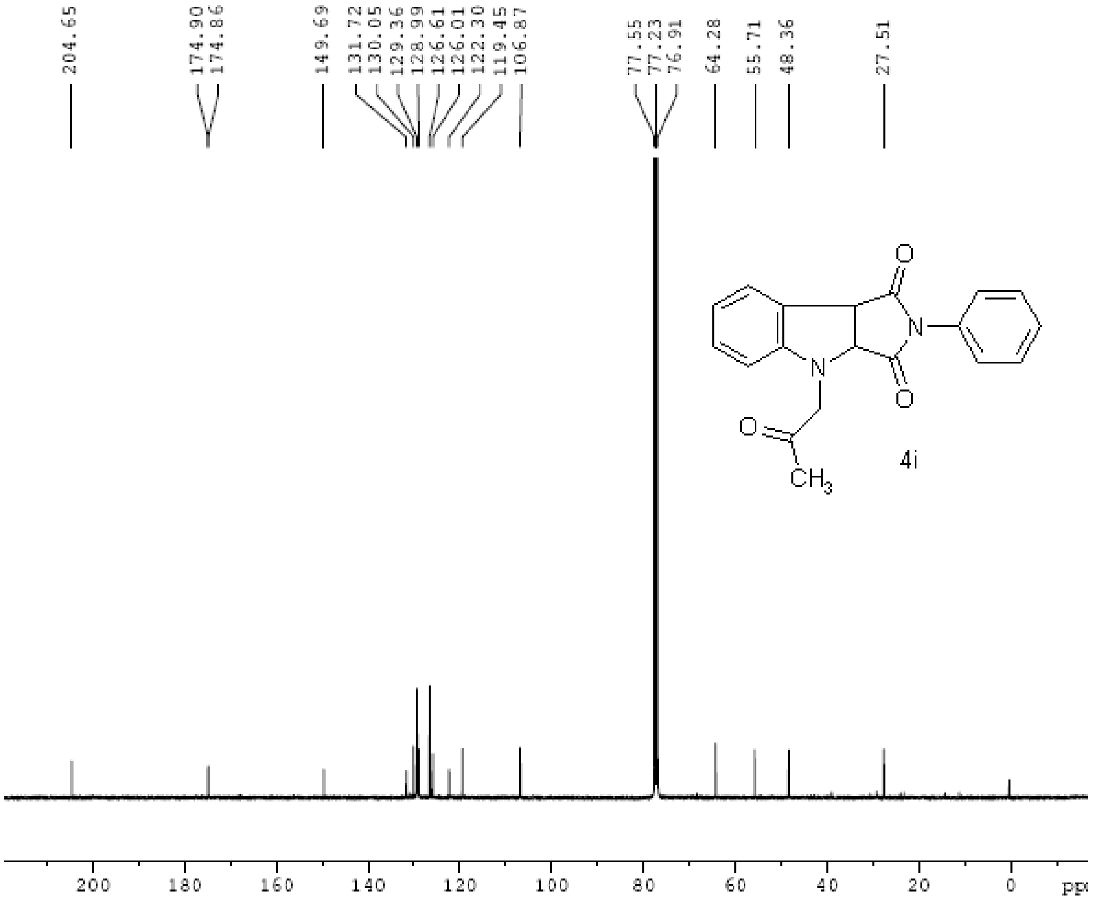

Figure S20. ^{1}H-NMR of **5**.

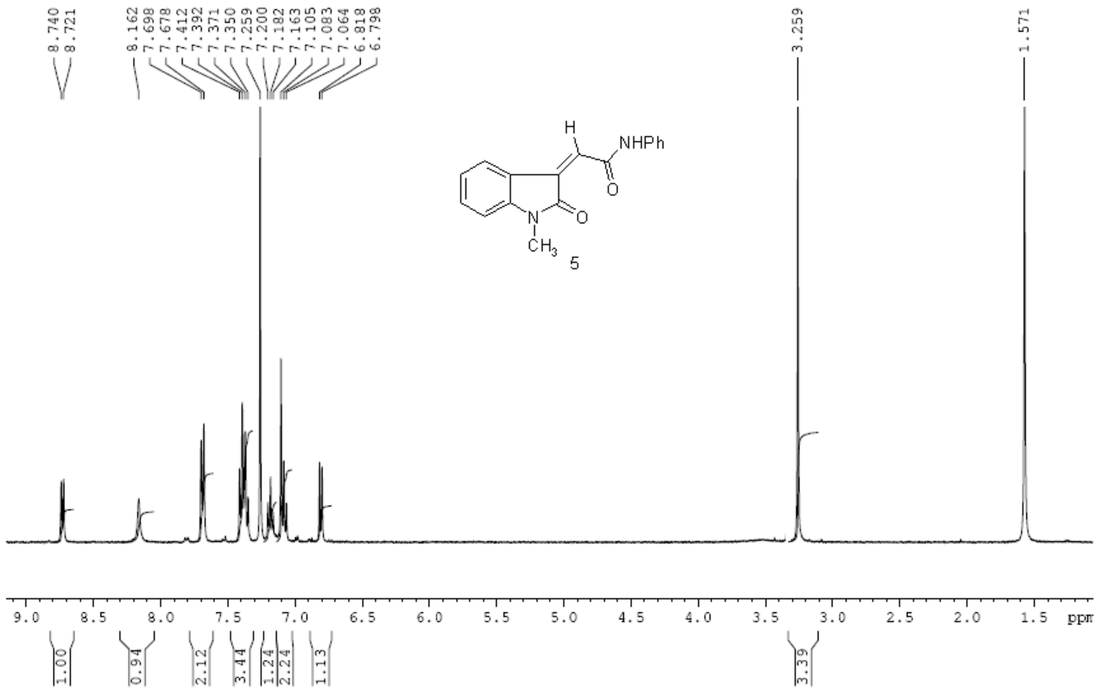

Figure S21. ^{13}C-NMR of **5**.

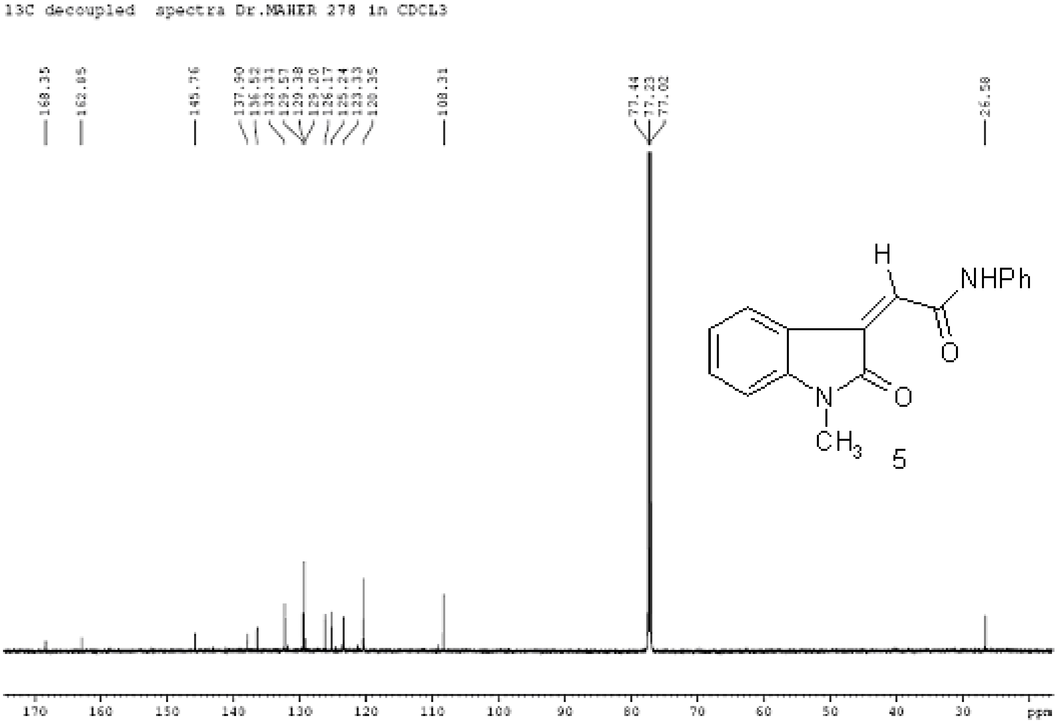

Figure S22. ^{13}C-NMR of **6b**.

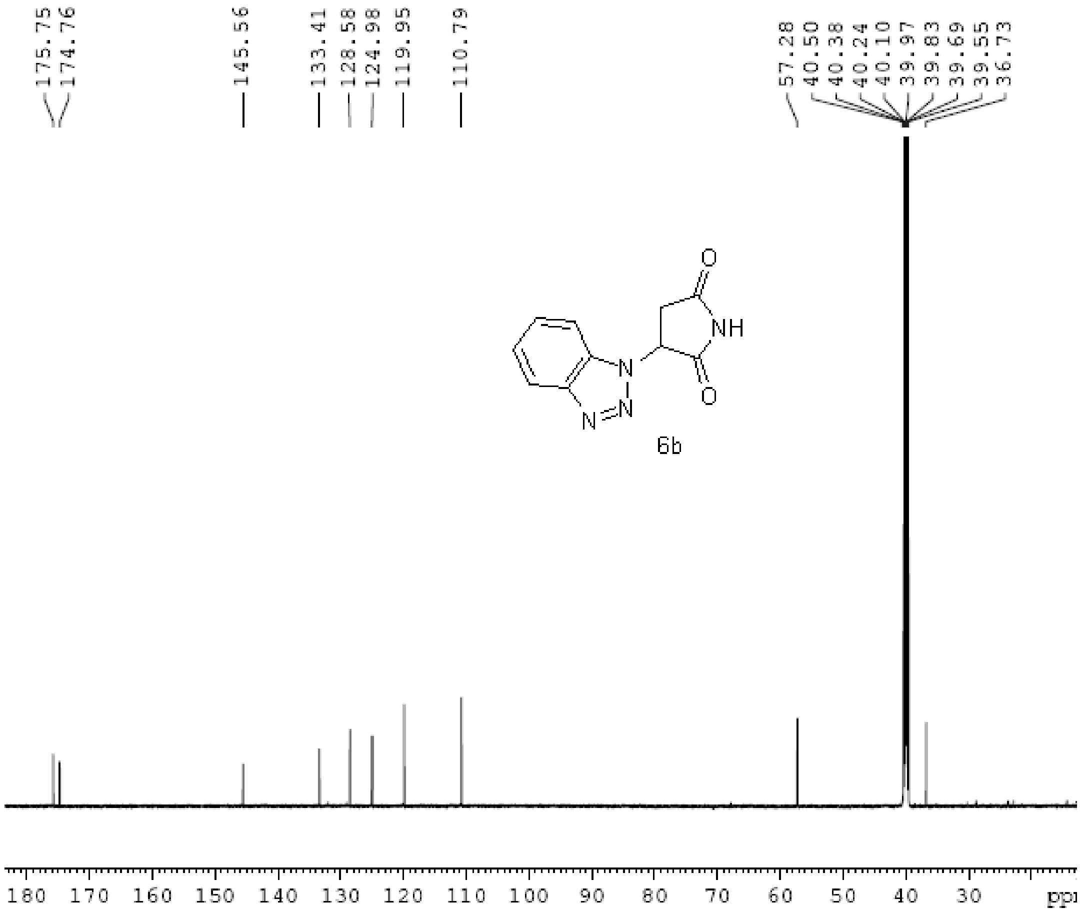

Figure S23. ^{1}H-NMR of **7a**.

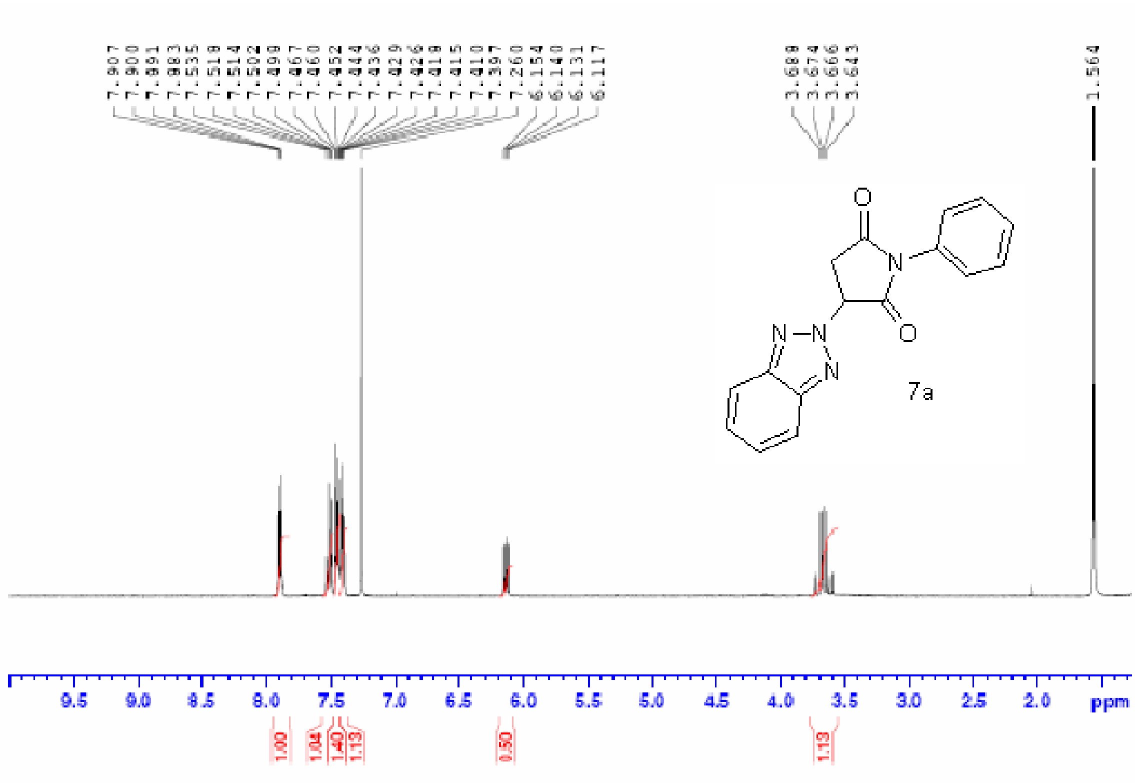

Figure S24. ^{13}C-NMR of **7a**.

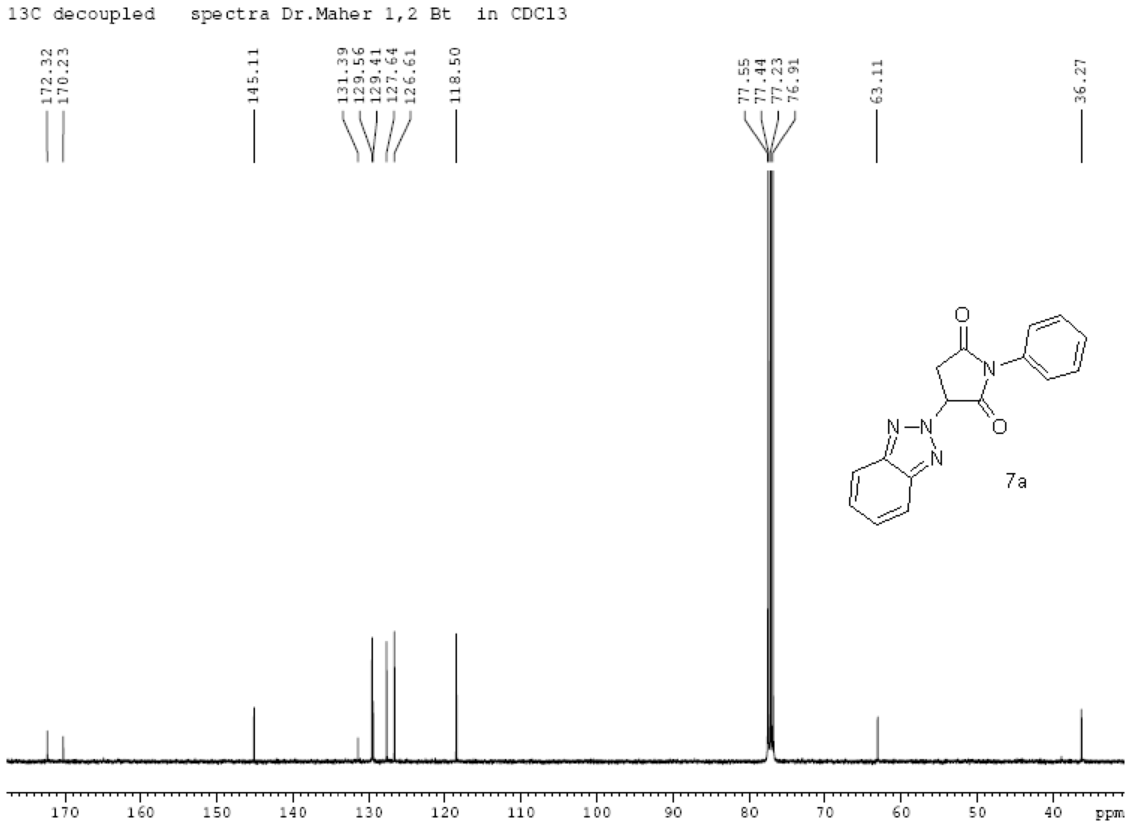

Figure S25. ^{1}H-NMR of **7b**.

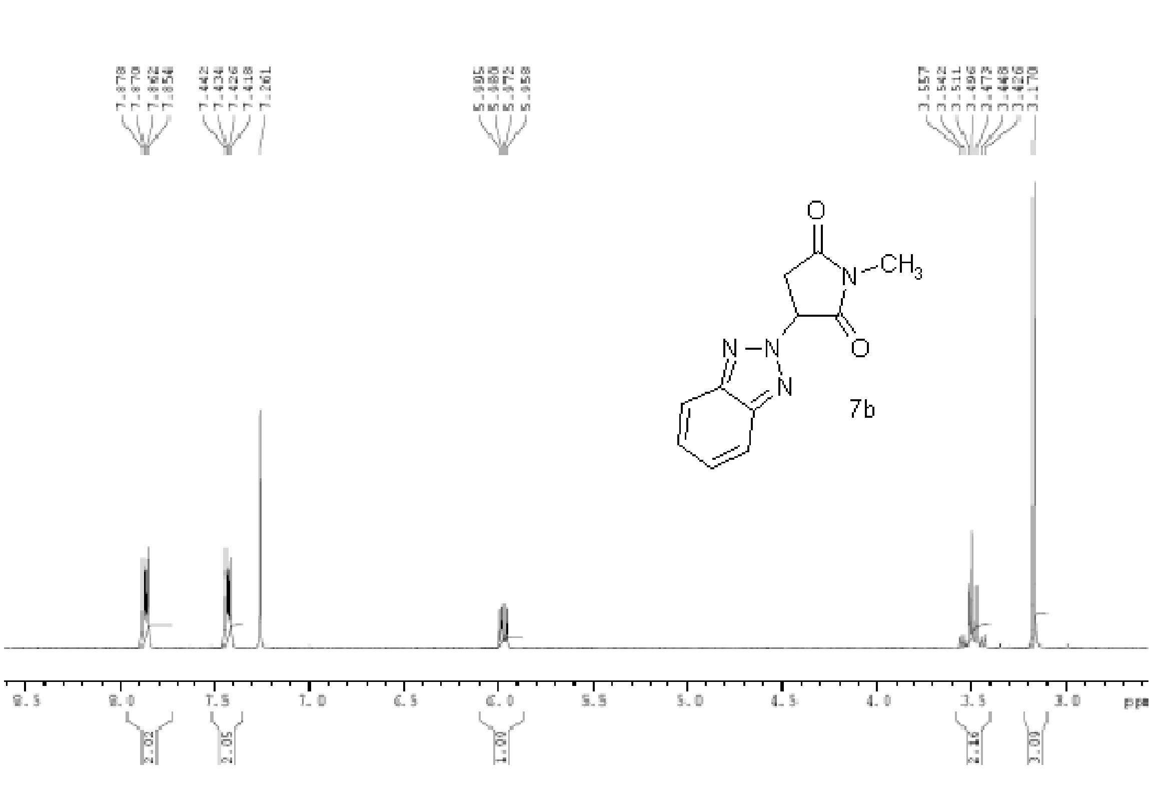

Figure S26. ^{13}C-NMR of **7b**.

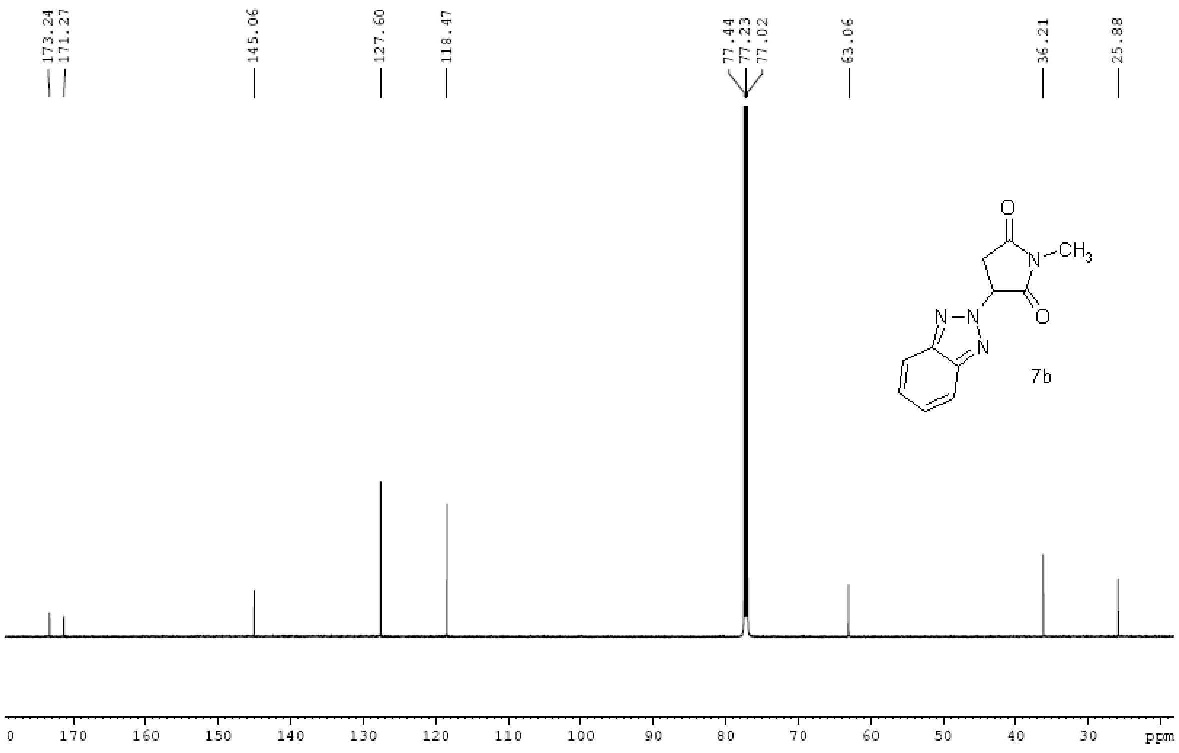

Figure S27. ^{1}H-NMR of **7c**.

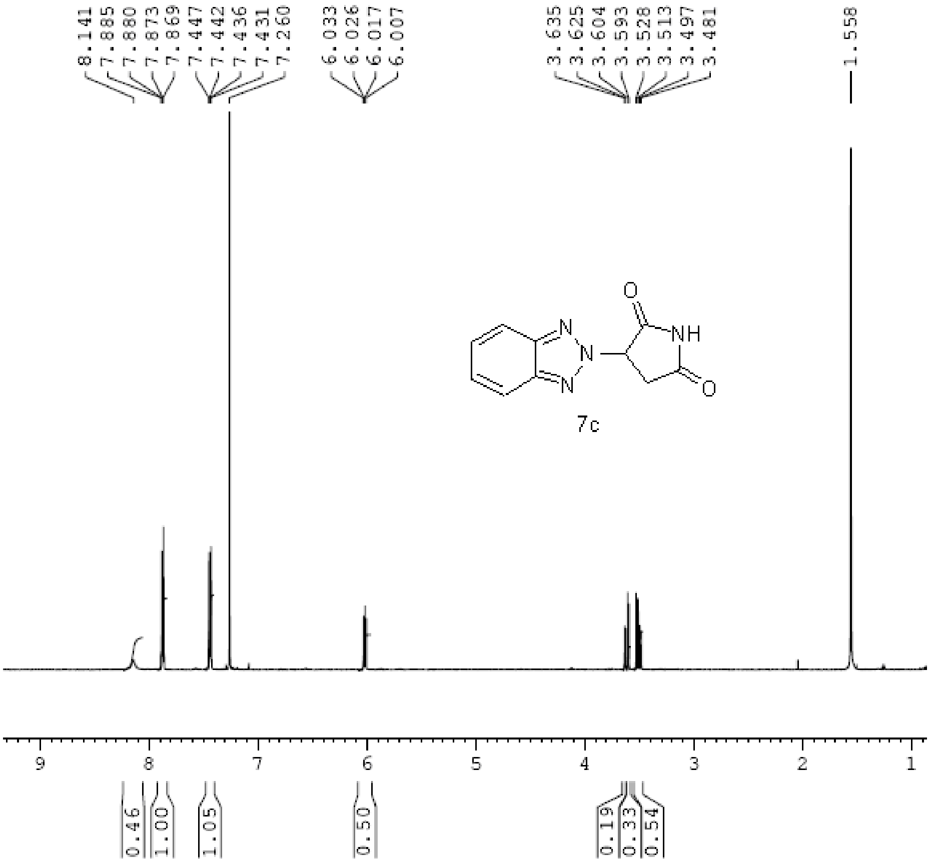

Figure S28. ^{13}C-NMR of **7c**.

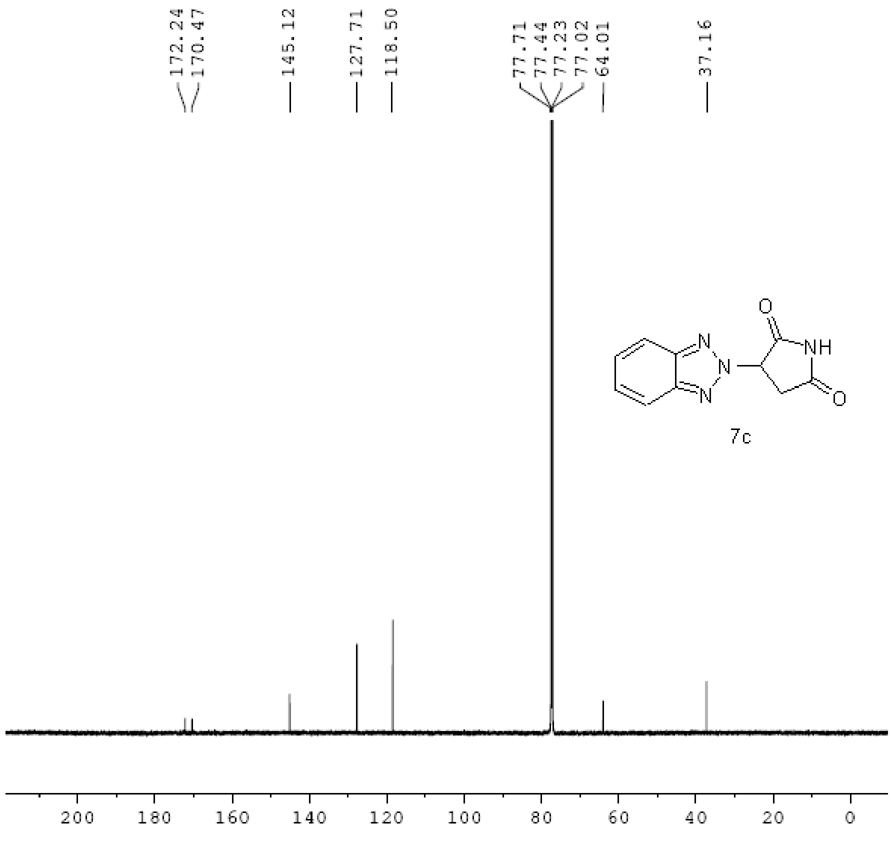

Figure S29. ^{1}H-NMR of **9a**.

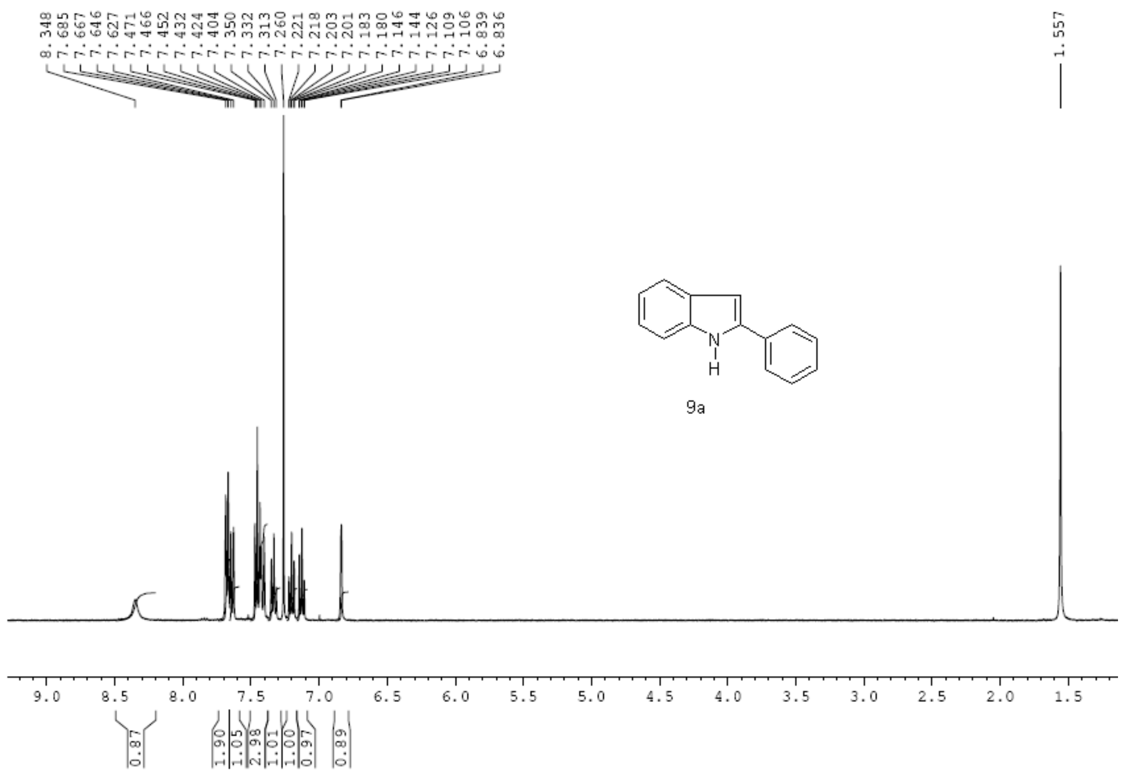

Figure S30. ^{13}C-NMR of **9a**.

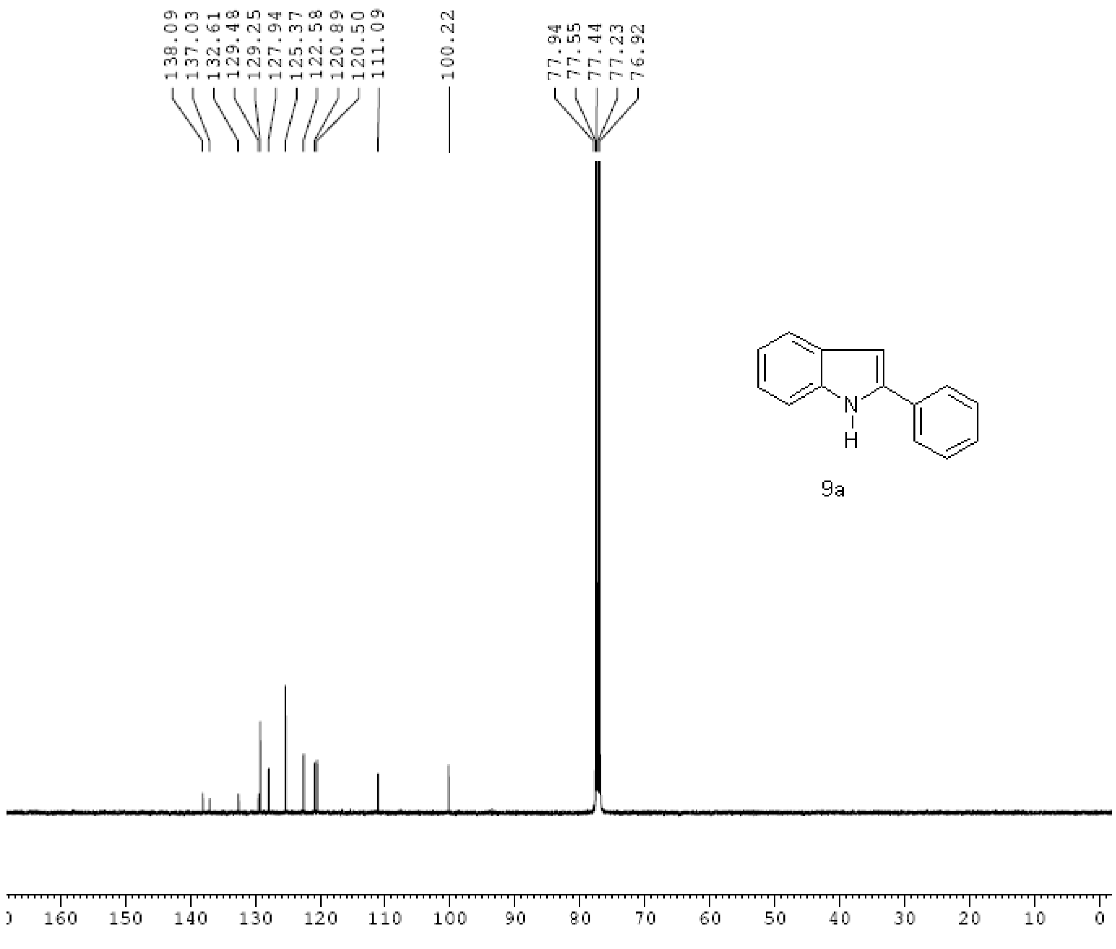

Figure S31. ^{1}H-NMR of **9b**.

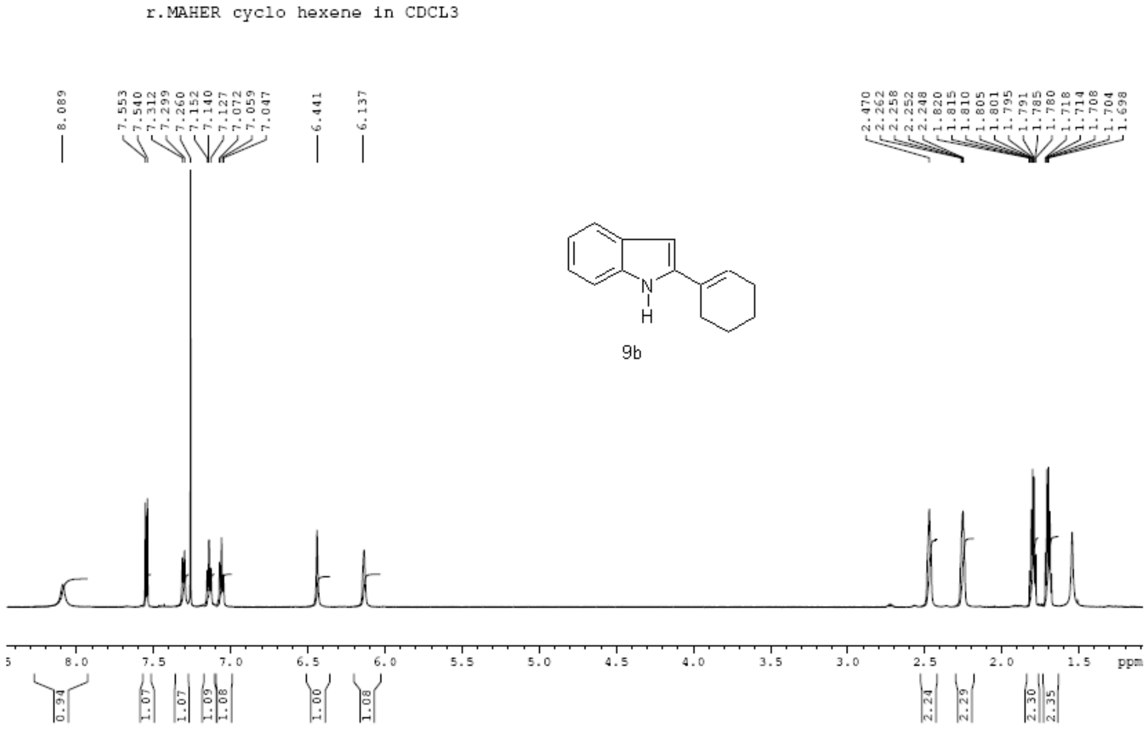

Figure S32. ^{13}C-NMR of **9b**.

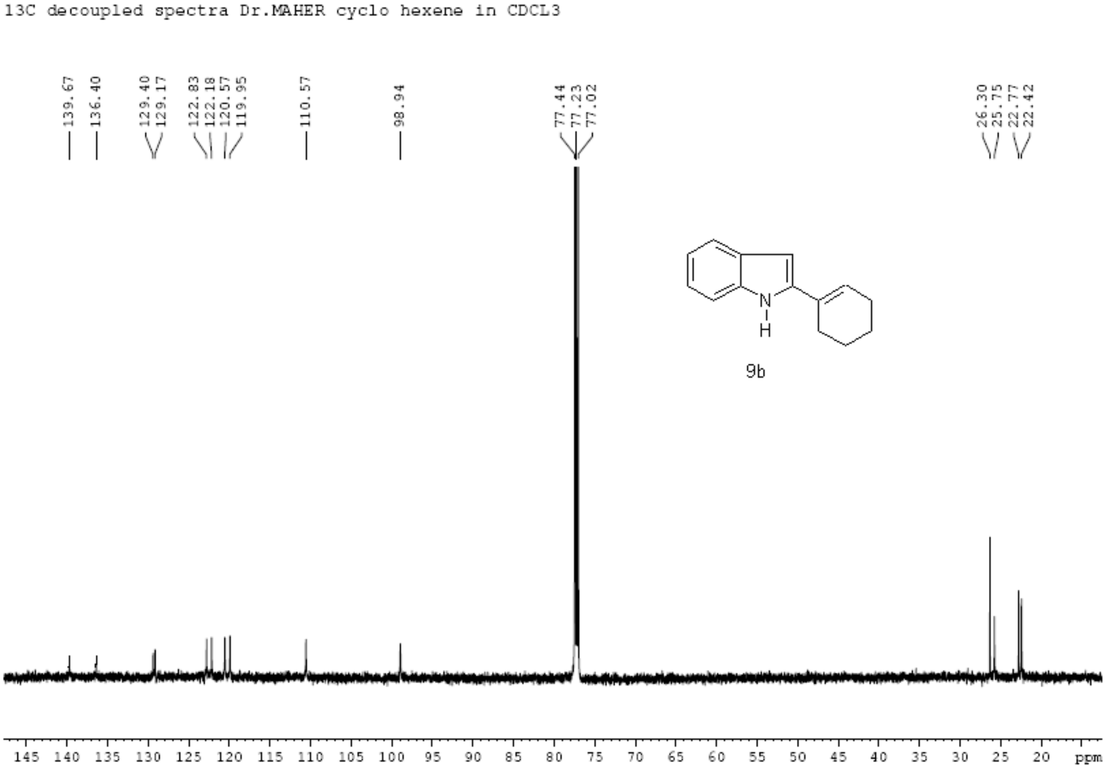

Figure S33. ^{1}H-NMR of **9c**.

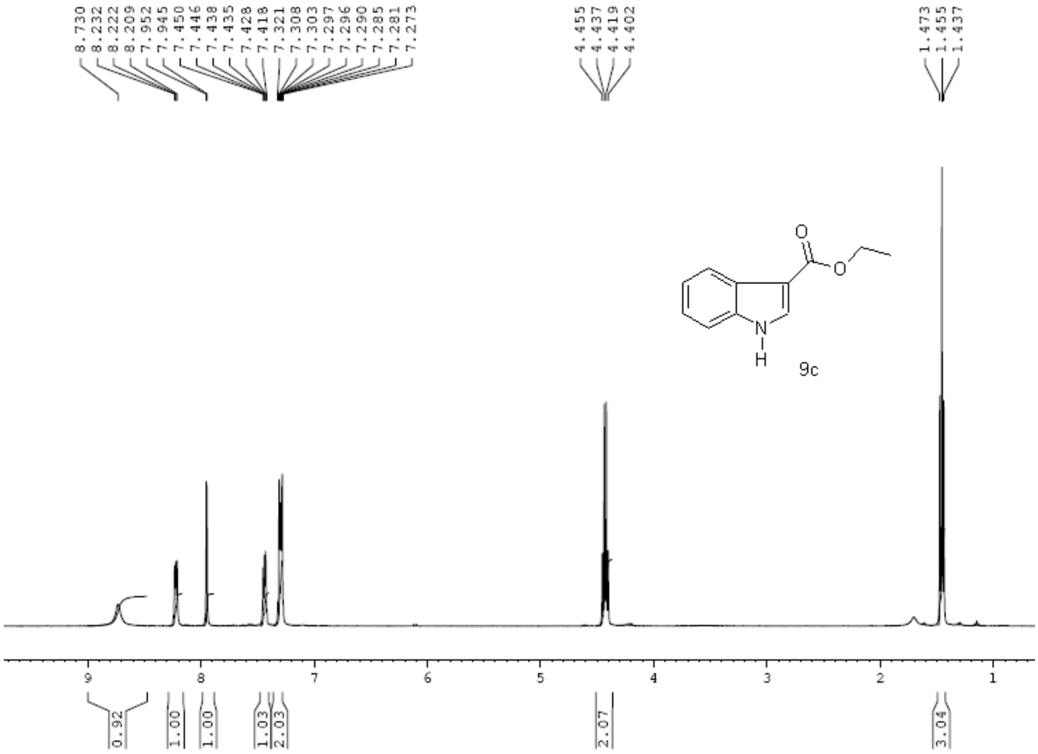

Figure S34. ^{13}C-NMR of **9c**.

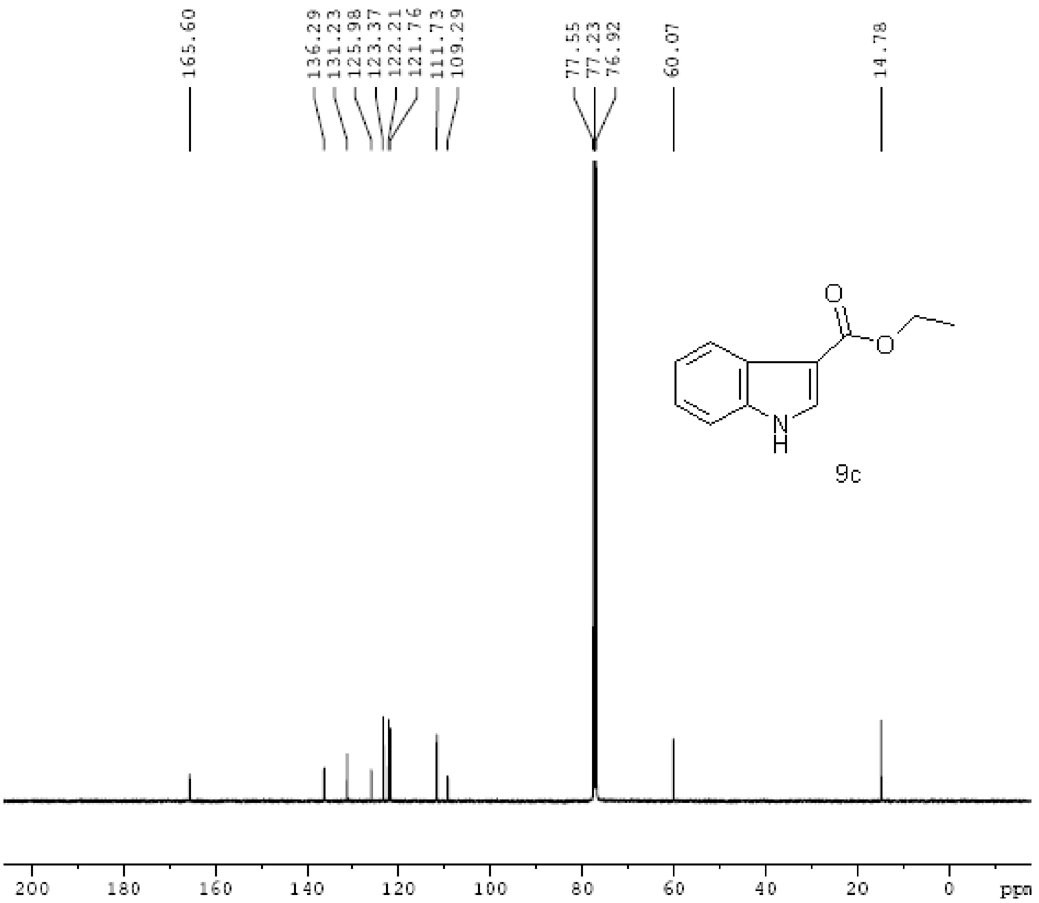

Figure S35. ^{1}H-NMR of **9d**.

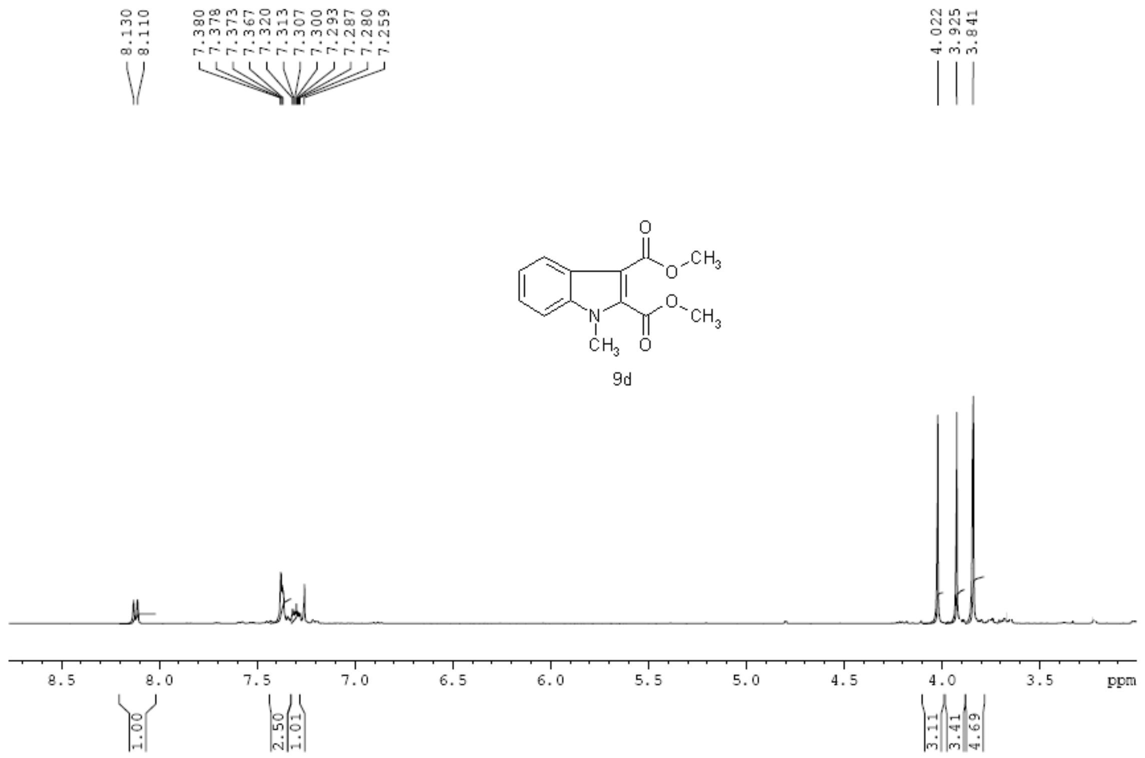

Figure S36. ^{13}C-NMR of **9d**.

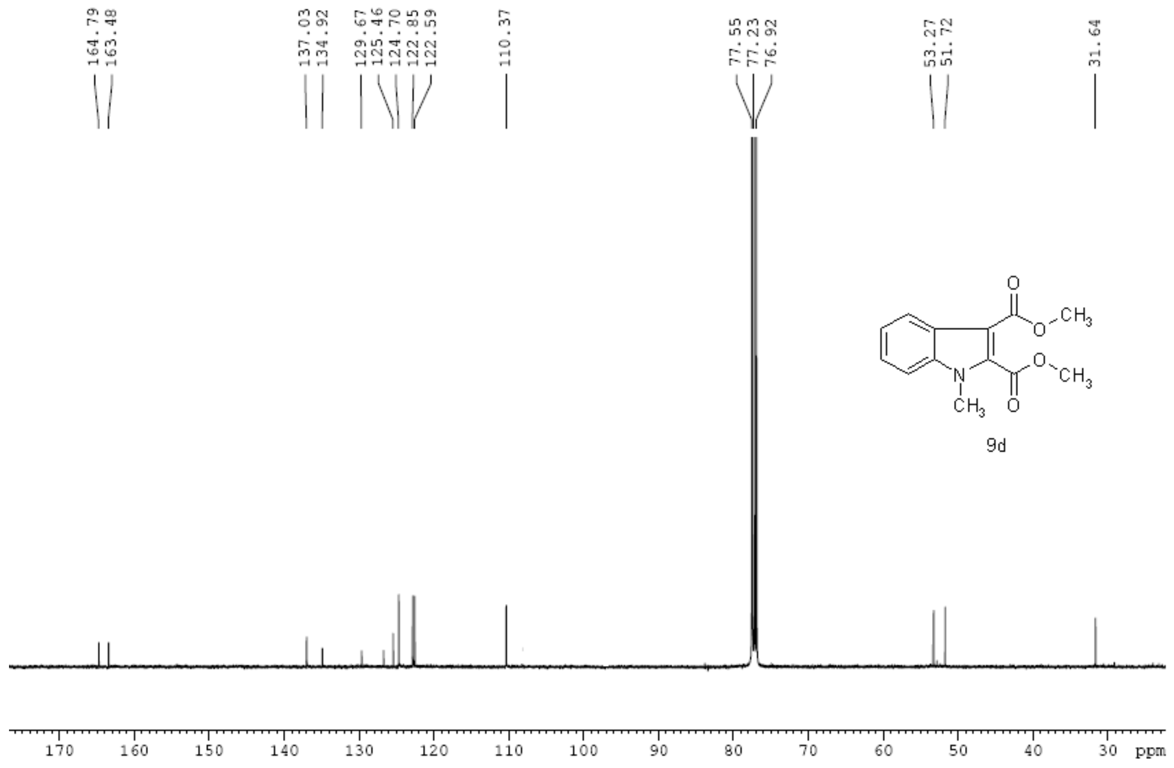

Figure S37. ^{1}H-NMR of **9e**.

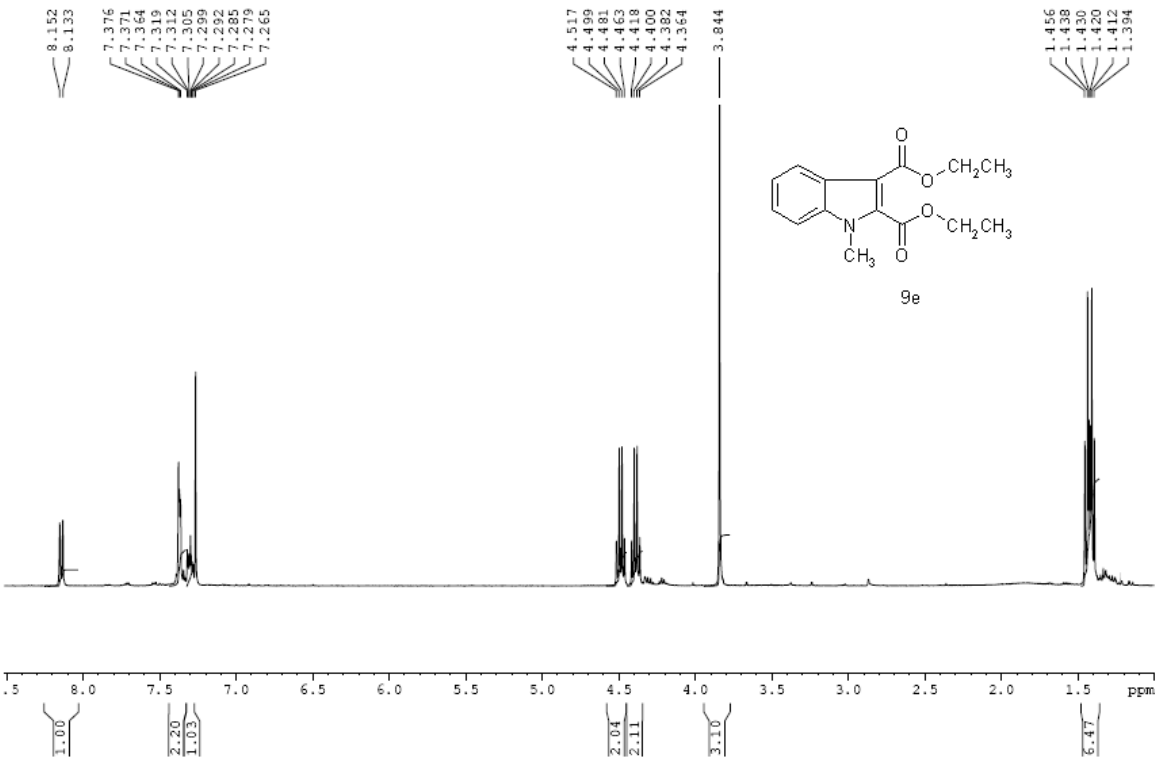

Figure S38. ^{13}C-NMR of **9e**.

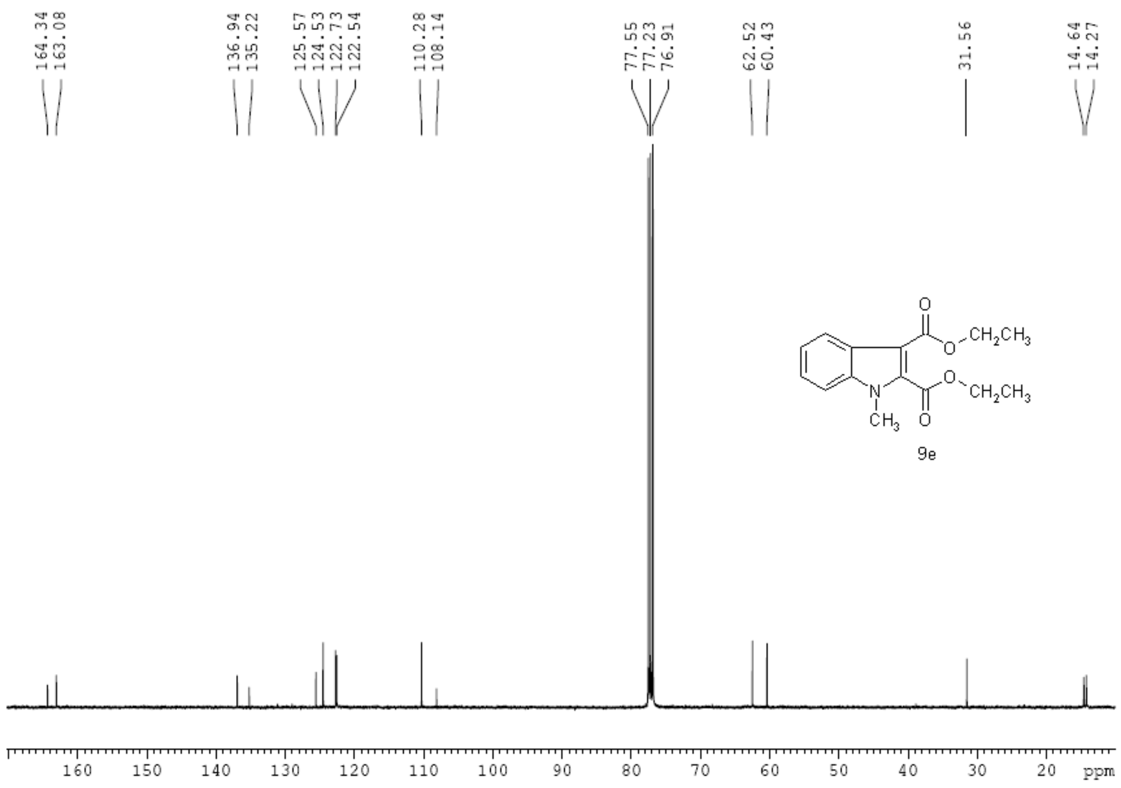

Figure S39. ^{1}H-NMR of **10a**.

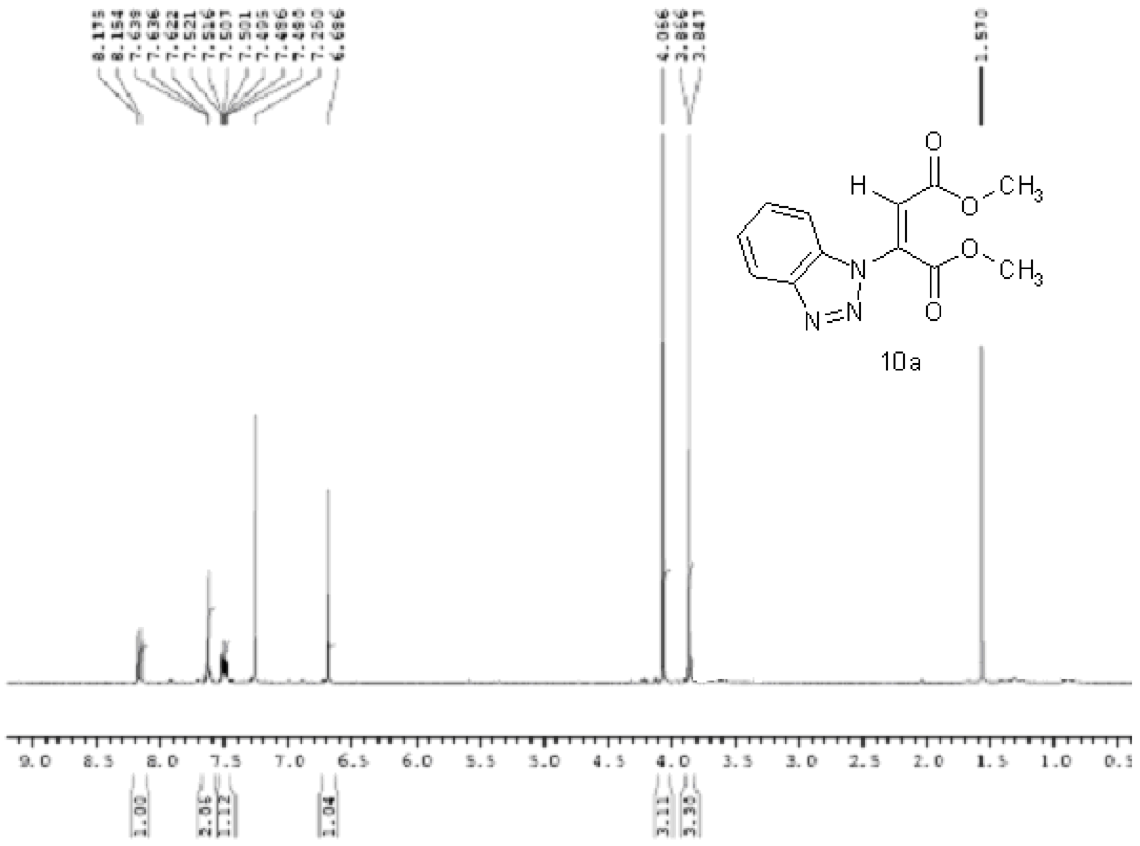

Figure S40. ^{13}C-NMR of **10a**.

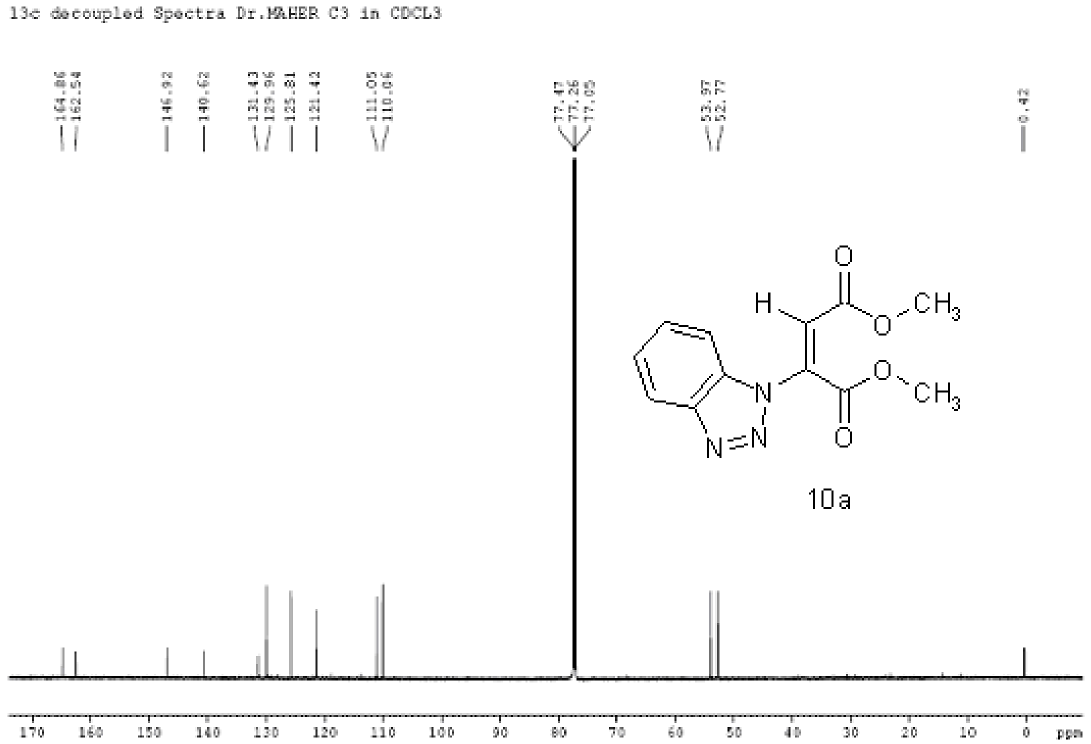

Figure S41. ^{1}H-NMR of **10b**.

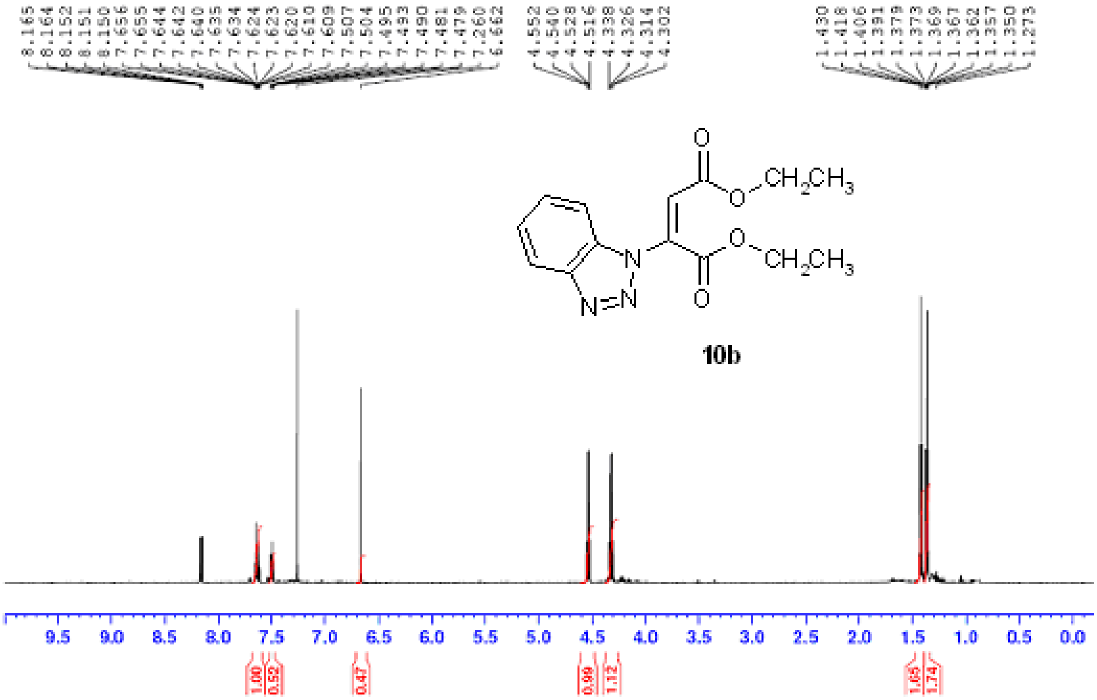

Figure S42. ^{13}C-NMR of **10b**.

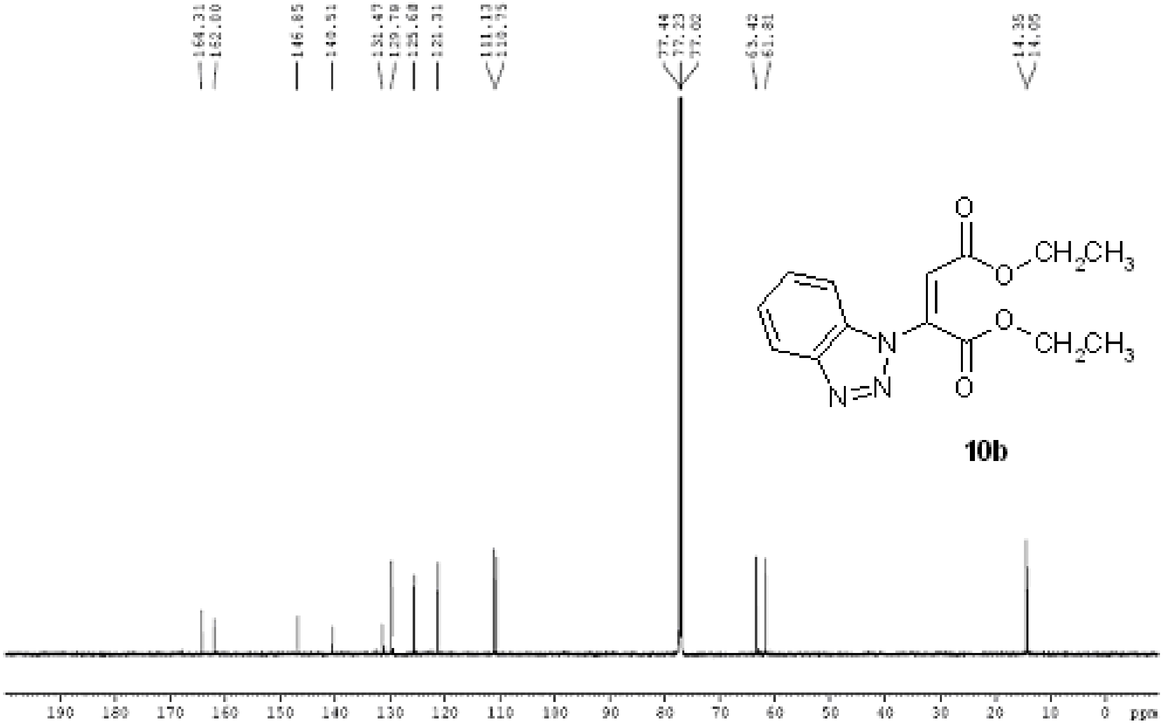

Figure S43. ^{1}H-NMR of **11a**.

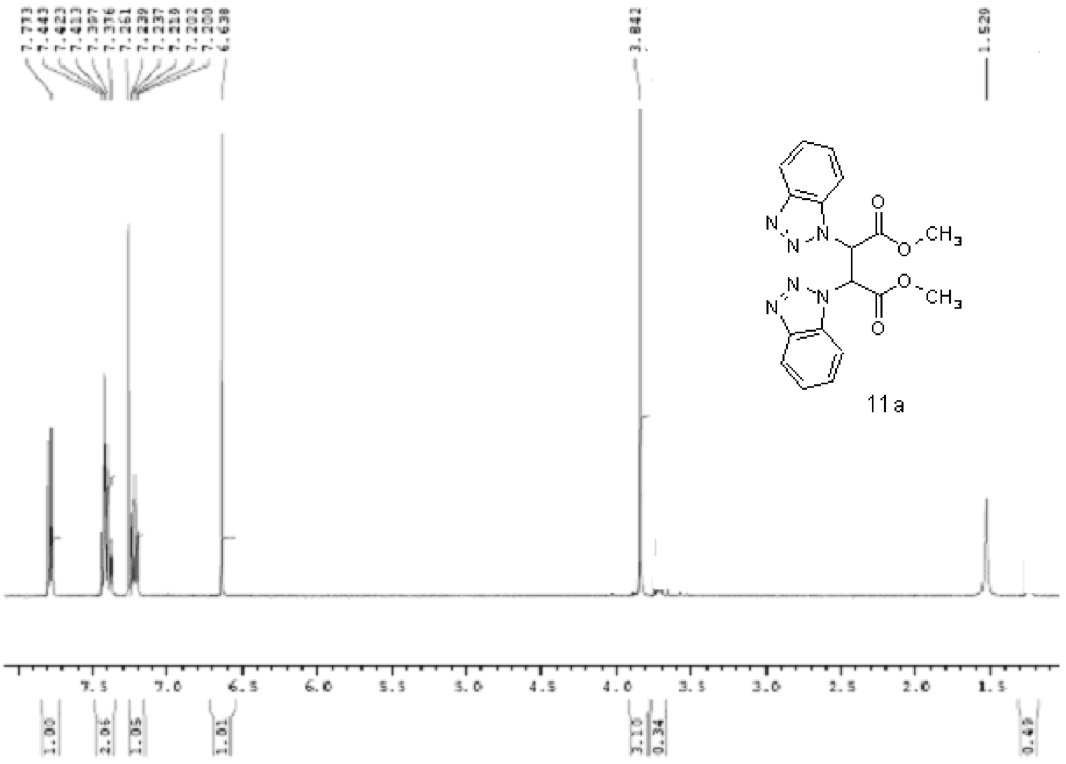

Figure S44. ^{13}C-NMR of **11a**.

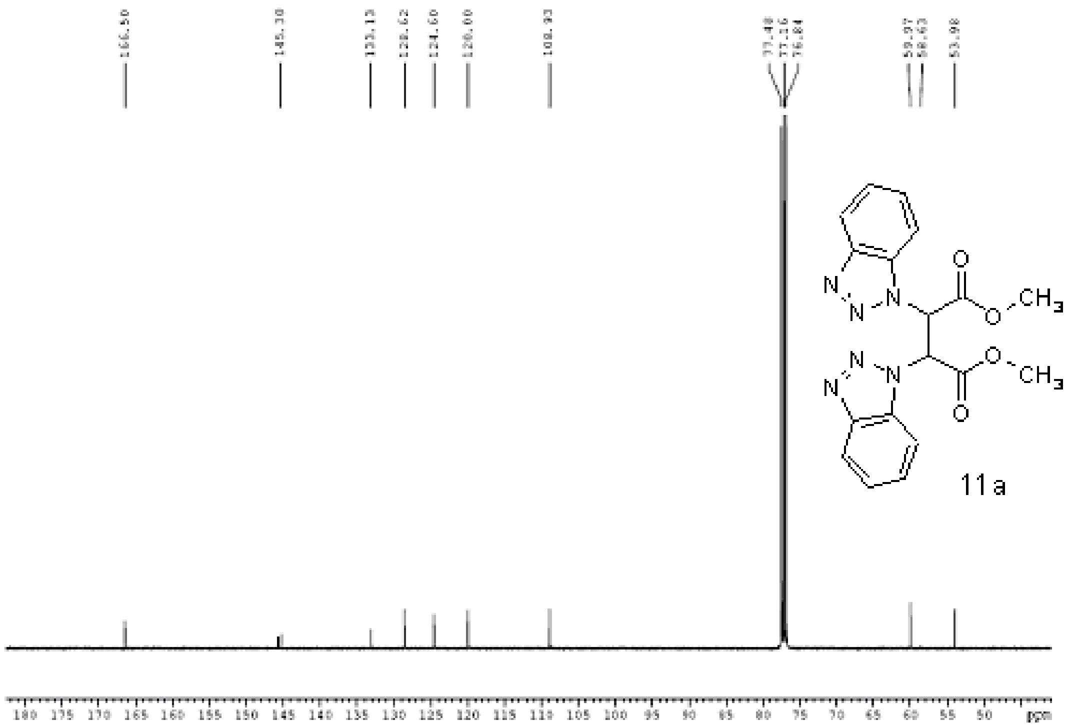

Figure S45. ^{1}H-NMR of **11b**.

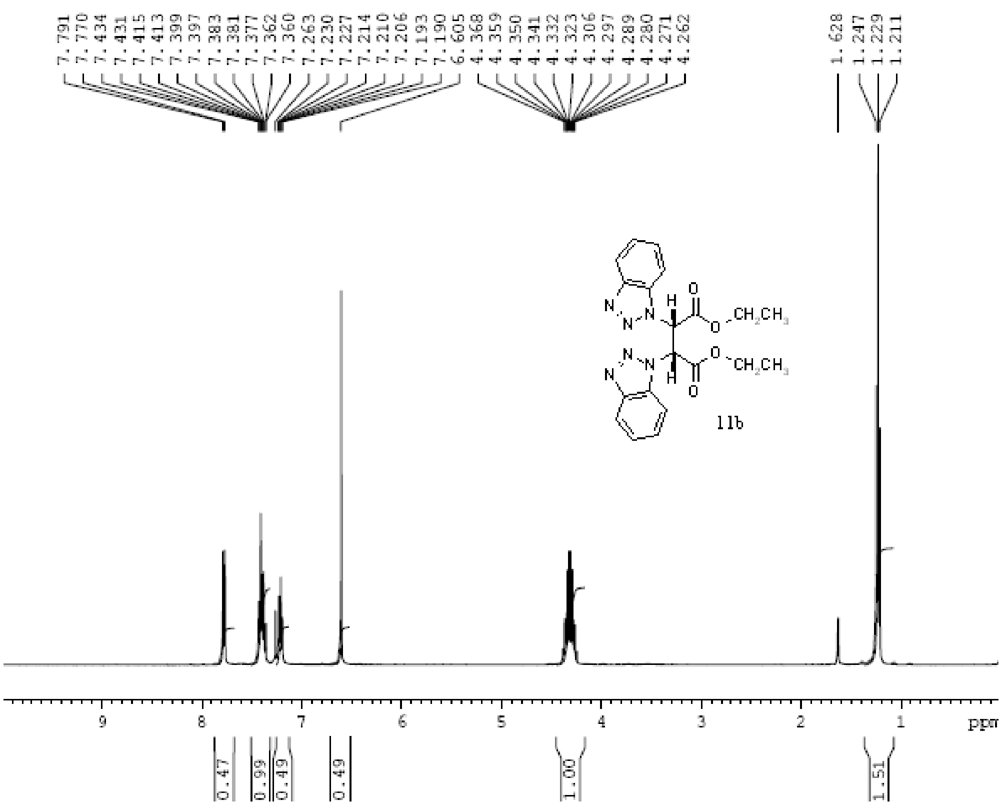

Figure S46. ^{13}C-NMR of **11b**.

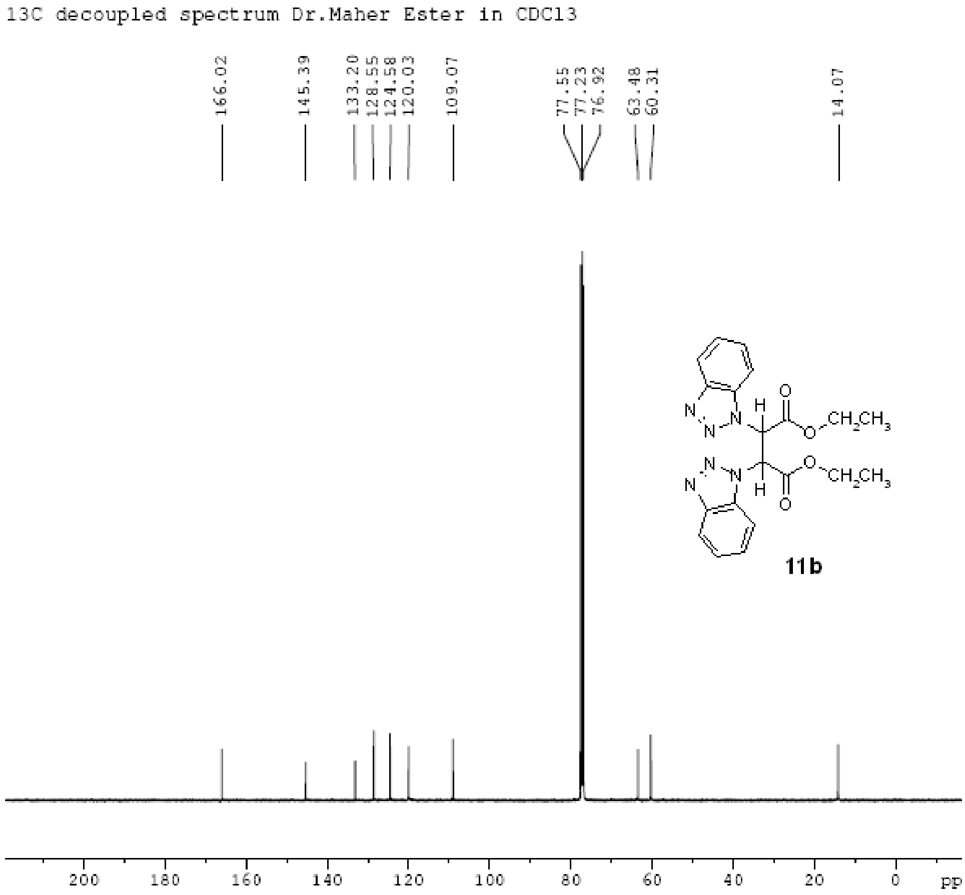

Figure S47. ^{1}H-NMR of **12**.

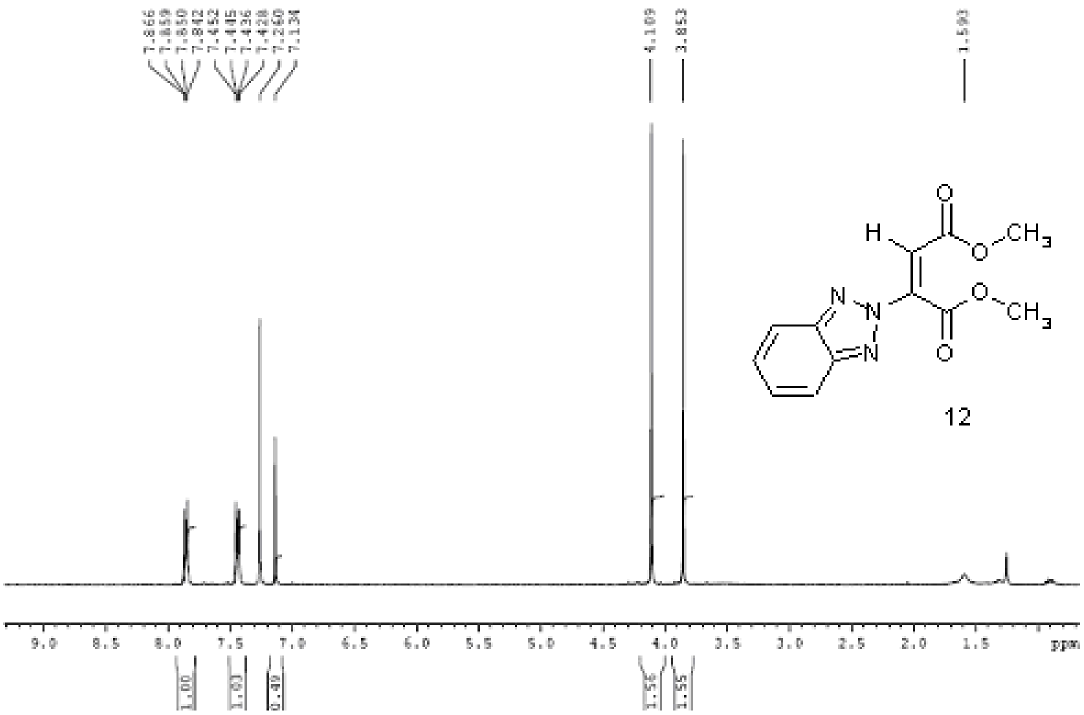

Figure S48. ^{13}C-NMR of **12**.

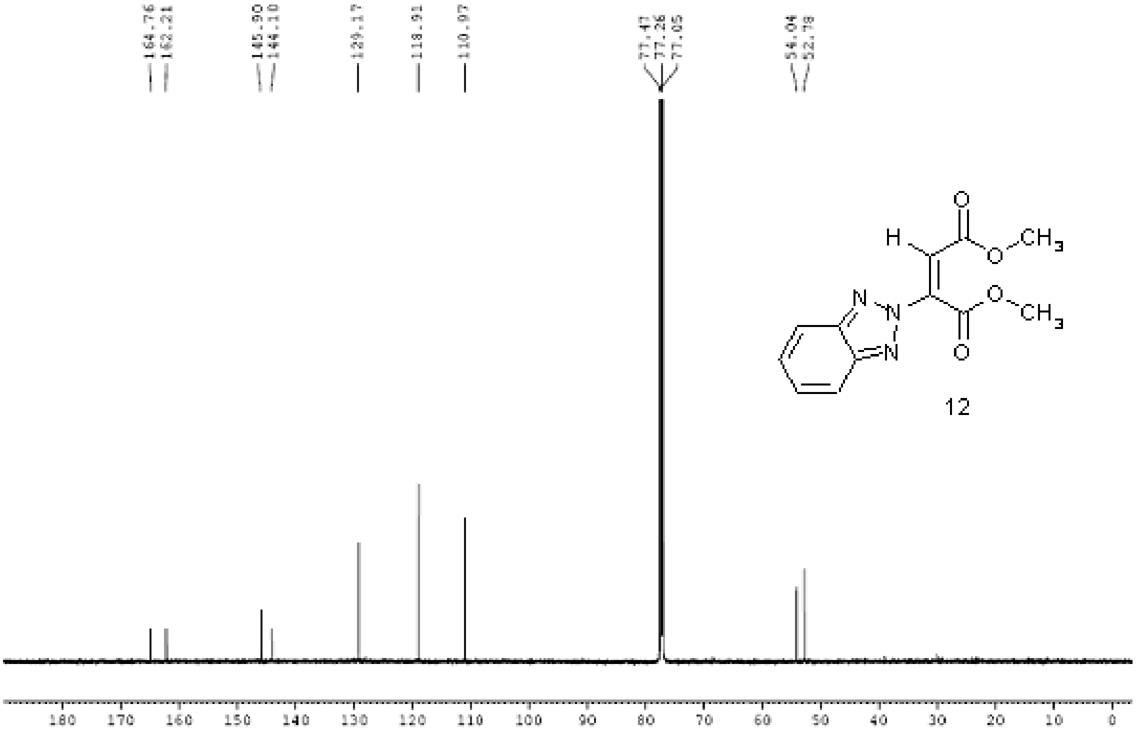

Figure S49. GC-Mass of **4a**.

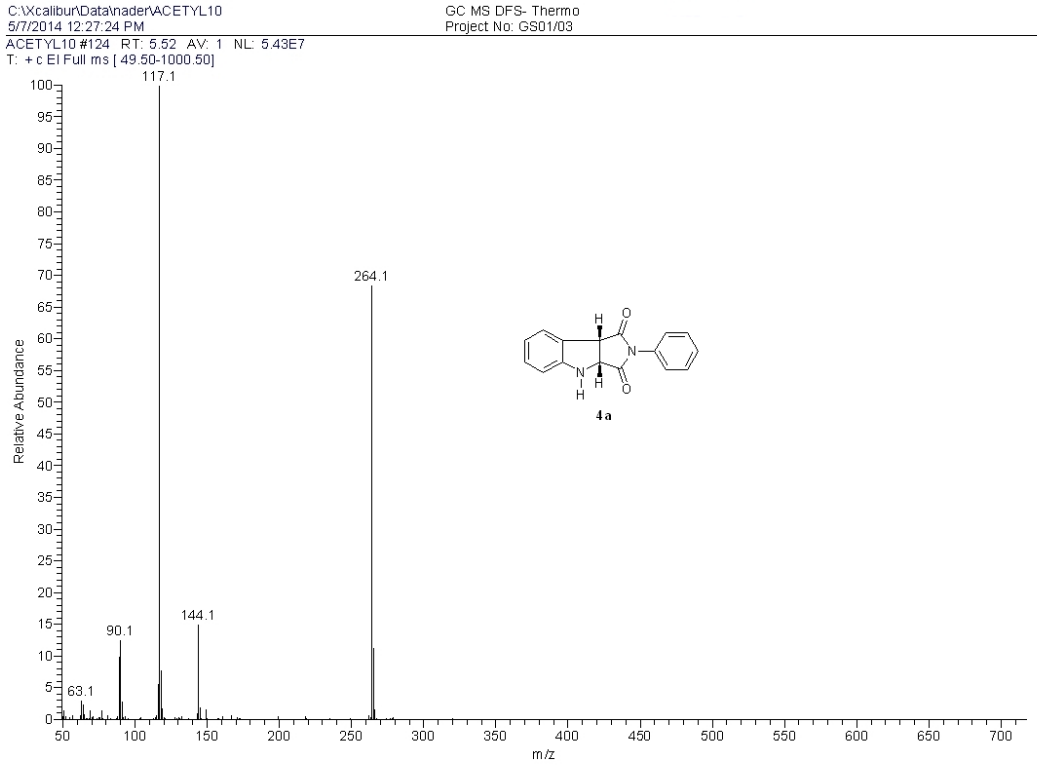

Figure S50. GC-Mass of **4b**.

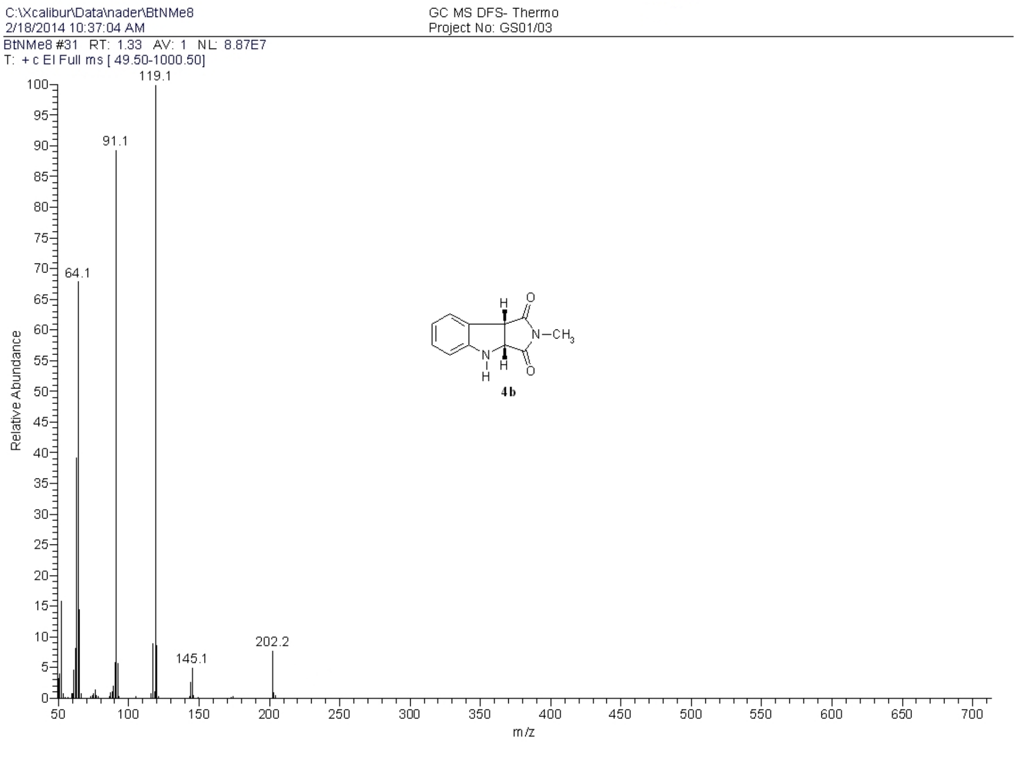

Figure S51. GC-Mass of **4d**.

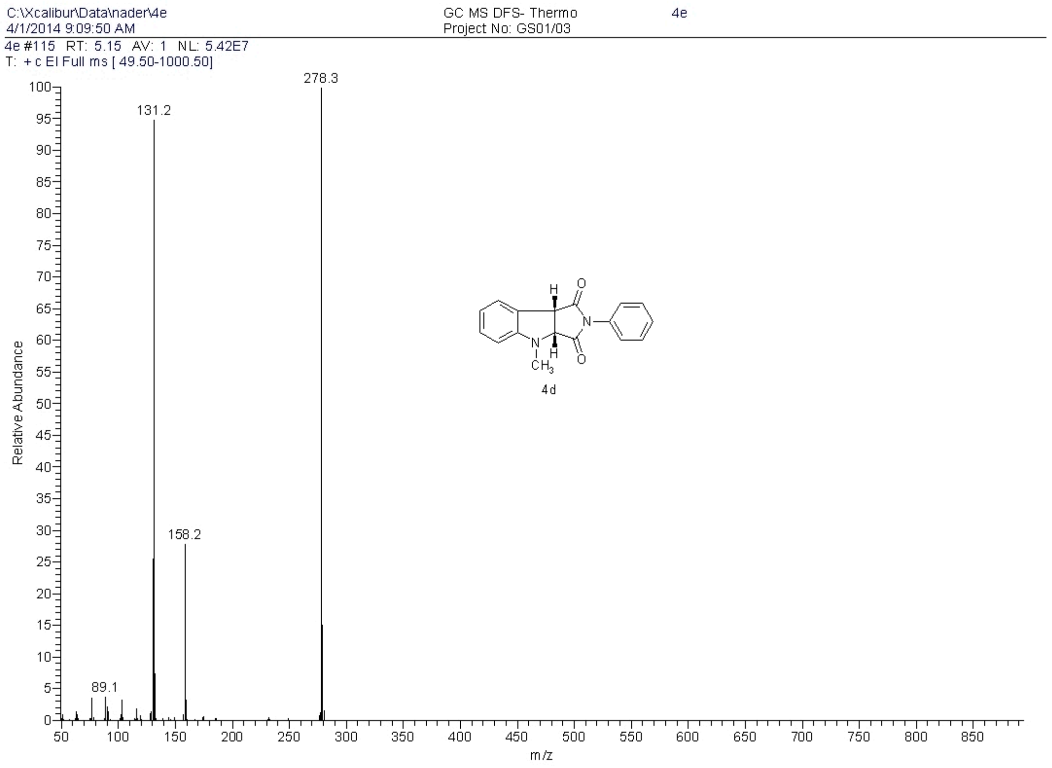

Figure S52. GC-Mass of **4e**.

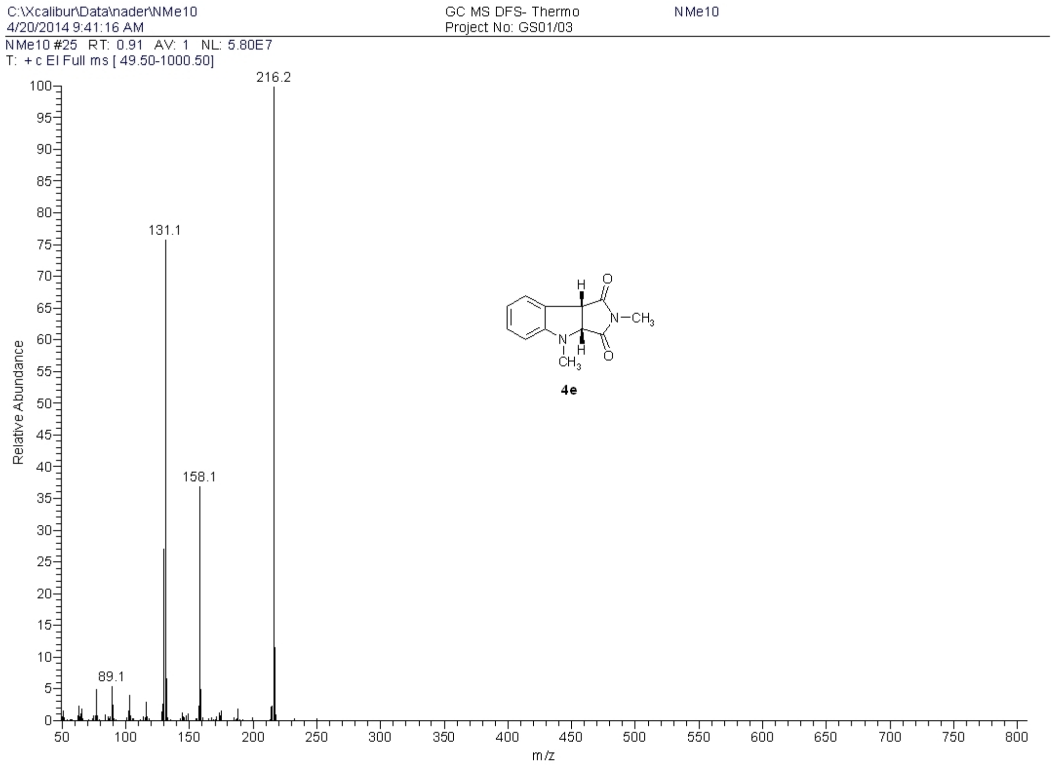

Figure S53. GC-Mass of **4g**.

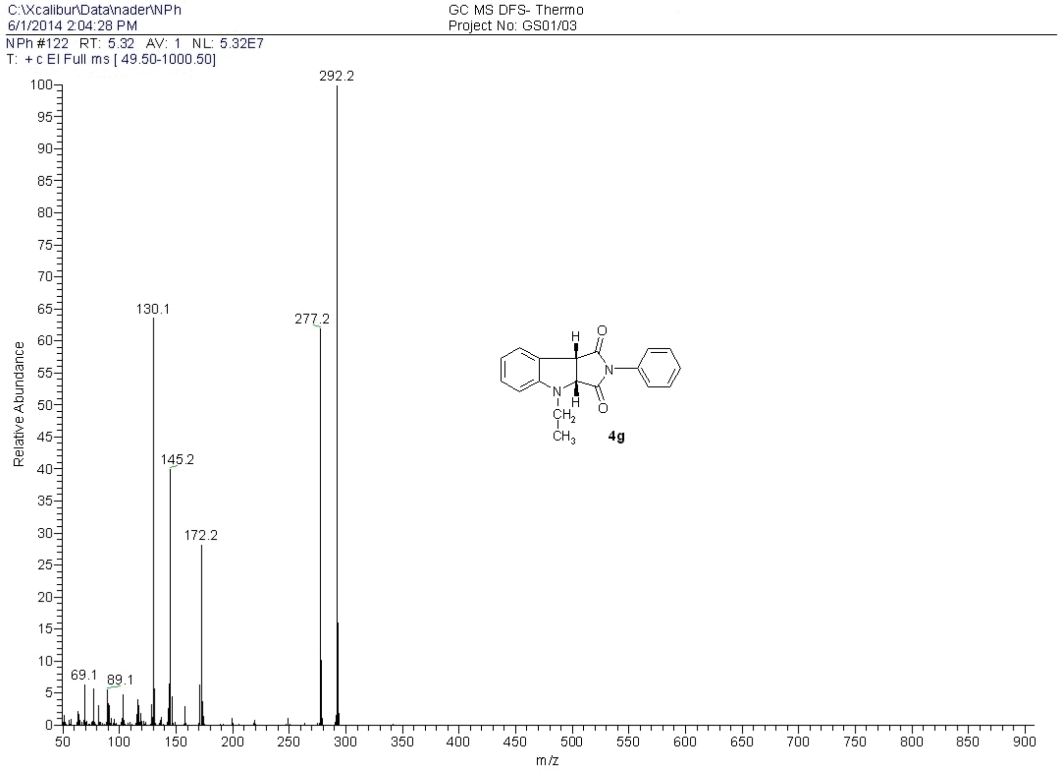

Figure S54. GC-Mass of **4h**.

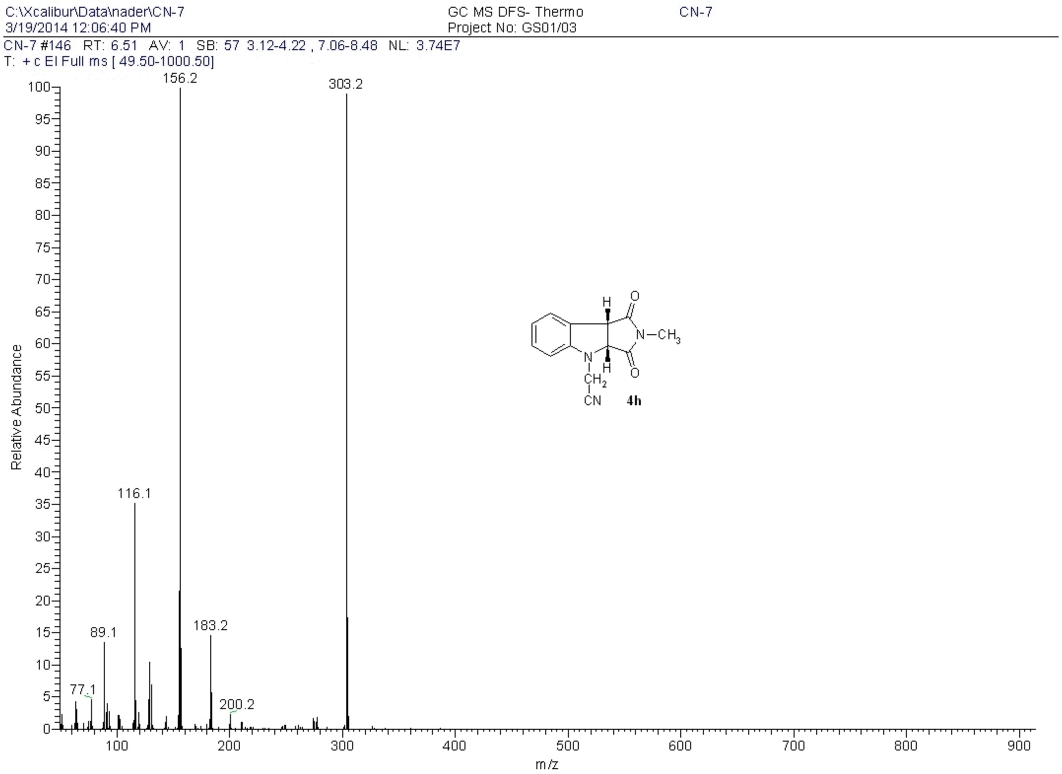

Figure S55. GC-Mass of **4i**.

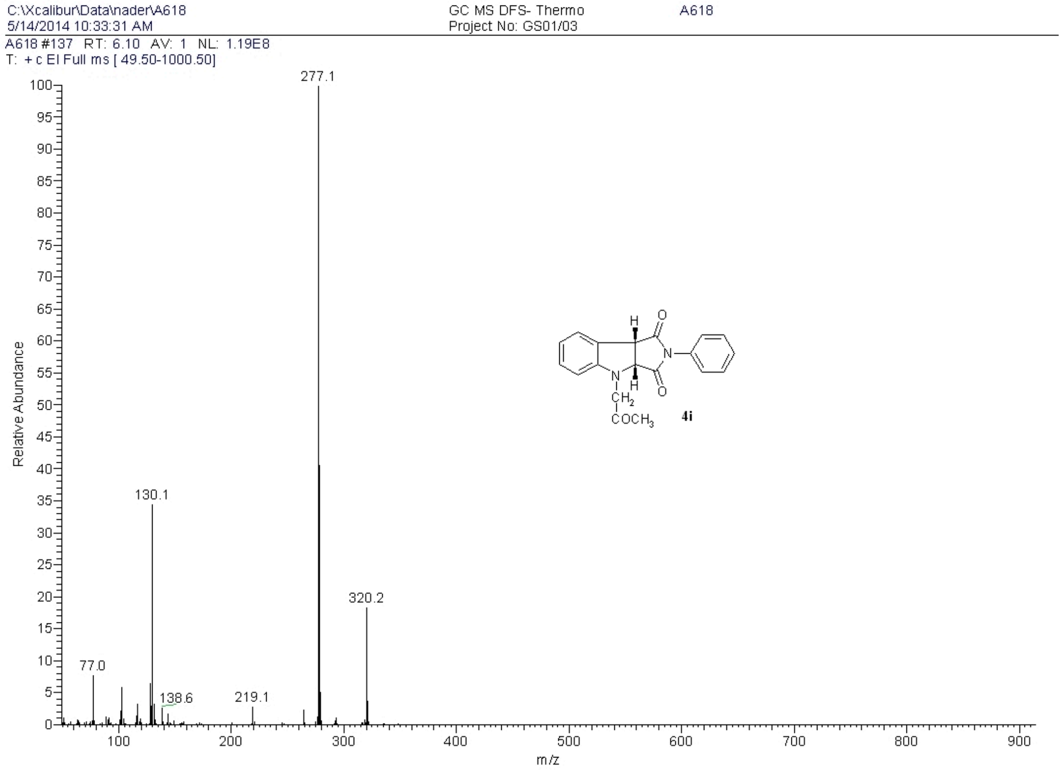

Figure S56. GC-Mass of **5**.

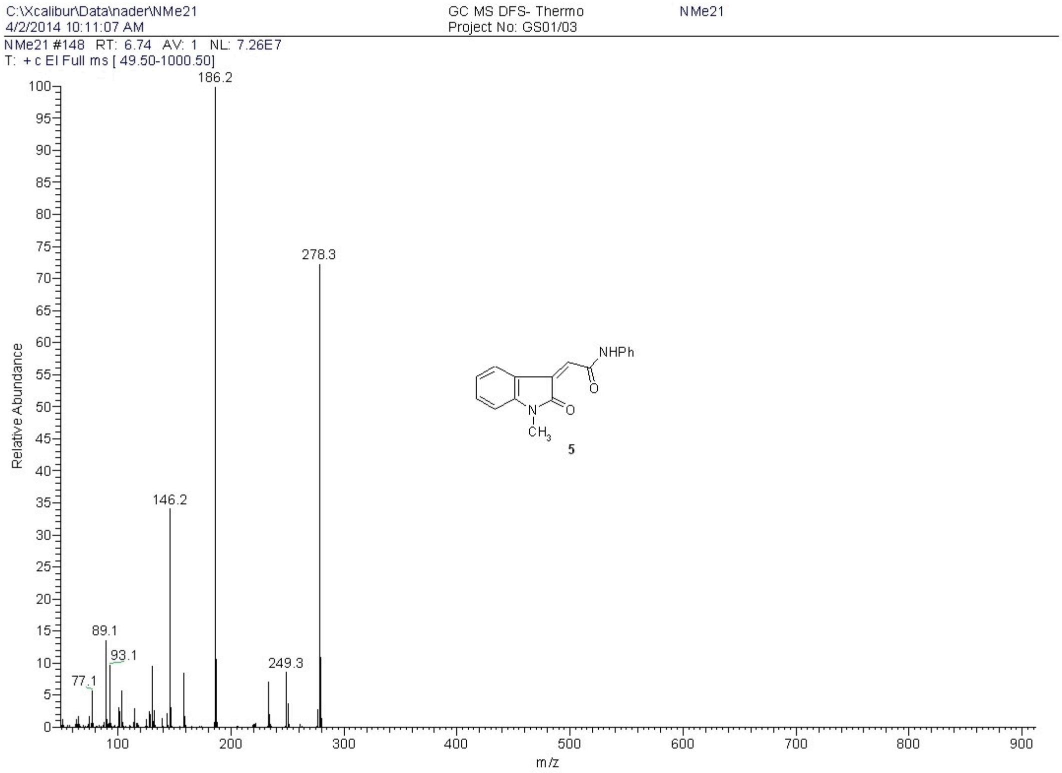

Figure S57. GC-Mass of **6a**.

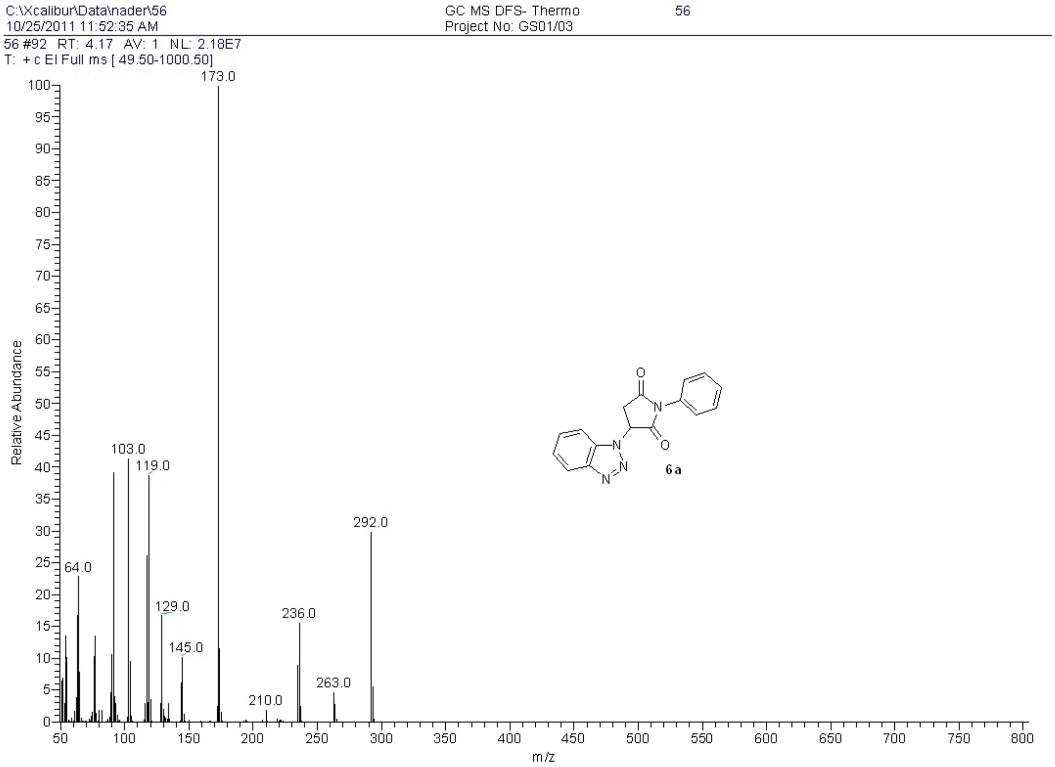

Figure S58. GC-Mass of **6b**.

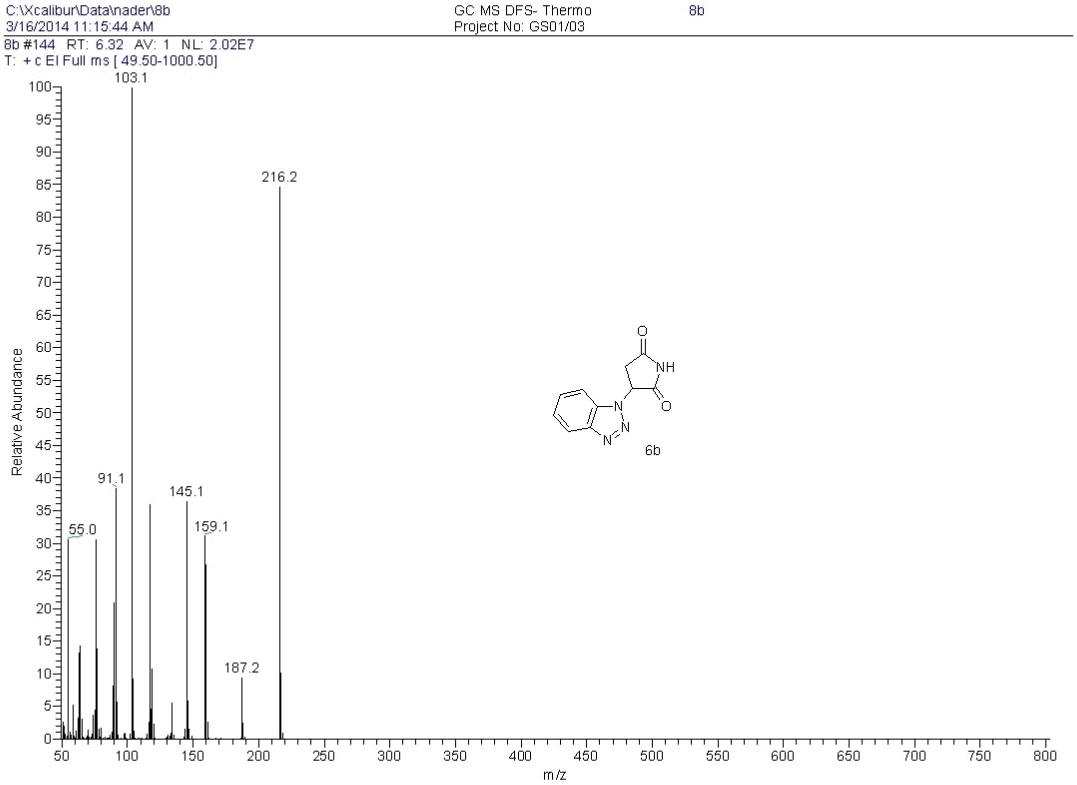

Figure S59. GC-Mass of **7a**.

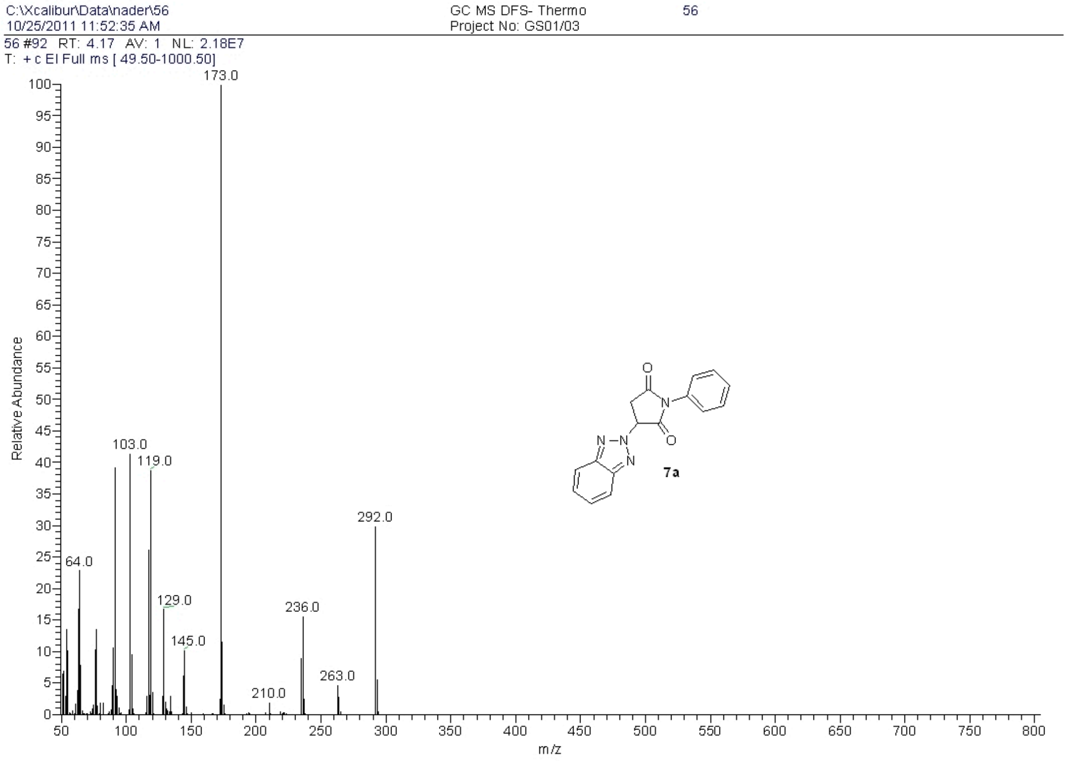

Figure S60. GC-Mass of **7b**.

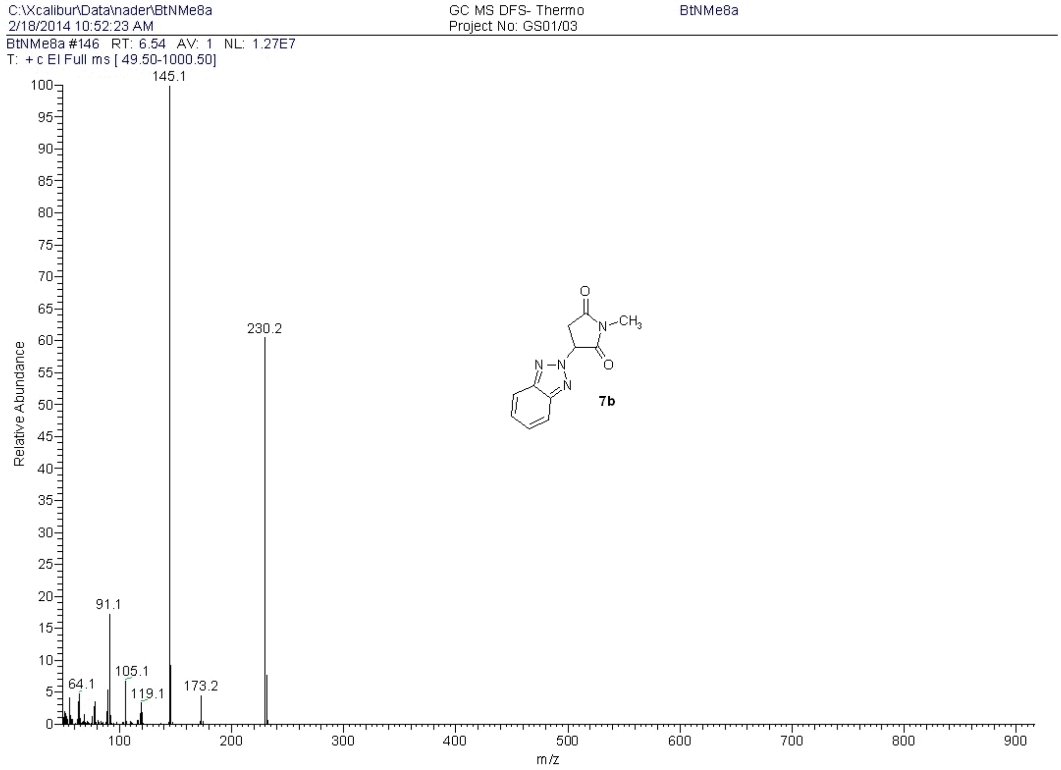

Figure S61. GC-Mass of **9d**.

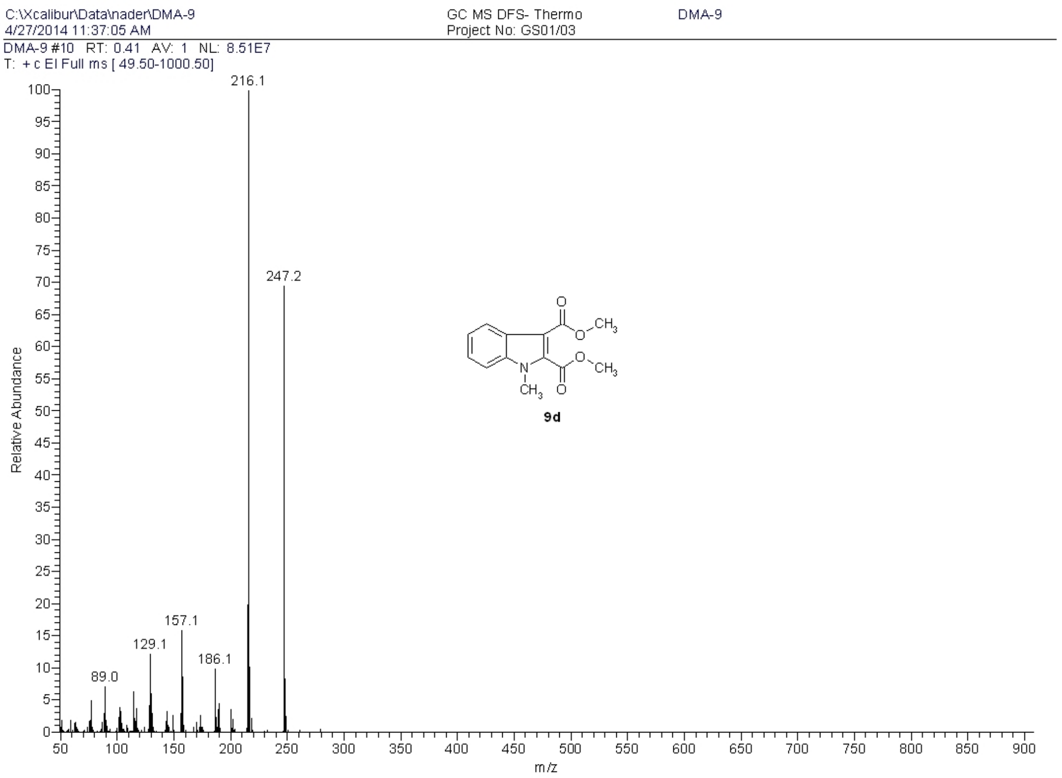

Figure S62. GC-Mass of **9e**.

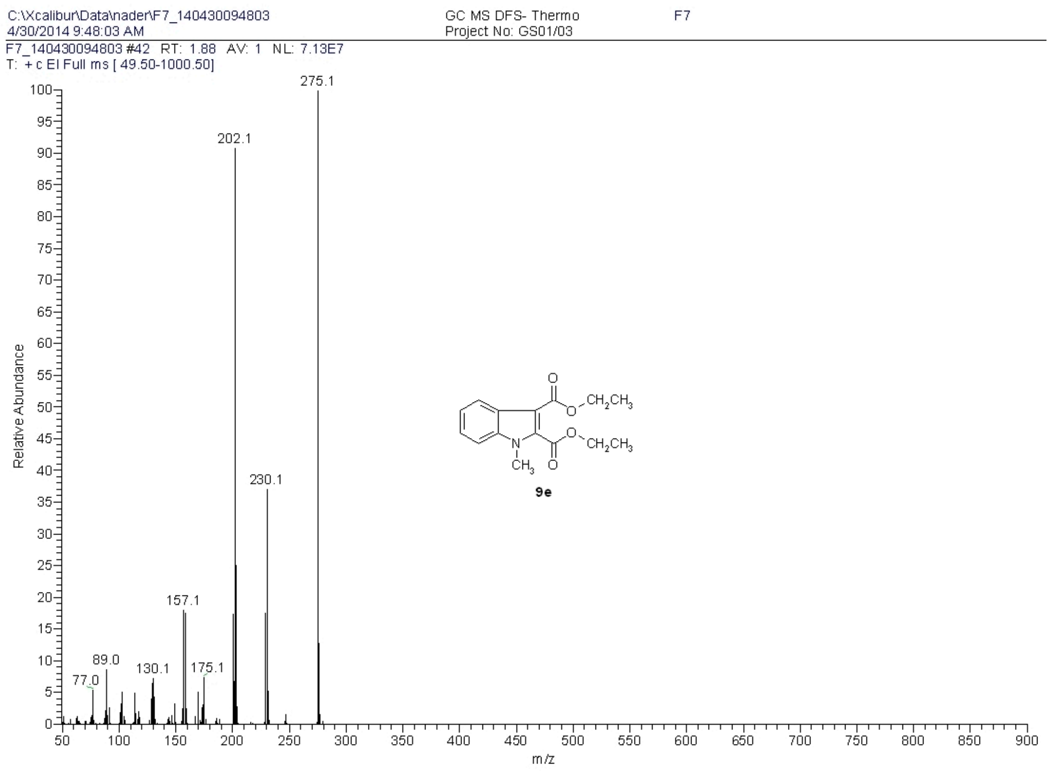

Figure S63. GC-Mass of **11a**.

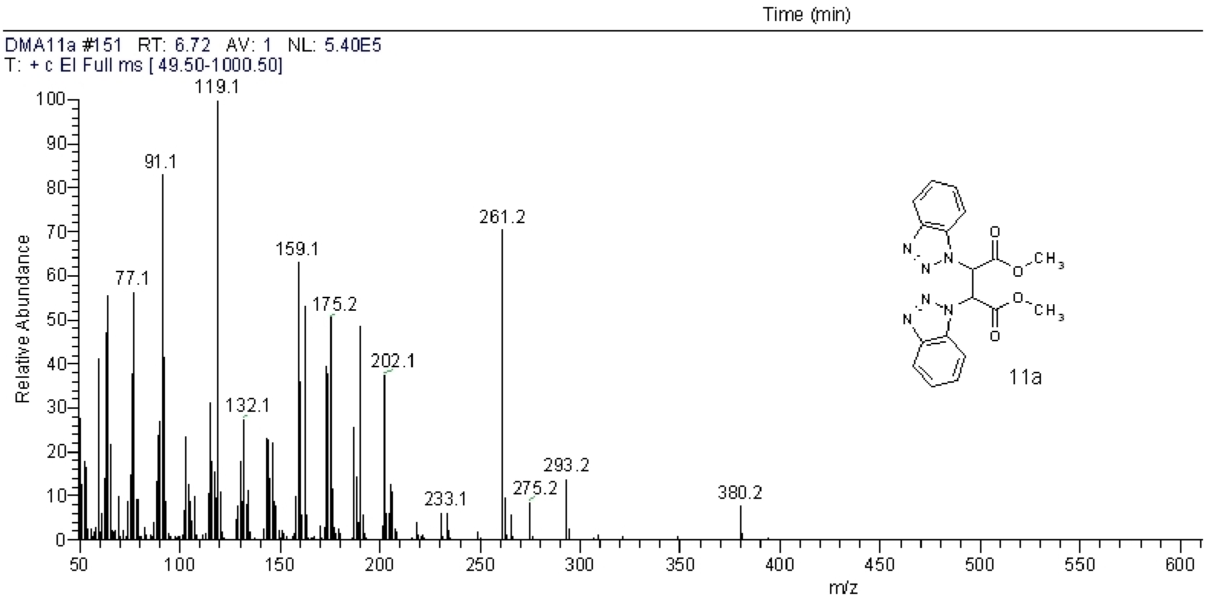

Figure S64. GC-Mass of **11b**.

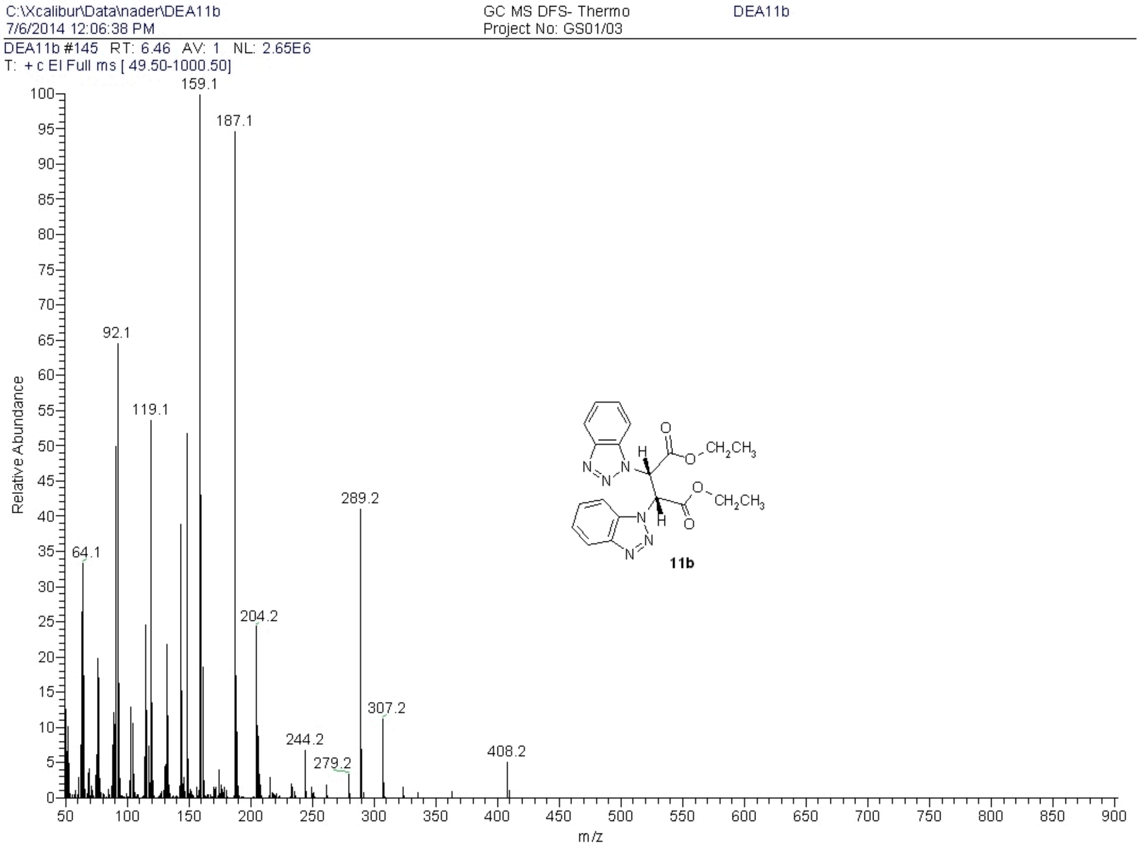

Figure S65. X-Ray Crystal Structure of **4c**.

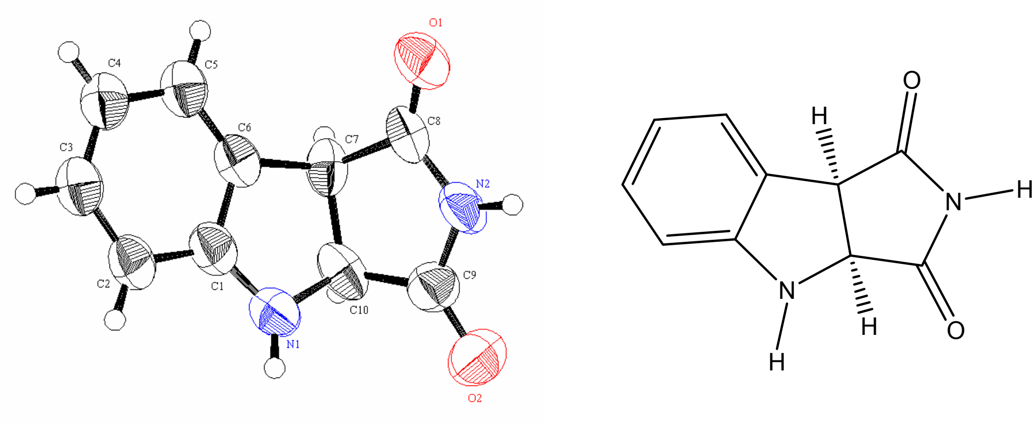

4c

Figure S66. X-Ray Crystal Structure of **4d**.

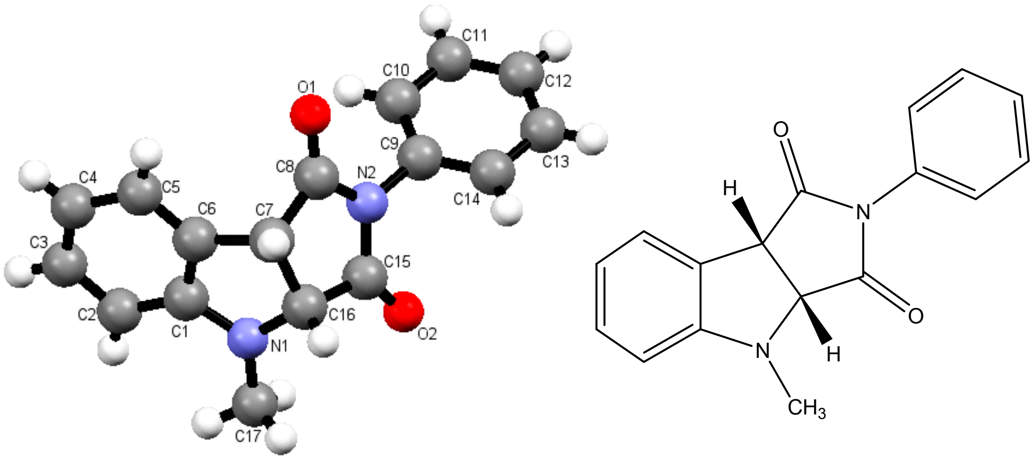

4d

Figure S67. X-Ray Crystal Structure of **4g**.

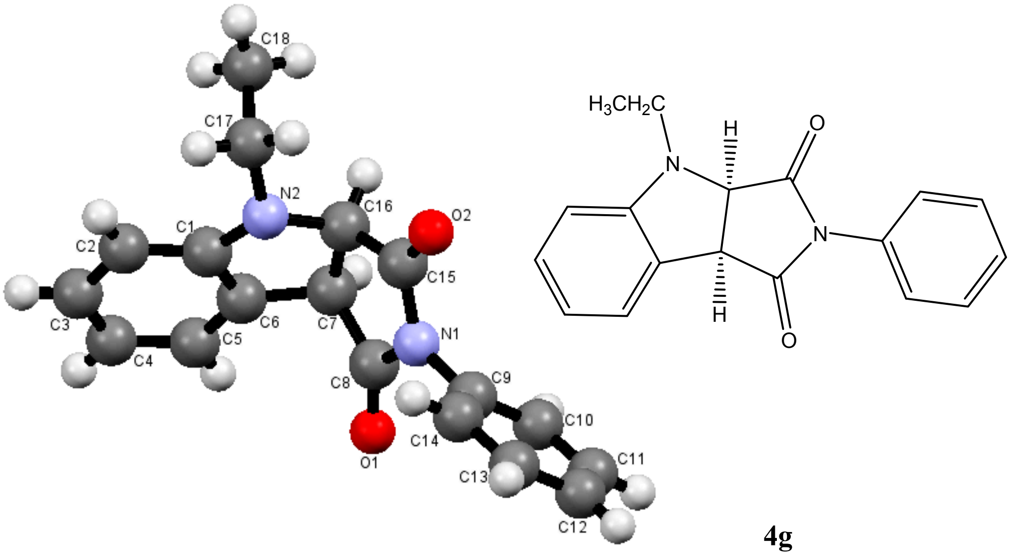

4g

Figure S68. X-Ray Crystal Structure of **5**.

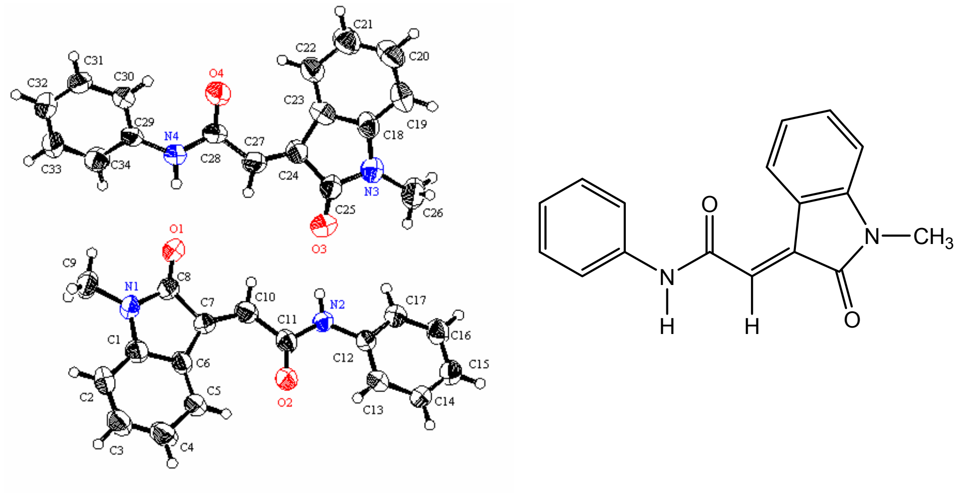

5

Figure S69. X-Ray Crystal Structure of **6b**.

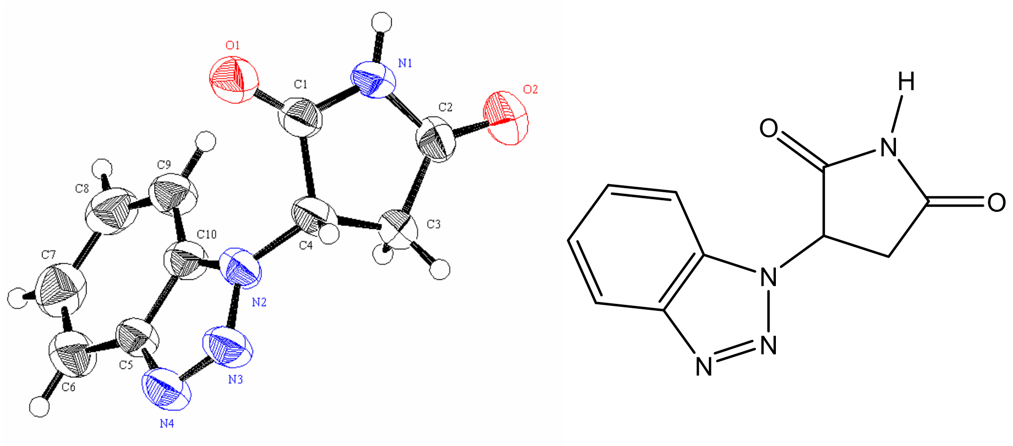

6b

Figure S70. X-Ray Crystal Structure of **11a**.

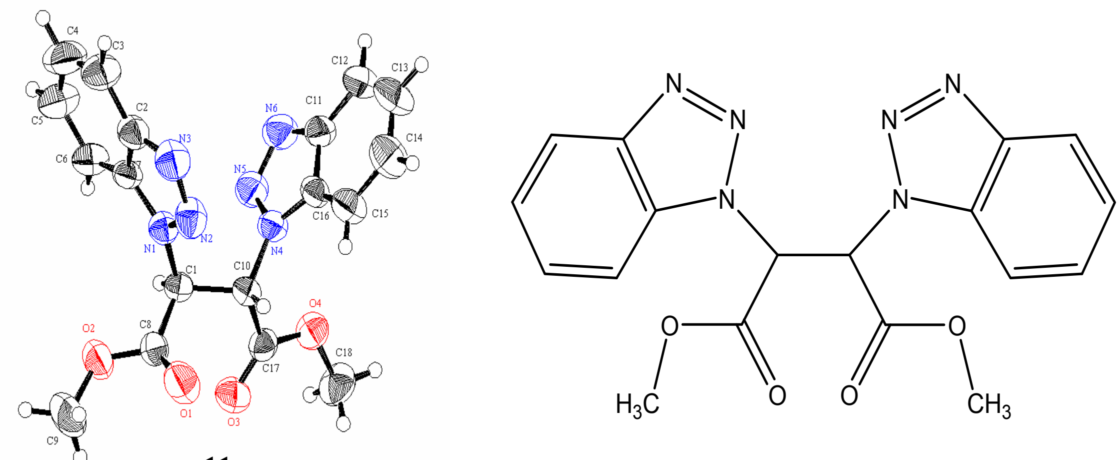

11a
